# Uncovering the mechanism of the effects of *Paeoniae Radix* Alba on iron-deficiency anaemia through a network pharmacology-based strategy

**DOI:** 10.1186/s12906-020-02925-4

**Published:** 2020-04-28

**Authors:** Xian-wen Ye, Ya-ling Deng, Lan-ting Xia, Hong-min Ren, Jin-lian Zhang

**Affiliations:** School of Pharmacy, Jiangxi University of Traditional Chinese Medicine, Nanchang, 330004 China

**Keywords:** *Paeoniae Radix* Alba, Network pharmacology, Iron-deficiency anaemia

## Abstract

**Background:**

*Paeoniae Radix* Alba, the root of the plant *Paeonia lactiflora* Pall, is a common blood-enriching drug in traditional Chinese medicine. Its effectiveness in the clinical treatment of anaemia is remarkable, but its potential pharmacologic mechanism has not been clarified.

**Methods:**

In this study, the potential pharmacologic mechanism of *Paeoniae Radix* Alba in the treatment of iron-deficiency anaemia was preliminarily elucidated through systematic and comprehensive network pharmacology.

**Results:**

Specifically, we obtained 15 candidate active ingredients from among 146 chemical components in *Paeoniae Radix* Alba. The ingredients were predicted to target 77 genes associated with iron-deficiency anaemia. In-depth analyses of these targets revealed that they were mostly associated with energy metabolism, cell proliferation, and stress responses, suggesting that *Paeoniae Radix* Alba helps alleviate iron-deficiency anaemia by affecting these processes. In addition, we conducted a core target analysis and a cluster analysis of protein-protein interaction (PPI) networks. The results showed that four pathways, the p53 signalling pathway, the IL-17 signalling pathway, the TNF signalling pathway and the AGE-RAGE signalling pathway in diabetic complications, may be major pathways associated with the ameliorative effects of *Paeoniae Radix* Alba on iron-deficiency anaemia. Moreover, molecular docking verified the credibility of the network for molecular target prediction.

**Conclusions:**

Overall, this study predicted the functional ingredients in *Paeoniae Radix* Alba and their targets and uncovered the mechanism of action of this drug, providing new insights for advanced research on *Paeoniae Radix* Alba and other traditional Chinese medicines.

## Background

Worldwide, 46% of children aged 5 to 14 years and 48% of pregnant women suffer from iron-deficiency anaemia (IDA) [1, 2]. IDA is one of the most widespread nutritional deficiency diseases [3] and can cause cognitive deficiency and irreversible auditory and visual system damage in infants [4, 5]. Pregnant women with anaemia may give birth to infants with foetal dysplasia and low birth weight [6, 7]. Iron supplements are widely used to treat IDA [8]. However, long-term use of supplements containing ferrous salts can cause side effects such as epigastric pain, diarrhoea and constipation [9, 10]. Thus, identification of a good alternative supplement with fewer side effects has become an important research objective.

In China, traditional Chinese medicine (TCM) is not simply a cultural practice, it also has a history of thousands of years of use for the treatment of various diseases. Under the guidance of the overall concepts and principles of syndrome differentiation and treatment, TCM has achieved satisfactory clinical results for anaemia treatment. *Paeoniae Radix* Alba (PRA), the root of the plant *Paeonia lactiflora* Pall (family Ranunculaceae), is a TCM with the functions of nourishing blood, astringing Yin, preventing perspiration, regulating menstruation, extinguishing liver wind and relieving pain [11, 12].

In recent years, an unconventional novel analytical technique called network pharmacology has been widely used in TCM research [13–16]. Combined with extensive data analysis, network pharmacology can systematically determine the effects and mechanisms of drugs employed to treat complex diseases at the molecular, cellular, tissue, and biological levels [17]. Although PRA is noteworthy in treating anaemia, for IDA, the active compositions, drug targets, and exact molecular mechanism are still unclear [12, 18–20].

**Fig. 1:**
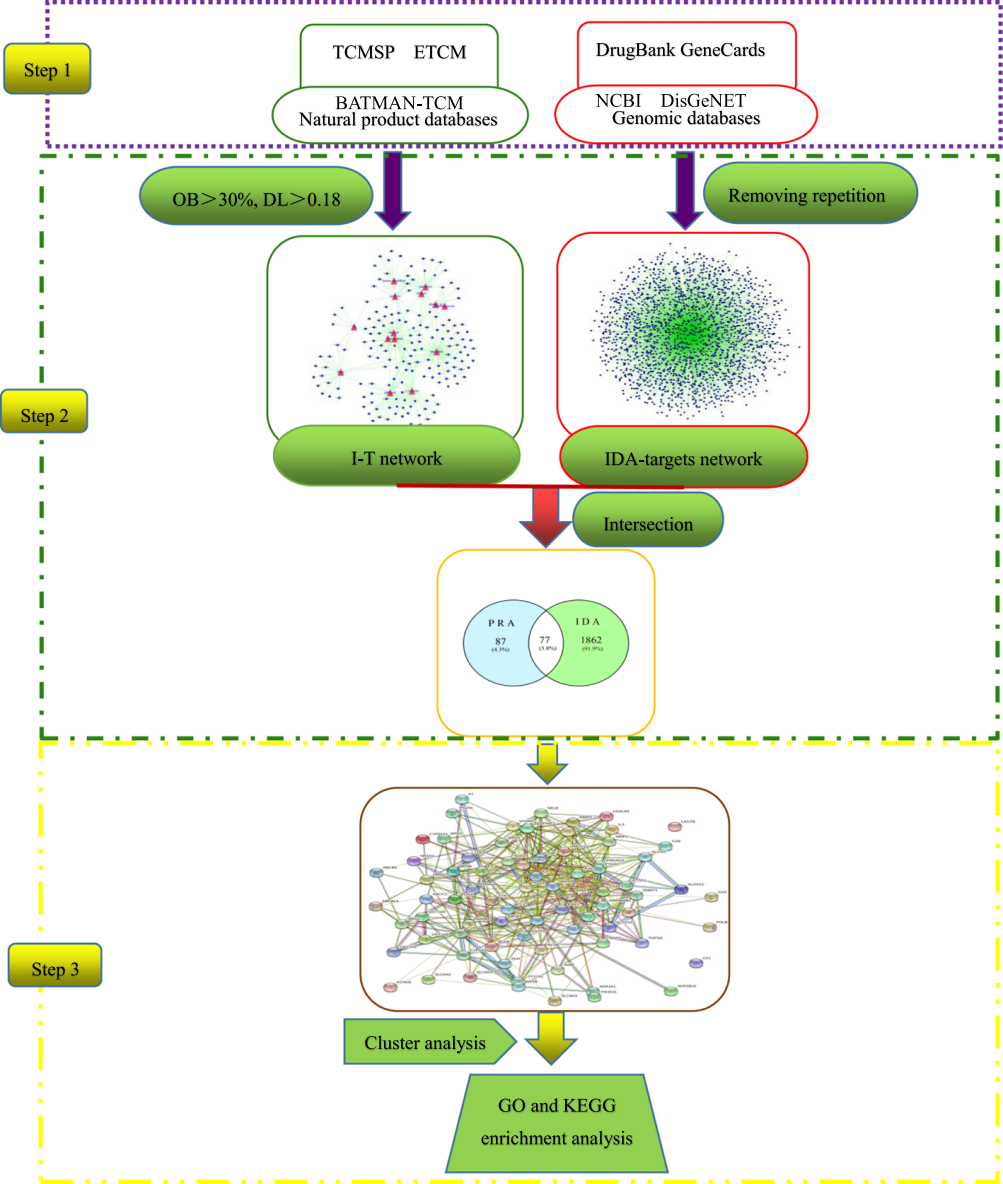
Diagram of the study design. Step 1: Gathered the chemical composition of PRA from three databases (TCMSP, ETCM, BATMAN-TCM), and collected IDA-related targets from four databases (DrugBank, GeneCards, NCBI, DisGeNET). Step 2: Select the candidate components and take the intersection of the component-target and the IDA-target. Step 3: KEGG and GO enrichment analysis

In this study, network pharmacology was utilized to analyse the active ingredients, drug targets and key pathways of PRA in the treatment of IDA, as shown in Fig. 1. This study provides a new perspective for studying the mechanisms of TCMs.

## Methods

### Data sources

#### Ingredients of PRA

Information on the chemical composition of PRA was gathered from three databases: the Traditional Chinese Medicine Systems Pharmacology Database and Analysis Platform (TCMSP, a unique pharmacologic platform for Chinese herbal medicine that can be used to search for the relationships among drugs, targets and diseases), the Encyclopedia of Traditional Chinese Medicine (ETCM, a database of commonly used herbs and herbal formulations that includes standardized information and ingredient information), and the Bioinformatics Analysis Tool for Molecular mechANisms of TCM (BATMAN-TCM, a biological online networking tool that provides users with basic information about herbs, such as their ingredients, targets, and disease relationships) [21–23]. In this experiment, “baishao” was invoked as the keyword, and the structures of the components were saved in MOL2 format. The structures of the components were verified with ChemSpider and SciFinder.

#### IDA-related targets

Targets of IDA were identified with four databases: GeneCards (a searchable, free, and comprehensive database that provides users with ample information for annotating and predicting human genes), DrugBank (a comprehensive, and freely accessible online database that includes information on drugs and drug targets), DisGeNET (a platform to explore the relationships between genes and diseases), and the NCBI database (National Center for Biotechnology Information, a platform integrating the PubMed, Bookshelf, Blast, Genome and other databases; users can search different targets through different databases of the platform) [24–26]. In this study, we used “iron-deficiency anaemia” as a keyword, searched the “gene” database type, and limited the species to “*Homo sapiens*” to identify IDA-related genes.

### Data preprocessing

#### Screening of active ingredients of PRA

Screening of dynamic components can be conducted on the basis of the five rules of Lipinski: a molecular weight (MW) < 500, a hydrogen acceptor number ≤ 10, a hydrogen donor (HDon) number ≤ 5, a log *P* value of − 2 ~ 5, and a rotatable hydrogen bond number (RBN) ≤ 10. If a compound does not violate two or more of the above principles, it can be considered that the compound has soothing properties [27]. Functional components can also be screened according to oral bioavailability (OB; > 30%) and drug likeness (DL; > 0.18) [28]. The OB value is an important indicator for evaluation of the internal conversion of drugs [29]; a higher OB value of a drug is associated with a higher utilization rate of the drug after oral administration and a greater possibility of clinical application [30, 31]. In this experiment, an OB > 30% and a DL > 0.18 were utilized as the criteria for screening of functional components. A literature review was used to supplement the information on the active components.

#### Target prediction for the bioactive ingredients of PRA

At present, the methods and techniques of drug target prediction can be divided into four parts according to their principles: (1) ligand prediction based on chemical structure similarity and pharmacophore models; (2) machine intelligence learning and prediction, for which standardized names and clear molecular target correspondence are required; (3) molecular docking, in which receptors are used to make predictions; and (4) combined prediction [32]. Building on the current conditions and constraints, we selected ligand prediction as the main method and supplemented it with data from DrugBank.

First, the TCMSP, BATMAN-TCM, and ETCM were chosen as databases for the chemical components of PRA. The active components were screened with the criteria of an OB > 30% and a DL > 0.18, and the component targets were then rigorously predicted by SwissTargetPrediction (which compares the components to a library containing 28,000 compounds by two-dimensional and three-dimensional similarity and further predicts any applicable molecular targets from among more than 2000 targets in five different organisms) and Stitch (which can randomly select at least four predicted linked proteins based on a single protein name, multiple protein names, or amino acid sequences with moderate or better confidence) [33]. In addition, listed or laboratory-verified targets in DrugBank were identified as supplementary data. In this experiment, we converted the dynamic component into the “SMILES” format and selected *Homo sapiens* as the species. To ensure the accuracy of the results, we used *P* > 0.5 as the constraint condition for the predicted targets, yielding moderate credibility. After that, we obtained the drug active ingredient targets.

Second, to consolidate and standardize the data, we identified the gene names of the predicted target proteins with Universal Protein (UniProt), a comprehensive resource of protein sequences and annotation data. UniProt is a compilation of the UniProt Knowledgebase, the UniProt Reference Cluster, and the UniProt Archive [34]. We restricted the species to humans and created a protein-gene document.

Finally, to identify the universal targets between IDA and PRA, we uploaded the two target networks to the Venny 2.1 online server and obtained 77 common targets (Table 2) [35]. We then used the WEB-based Gene SeT AnaLysis Toolkit (WebGestalt) online tool to carry out Gene Ontology (GO) analysis on the shared targets for the Biological Process (BP), Cellular Component (CC), Molecular Function (MF) GO categories [36]. Enriched terms in the BP, CC, and MF categories were selected for display. Additionally, we utilized ClueGo in Cytoscape (v 3.6.1) for pathway analysis using data from the Kyoto Encyclopedia of Genes and Genomes (KEGG).

### Protein-protein interaction (PPI) network construction for IDA and PRA

Physiological processes are not only affected by single signals; rather, the expression and function of a gene/protein are often impacted by multiple genes [37]. PPI networks are interaction networks between targets and proteins [38]. We uploaded the obtained targets to the tool of Search Tool for the Retrieval of Interacting Genes/Proteins (STRING) Version 11.0 to develop a PPI network. STRING calculates comprehensive scores and probabilities based on distinct lines of evidence and corrects for the probabilities of random interactions. A minimum score of 0.4 indicates moderate credibility, a minimum score of 0.7 indicates high credibility, and a minimum score of 0.9 indicates the highest credibility [39]. In this study, we constructed a PPI network with a score of 0.4; thus, it was a moderate-credibility network.

### Cluster analyses for the PPI network

Clustering refers to the identification of highly correlated groups of different compounds or objects with similar basic attributes [40]. Cluster analysis, an important classification method, indicates the effectiveness of the classification used for the PPI network. Many algorithms for Cytoscape PPI network clustering analysis have been reported, but previous studies have shown that the molecular complex detection (MCODE) algorithm for protein complex detection is the most reliable for gene network module analysis [41, 42]; thus, we choose MCODE for our PPI network cluster analysis.

### GO function and KEGG pathway enrichment analyses

To determine the commonality among targets, the GO and KEGG pathways of clustered targets are commonly analysed [42]. WebGestalt is a feature-rich web analytics tool; as of 14 January 2019, it covers 354 databases that support 12 organisms and 321,251 functional classifications. It also enables analysis of genes that are not in the database and of data from experimental organisms on the platform [36]. In this study, we examined the enriched GO functions for each target classification and conducted KEGG pathway analysis of the targets with WebGestalt.

### Drug-ingredient-target-pathway-disease (D-I-T-P-D) network construction

The network obtained from the above experiment was introduced into Cytoscape (v 3.6.1), and the “merge” tool was utilized to merge the network. The D-I-T-P-D network was obtained.

### Molecular docking verification

The LibDock module of Discovery Studio 2016 was used to verify the molecular docking based on the functional components of PRA, and a heat map was constructed from the component-core target docking scores.

## Results

### Component-target networks of PRA

We collected data on the chemical components of PRA from three databases, namely, the TCMSP, ETCM, and BATMAN-TCM. The numbers of chemical components derived from these three databases were 85, 59, and 35, respectively. After removing duplicates, we obtained 146 chemical components. Through screening of OB values and DL values, 13 qualified chemical components were obtained (Table 1). The literature shows that albiflorin and paeoniflorin are the active ingredients of PRA responsible for its ameliorative effects on anaemia [20], and gallic acid has anti-inflammatory, antioxidant and antitumour effects [43]. Therefore, these chemical components were also considered candidate components, and their structures were verified with SciFinder and ChemSpider.

**Table 1:** Candidate active ingredients of PRA

NO.	ID	Name	OB%	DL
1	MOL001910	11alpha,12alpha-epoxy-3beta-23-dihydroxy-30-norolean-20-en-28,12beta-olide	64.77	0.38
2	MOL001919	(3S,5R,8R,9R,10S,14S)-3,17-dihydroxy-4,4,8,10,14-pentamethyl-2,3,5,6,7,9-hexahydro-1H-cyclopenta [a]phenanthrene-15,16-dione	43.56	0.53
3	MOL001918	paeoniflorigenone	87.59	0.37
4	MOL001921	Lactiflorin	49.12	0.8
5	MOL001924	paeoniflorin	53.87	0.79
6	MOL001925	paeoniflorin_qt	68.18	0.4
7	MOL001928	albiflorin_qt	66.64	0.33
8	MOL001930	benzoylpaeoniflorin	31.27	0.75
9	MOL000211	Mairin	55.38	0.78
10	MOL000358	beta-sitosterol	36.91	0.75
11	MOL000359	sitosterol	36.91	0.75
12	MOL000422	kaempferol	41.88	0.24
13	MOL000492	(+)-catechin	54.83	0.24
14	MOL001927	Albiflorin	12.09	0.77
15	MOL000513	gallic acid	31.69	0.04

To more intuitively indicate the relationships between components and targets, we constructed a component-target network diagram with Cytoscape (v 3.6.1) [44] that contained 178 nodes and 264 edges. In this network diagram, we found that the median degree of connectivity among 12 components was greater than 6; specifically, kaempferol, beta-sitosterol, (+)-catechin and gallic acid exhibited 61, 38, 32 and 32 degrees of connectivity, respectively, indicating that these four components are important active ingredients in PRA.

### Target networks associated with IDA

**Fig. 2:**
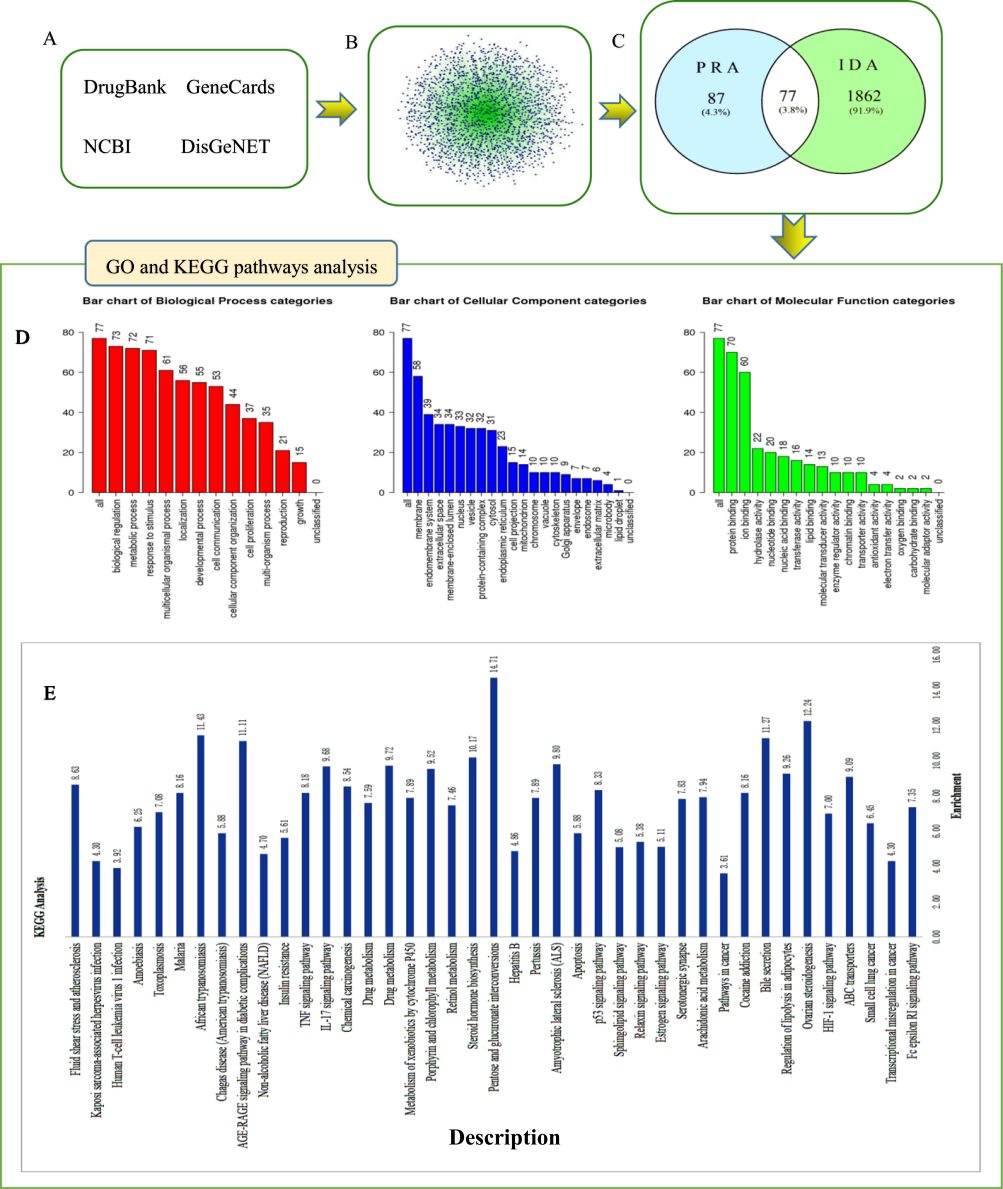
IDA-related target network. **a** Four disease-related gene target databases. **b** IDA target network containing 1940 nodes and 1939 edges. **c** Seventy-seven common targets between IDA and PRA. **d** GO and **e** KEGG pathway enrichment analysis results for PRA-targeted genes associated with IDA

The development of a disease is usually associated with multiple genes or proteins, as is the case for IDA. In this study, we identified 1923, 60, 25 and 29 IDA-related genes from the GeneCards, DisGeNET, NCBI and DrugBank databases, respectively. Duplicates were removed, and 1939 related genes were obtained. A total of 77 genes were shared between PRA targets and IDA-related genes (Table 2). To investigate the relationships between the 77 common targets and IDA, we conducted GO and KEGG analyses of the shared targets (Fig. 2). Ultimately, we obtained 12 enriched BP terms, 19 enriched CC terms, 16 enriched MF terms and 40 enriched KEGG pathways. The BP category results mainly indicated enrichment for the biological regulation (73/77), metabolic process (72/77), response to stimulus (71/77), multicellular organismal process (76/77), localization (56/77), developmental process (55/77), and cell communication (53/77) terms. The membrane (58/77), endomembrane system (39/77) and membrane-enclosed lumen (34/77) terms were significantly enriched in the CC category. The protein binding (70/77) and ion binding (60/77) terms were the primary enriched MF terms identified in our study. In addition, nitrogen metabolism was the most significantly enriched pathway. This suggests that nitrogen metabolism may be the core process affecting IDA. IDA development has been found to play roles in a variety of diseases, such as hepatitis B, amoebiasis, toxoplasmosis, malaria, African trypanosomiasis, and prostate cancer, suggesting that IDA may be affected by one or more diseases. The NF-kappa B signalling pathway, the HIF-1 signalling pathway, the AGE-RAGE signalling pathway in diabetic complications, the pentose and glucuronate interconversion pathways, and the IL-17 signalling pathway were also identified in this study. The results show that the TCM PRA affects multiple pathways and processes in the context of IDA treatment.

**Table 2:** Common targets between PRA and IDA

NO.	Protein ID	Gene name	Protein name	Protein Class
1	P31645	SLC6A4	solute carrier family 6 member 4	transporter
2	Q01959	SLC6A3	solute carrier family 6 member 3	transporter
3	P23975	SLC6A2	solute carrier family 6 member 2	transporter
4	P33527	ABCC1	ATP binding cassette subfamily C member 1	transporter
5	P19793	RXRA	retinoid X receptor alpha	nucleic acid binding; receptor; transcription factor
6	P03372	ESR1	estrogen receptor 1	nucleic acid binding; receptor; transcription factor
7	P05412	JUN	Jun proto-oncogene, AP-1 transcription factor subunit	nucleic acid binding; transcription factor
8	P10275	AR	androgen receptor	nucleic acid binding; receptor; transcription factor
9	P37231	PPARG	peroxisome proliferator activated receptor gamma	nucleic acid binding; receptor; transcription factor
10	P27338	MAOB	monoamine oxidase B	nucleic acid binding; oxidoreductase; transferase
11	P55055	NR1H2	nuclear receptor subfamily 1 group H member 2	nucleic acid binding; receptor;transcription factor
12	O75469	NR1I2	nuclear receptor subfamily 1 group I member 2	nucleic acid binding; receptor; transcription factor
13	P06746	POLB	DNA polymerase beta	nucleic acid binding
14	O95342	ABCB11	ATP binding cassette subfamily B member 11	hydrolase; protease
15	Q2M3G0	ABCB5	ATP binding cassettesubfamily B member 5	hydrolase; protease
16	P22303	ACHE	Acetylcholinesterase	hydrolase; protease
17	P03956	MMP1	matrix metallopeptidase 1	hydrolase; protease
18	P00734	F2	coagulation factor II, thrombin	hydrolase; protease
19	P08253	MMP2	matrix metallopeptidase 2	hydrolase; protease
20	P08183	ABCB1	ATP binding cassette subfamily B member 1	hydrolase; protease
21	P08709	F7	coagulation factor VII	hydrolase; protease
22	P45983	MAPK8	mitogen-activated protein kinase 8	kinase; transferase
23	Q13315	ATM	ATM serine/threonine kinase	kinase;nucleic acid binding; transferase
24	P06493	CDK1	cyclin dependent kinase 1	kinase; transferase
25	P48736	PIK3CG	phosphatidylinositol-4,5-bisphosphate 3-kinase catalytic subunit gamma	kinase; transferase
26	P47989	XDH	xanthine dehydrogenase	oxidoreductase
27	P04040	CAT	catalase	oxidoreductase
28	P09917	ALOX5	arachidonate 5-lipoxygenase	oxidoreductase
29	P08684	CYP3A4	cytochrome P450 family 3 subfamily A member 4	oxidoreductase
30	P16050	ALOX15	arachidonate 15-lipoxygenase	oxidoreductase
31	P23219	PTGS1	prostaglandin-endoperoxide synthase 1	oxidoreductase
32	P05177	CYP1A2	cytochrome P450 family 1 subfamily A member 2	oxidoreductase
33	P35354	PTGS2	prostaglandin-endoperoxide synthase 2	oxidoreductase
34	Q16850	CYP51A1	cytochrome P450 family 51 subfamily A member 1	oxidoreductase
35	P09601	HMOX1	heme oxygenase 1	oxidoreductase
36	P18054	ALOX12	arachidonate 12-lipoxygenase, 12S type	oxidoreductase
37	P14550	AKR1A1	aldo-keto reductase family 1 member A1	oxidoreductase
38	O60218	AKR1B10	aldo-keto reductase family 1 member B10	oxidoreductase
39	P04141	CSF2	colony stimulating factor 2	signaling molecule
40	P10415	BCL2	BCL2, apoptosis regulator	signaling molecule
41	P01375	TNF	tumor necrosis facto	signaling molecule
42	P27487	DPP4	dipeptidyl peptidase 4	enzyme modulator; hydrolase; protease
43	P05121	SERPINE1	serpin family E member 1	enzyme modulator
44	P42574	CASP3	caspase 3	enzyme modulator; hydrolase; protease
45	P07550	ADRB2	adrenoceptor beta 2	receptor
46	P25105	PTAFR	platelet activating factor receptor	receptor
47	P08238	HSP90AB1	heat shock protein 90 alpha family class B member 1	chaperone
48	P04637	TP53	tumor protein p53	transcription factor
49	P17931	LGALS3	galectin 3	cell adhesion molecule; signaling molecule
50	P05231	IL6	interleukin 6	None
51	P00918	CA2	carbonic anhydrase 2	None
52	P00915	CA1	carbonic anhydrase 1	None
53	P01130	LDLR	low density lipoprotein receptor	None
54	P11388	TOP2A	DNA topoisomerase II alpha	None
55	P35503	UGT1A3	UDP glucuronosyltransferase family 1 member A3	None
56	O60656	UGT1A9	UDP glucuronosyltransferase family 1 member A9	None
57	P04035	HMGCR	3-hydroxy-3-methylglutaryl -CoA reductase	None
58	P10636	MAPT	microtubule associated protein tau	None
59	P26358	DNMT1	DNA methyltransferase 1	None
60	P35228	NOS2	nitric oxide synthase 2	None
61	P05093	CYP17A1	Cytochrome P450 family 17 subfamily A member 1	None
62	P16581	SELE	selectin E	None
63	P04114	APOB	apolipoprotein B	None
64	P17612	PRKACA	protein kinase cAMP-activated catalytic subunit alpha	None
65	O00255	MEN1	menin 1	None
66	P08700	IL3	interleukin 3	None
67	P19320	VCAM1	vascular cell adhesion molecule 1	None
68	Q9HAW9	UGT1A8	UDP glucuronosyl transferase family 1member A8	None
69	P29474	NOS3	nitric oxide synthase 3	None
70	Q16790	CA9	carbonic anhydrase 9	None
71	P83111	LACTB	lactamase beta	None
72	P06276	BCHE	butyrylcholinesterase	None
73	P09211	GSTP1	glutathione S-transferase pi 1	None
74	P06213	INSR	insulin receptor	None
75	P14679	TYR	tyrosinase	None
76	Q12809	KCNH2	potassium voltage-gated channel subfamily H member 2	None
77	P27169	PON1	paraoxonase 1	None

### PRA-IDA PPI networks

**Fig. 3:**
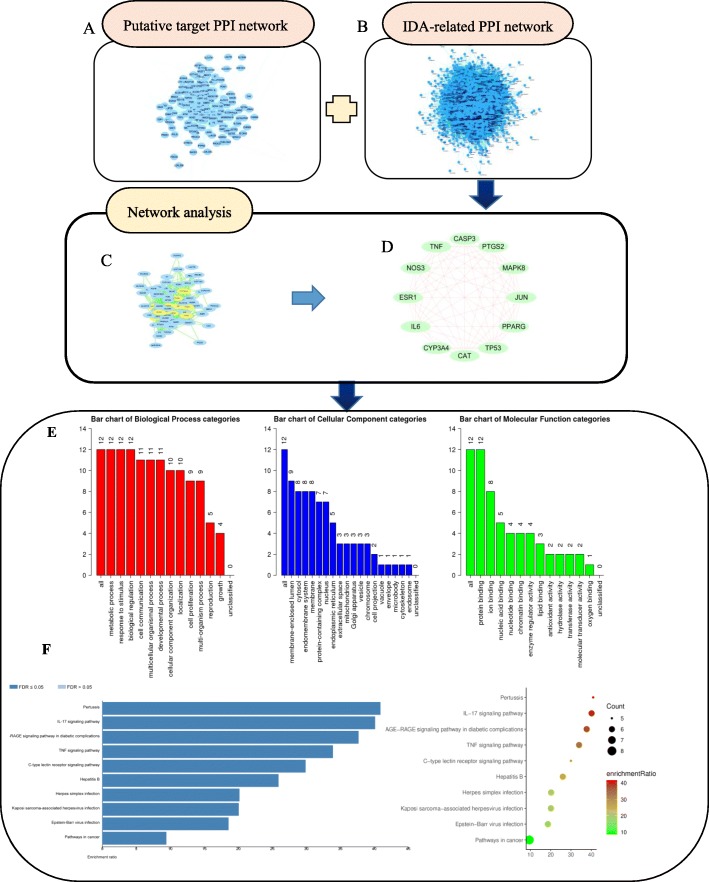
Enrichment analysis of 12 core targets. **a** Putative target PPI network of PRA. **b** IDA-related PPI network. **c** and **d** Analysis network; targets with DC values > 27.789 were considered core targets. **e** GO and **f** KEGG pathway analysis results

To develop a better understanding of the association between PRA and IDA, we analysed the relationships between them through assessment of their core targets. The screening condition of a degree centrality (DC) > 2× the average degree for the core targets yielded 12 strategic targets. The results of the GO function and KEGG pathway enrichment analyses were very similar to the enrichment results for the 77 targets (Fig. 3). The top eight functional terms were the biological regulation (12/12), metabolic process (12/12), response to stimulus (12/12), membrane-enclosed lumen (9/12), cytosol (8/12), endomembrane system (8/12), protein binding (12/12), and ion binding (8/12) terms. These enriched terms were highly correlated with anti-inflammatory activity, especially in the context of chronic or allergic rhinitis.

**Fig. 4:**
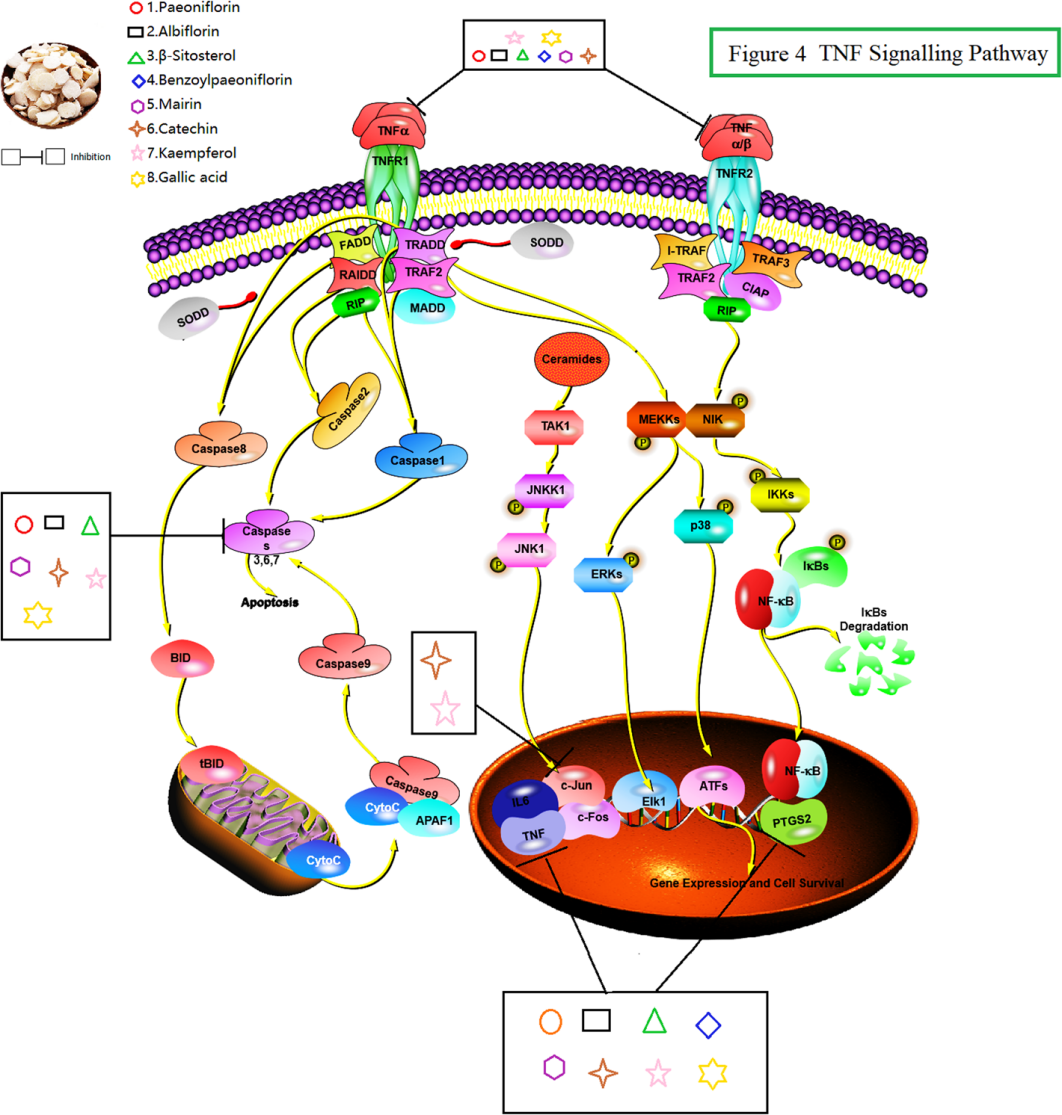
TNF signalling pathway. As shown in the figure, PRA may treat IDA by inhibiting the TNF signalling pathway

The 12 targets were enriched in 10 KEGG pathways with significant false discovery rate (FDR)-adjusted *P*-values, including pertussis and the TNF signalling pathway (Fig. 4). The details of the KEGG pathways are outlined in Additional file 3.

### Enrichment analyses of 75 core targets

**Fig. 5:**
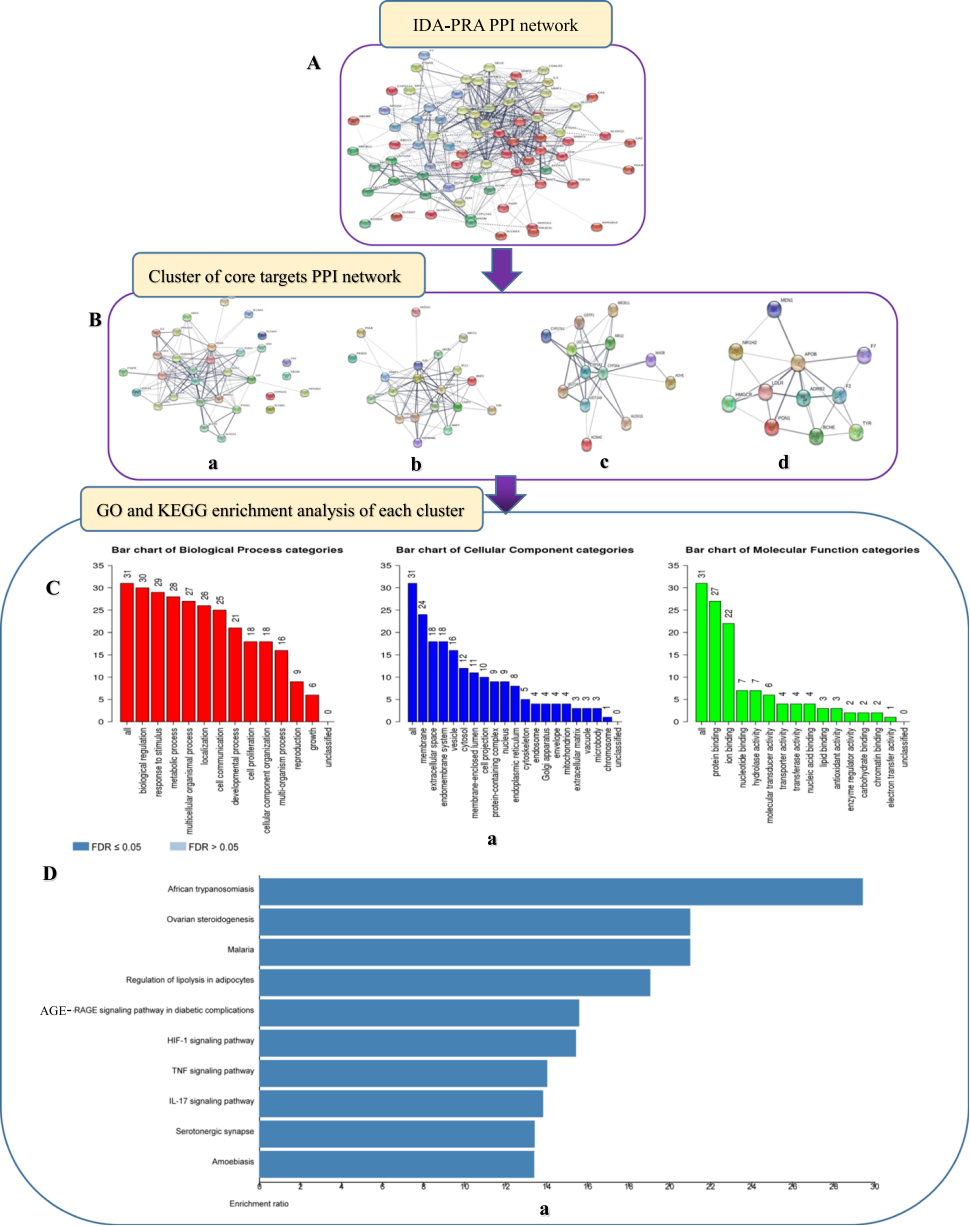
Enrichment analysis of 75 core targets. **A** IDA-PRA PPI network. **B** Clusters of the PPI network. **C** GO and **D** KEGG pathway analysis results for each cluster. Due to space constraints, other Cluster analysis (b, c, d) is shown in file 3

The PPI network comparison of IDA and PRA revealed 75 core targets. To elucidate the biological functions of these targets, we divided the 75 targets into four clusters and subjected them to GO and KEGG pathway analyses (Fig. 5). Based on the GO term results, we found that biological regulation-related processes, such as gene expression, smooth muscle cell proliferation, and nitric oxide biosynthesis; metabolic processes, such as aerobic metabolism and steroid metabolism; responses to stimuli such as hypoxia, oestradiol, and lipopolysaccharide; and other processes, such as enzyme binding, protein homodimerization activity, iron ion binding, RNA polymerase II transcription factor activity, and ligand-activated sequence-specific DNA binding, were enriched for our clusters, suggesting that PRA may help alleviate IDA by affecting enzymes, iron ion binding, stress responses and nitric oxide biosynthesis.

The occurrence and development of diseases can be affected by other diseases and processes. In our study, we found that African trypanosomiasis, malaria, amoebiasis, colorectal cancer, pertussis, hepatitis B, serotonergic synapse-related processes, ovarian steroidogenesis, apoptosis, and cell proliferation could also indirectly affect the development of IDA, suggesting that PRA alleviates IDA by affecting cell-, nerve-, and inflammation-related processes.

In addition, we believe that the top four KEGG pathways identified for these clusters, namely, the HIF-1 signalling pathway, the AGE-RAGE signalling pathway in diabetic complications, the TNF signalling pathway, and the IL-17 signalling pathway, might play significant roles in IDA treatment.

### D-I-T-P-D network construction

**Fig. 6:**
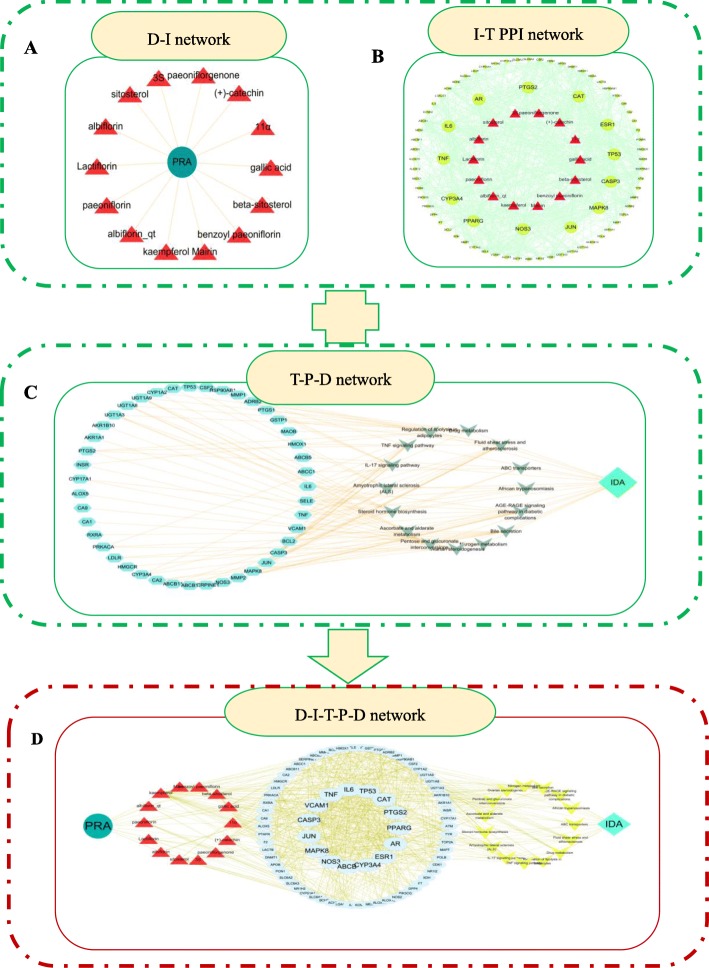
Construction of the D-I-T-P-D network. **a** Ingredient-PRA (ingredient-drug [I-D]) network. **b** Ingredient-target (I-T) network. **c** Target-pathway-disease (T-P-D) network. **d** D-I-T-P-D network containing 108 nodes and 785 edges

On the basis of the PPI targets and pathway analyses, a D-I-T-P-D network was constructed using Cytoscape (v 3.6.1). As illustrated in Fig. 6, this D-I-T-P-D network had 108 nodes and 785 edges. The dark cyan circles, red triangles, celadon ellipses, yellow inverted triangles, and cyan diamond represent PRA, PRA ingredients, target genes, pathways, and IDA, respectively.

### Component-core target docking scores

**Fig. 7:**
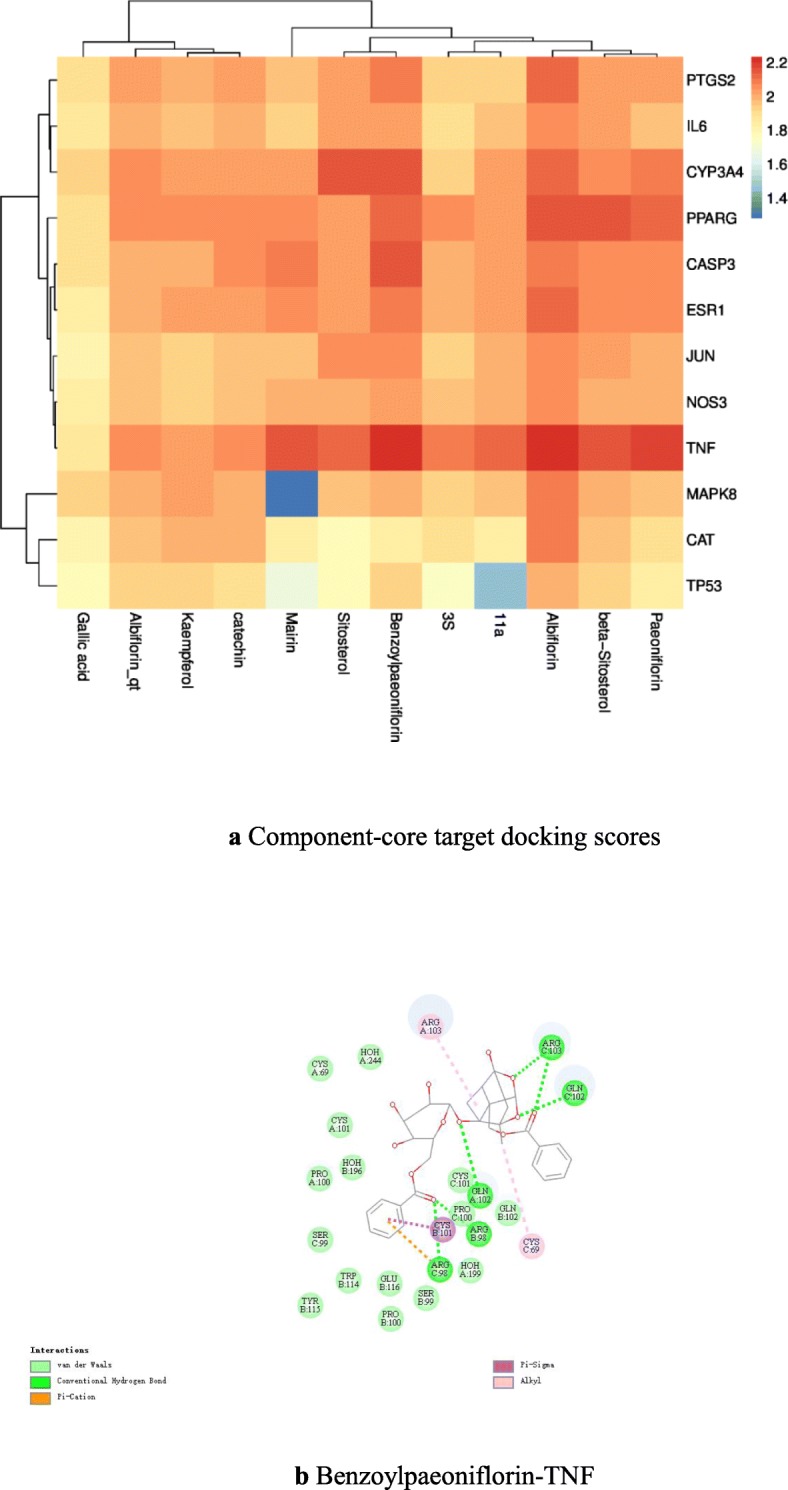
**a** Component-core target docking scores. In this heat map, the docking score between the component and the target is converted by Log10, and the color is closer to red, indicating that the docking score between the component and the target is high and the docking strength is high. **b** benzoylpaeoniflorin-TNF. A two-dimensional diagram of the interaction between benzoylpaeoniflorin and TNF. Most of the oxygen atoms in benzoylpaeoniflorin form hydrogen bonds with ARG98, ARG103, GLN102, and some benzene rings form Pi-Sigma with CYS101

The docking score diagram is given in Fig. 7a. Notably, each active component and core target had good docking ability. PPARG, CYP3A4, and TNF may be the most important targets. Paeoniflorin, benzoylpaeoniflorin, and albiflorin may be the components associated with the ameliorative effects of PRA on IDA, which further confirms the reliability of the network pharmacology. MOL001910 and MOL001919 were replaced by 11α and 3S, respectively. The ligands paeoniflorigenone and lactiflorin could not be constructed, so docking verification was not carried out. Example of molecular docking: benzoylpaeoniflorin-TNF, Fig. 7b.

## Discussion

IDA is a condition that occurs among children in both developing and developed countries, leading to impaired development, activity intolerance, behavioural changes, irritability, and reduced learning ability; women and elderly individuals are also affected [45, 46]. The iron deficiency associated with IDA can be classified as either absolute or functional iron deficiency: absolute iron deficiency is defined as a severe reduction in or loss of iron reserves in the bone marrow, liver, and spleen, while functional iron deficiency is defined as deficiency due to inadequate intake, malabsorption, or metabolic disorders [47–49]. Iron is an important component of human metabolism that plays crucial roles in cellular respiration, DNA synthesis, cell proliferation and oxygen storage [50]. Disorders in iron absorption and metabolism result in severe oxidative stress and tissue damage [51]. PRA is a blood tonic drug that regulates menstruation, but its precise mechanism of action is not yet clear [11]. Therefore, it is imperative to explore the mechanism of PRA in IDA treatment by using network pharmacology methods combined with functional ingredient screening, drug target prediction, and network and pathway analyses.

In this study, we identified 77 common targets between PRA and IDA. These targets were mainly enriched for energy metabolism-, cell proliferation-, and apoptosis-related terms. The main properties of these targets were associated with nucleic acid binding, receptors, and transcription factors (9/77); hydrolases and proteases (8/77); and oxidoreductases (13/77). Many other targets had unknown or other attributes. We reviewed several literature sources and found that most of the components downregulate TNF, IL6, PTGS2, CYP3A4, and CASP3 and upregulate PPARG and CAT, further indicating the reliability of the target prediction, as detailed in Table 3. In addition, we found that IL6 can induce the growth of myeloma and plasma cell tumours and induce the differentiation of nerve cells and that its overexpression in inflammation sites is a main cause of anaemia and chronic inflammation [88, 89]. TP53, which is involved in a variety of cell death pathways and can be used as a marker of neuronal injury, has been widely studied in the context of cancer treatment [90, 91]. TNF and IL6 are involved in the occurrence and development of chronic anaemia/IDA, and the level of TNF expression can reflect the degree of disease of patients with aplastic anaemia [92–94]. IL3 and TNF participate in relieving blood deficiencies in mice caused by cyclophosphamide [19].

**Table 3:** Correlations between components and targets

Components	Downregulate	Upregulate	References
Paeoniflorin	TNF,IL6,PTGS2,CYP3A4,CASP3	PPARG,CAT	[52–58]
Albiflorin	TNF,IL6,PTGS2,CYP3A4,CASP3	NOS3	[52, 54, 57, 59]
β-Sitosterol	IL6,PTGS2,TNF,CYP3A4,CASP3	PPARG,CAT,NOS3	[60–64]
Mairin	PTGS2,TNF,CYP3A4,IL6,CASP3,	PPARG,CAT	[65–69]
(+)-Catechin	TNF,PTGS2,CYP3A4,IL6,CASP3,JUN	PPARG,ESR1,TP53	[70–76]
Kaempferol	PTGS2,CYP3A4,TNF,IL6,CASP3,JUN	PPARG,CAT	[77–82]
Gallic acid	PTGS2,CYP3A4,IL6,TNF,CASP3	PPARG,CAT,NOS3	[61, 83–87]
Benzoylpaeoniflorin	PTGS2,IL6,TNF		[53, 84]

In addition, we compared the KEGG and GO results for the 12 core targets, the 75 targets revealed in the cluster analyses, and the common targets, and the results showed an IDA correlation. We believe that the processes of nitric oxide biosynthesis, arachidonic acid metabolism, and Th17 cell differentiation play important roles in IDA [95–97]. Effects on gene expression, steroid metabolism, hypoxia responses, and protein homodimerization activity may be responsible for a large proportion of the treatment effects.

Subsequently, we searched for the top four KEGG pathways and identified the p53 signalling pathway, the IL-17 signalling pathway, the TNF signalling pathway, and the AGE-RAGE signalling pathway in diabetic complications. We concluded that these pathways are involved in the treatment of anaemia [98–101]. Interestingly, toxoplasmosis, malaria, African trypanosomiasis, hepatitis B and prostate cancer are all associated with anaemia [102–106], demonstrating that the progression of this disease is influenced by other diseases. To some extent, these results also indicate that PRA contains multiple active components and can achieve multiple objectives through multiple pathways. The findings also serve as a basis for investigation into the collaboration among multiple components in follow-up research.

## Conclusions

In general, we elucidated the multi-component, multi-target, and multi-pathways characteristics of PRA in the context of IDA treatment through network pharmacology. Four pathways, the p53 signalling pathway, the IL-17 signalling pathway, the TNF signalling pathway, and the AGE-RAGE signalling pathway in diabetic complications, were identified, providing new directions for the development of drugs for IDA.

## Supplementary information

**Additional file 1: Table 1.** Candidate active ingredients of PRA; **Table 2.** Common targets between PRA and IDA; **Table 3.** Correlations between components and targets.

**Additional file 2: File 2a: Table 1** Known therapeutic targets for IDA; **File 2b: Table 2** Targets for PRA.

**Additional file 3.** File for all pictures.

**Additional file 4.** File for partial targets with components docking.

## Data Availability

Datasets supporting the results of this article have been included in the additional files.

## Abbreviations

IDA: Iron-deficiency anaemia
TCM: Traditional Chinese medicine
PRA: *Paeoniae Radix* Alba
TCMSP: Traditional Chinese Medicine Systems Pharmacology database and analysis platform
PPI: Protein-protein interaction network
ETCM: Encyclopedia of Traditional Chinese Medicine
BATMAN-TCM: Bioinformatics Analysis Tool for Molecular mechANisms of TCM
STRING: Search Tool for the Retrieval of Interacting Genes/Proteins
OB: Oral bioavailability
DL: Drug likeness
UniProt: Universal Protein
NCBI: National Center for Biotechnology Information
DC: Degree centrality
GO: Gene ontology
BP: Biological Process
CC: Cellular Component
MF: Molecular Function
KEEG: Kyoto encyclopedia of genes and genome
MCODE: Molecular complex detection
WebGestalt: WEB-based Gene SeT AnaLysis Toolkit
