# Supplementary material for: Uncovering the mechanism of the effects of Paeoniae Radix Alba on iron-deficiency anaemia through a network pharmacology-based strategy
Source: BMC Complement Med Ther. 2020 Apr 28;20:130. doi: 10.1186/s12906-020-02925-4 (PMC7189569; doi:10.1186/s12906-020-02925-4)
Supplement: Supplementary file 1 — Additional file 1: Table 1. Candidate active ingredients of PRA; Table 2. Common targets between PRA and IDA; Table 3. Correlations between components and targets. [file 12906_2020_2925_MOESM1_ESM.doc]

| **Table 1 Candidate active ingredients of PRA** | | | | |
| --- | --- | --- | --- | --- |
| NO. | ID | Name | OB% | DL |
| 1 | MOL001910 | 11alpha,12alpha-epoxy-3beta-23-  dihydroxy-30-norolean-20-en-28,12beta-olide | 64.77 | 0.38 |
| 2 | MOL001919 | (3S,5R,8R,9R,10S,14S)-3,17-dihydroxy-4,4,8,10,14-pentamethyl-  2,3,5,6,7,9-hexahydro-1H-cyclopenta[a]phenanthrene-15,16-dione | 43.56 | 0.53 |
| 3 | MOL001918 | paeoniflorigenone | 87.59 | 0.37 |
| 4 | MOL001921 | Lactiflorin | 49.12 | 0.8 |
| 5 | MOL001924 | paeoniflorin | 53.87 | 0.79 |
| 6 | MOL001925 | paeoniflorin_qt | 68.18 | 0.4 |
| 7 | MOL001928 | albiflorin_qt | 66.64 | 0.33 |
| 8 | MOL001930 | benzoylpaeoniflorin | 31.27 | 0.75 |
| 9 | MOL000211 | Mairin | 55.38 | 0.78 |
| 10 | MOL000358 | beta-sitosterol | 36.91 | 0.75 |
| 11 | MOL000359 | sitosterol | 36.91 | 0.75 |
| 12 | MOL000422 | kaempferol | 41.88 | 0.24 |
| 13 | MOL000492 | (+)-catechin | 54.83 | 0.24 |
| 14 | MOL001927 | Albiflorin | 12.09 | 0.77 |
| 15 | MOL000513 | gallic acid | 31.69 | 0.04 |

| **Table 2 Common targets between PRA and IDA** | | | | |
| --- | --- | --- | --- | --- |
| NO. | Protein ID | Gene name | Protein name | Protein Class |
| 1 | P31645 | SLC6A4 | solute carrier family 6 member 4 | transporter |
| 2 | Q01959 | SLC6A3 | solute carrier family 6 member 3 | transporter |
| 3 | P23975 | SLC6A2 | solute carrier family 6 member 2 | transporter |
| 4 | P33527 | ABCC1 | ATP binding cassette subfamily  C member 1 | transporter |
| 5 | P19793 | RXRA | retinoid X receptor alpha | nucleic acid binding;  receptor; transcription factor |
| 6 | P03372 | ESR1 | estrogen receptor 1 | nucleic acid binding;  receptor; transcription factor |
| 7 | P05412 | JUN | Jun proto-oncogene,  AP-1 transcription factor subunit | nucleic acid binding;  transcription factor |
| 8 | P10275 | AR | androgen receptor | nucleic acid binding;  receptor; transcription factor |
| 9 | P37231 | PPARG | peroxisome proliferator  activated receptor gamma | nucleic acid binding;  receptor; transcription factor |
| 10 | P27338 | MAOB | monoamine oxidase B | nucleic acid binding;  oxidoreductase; transferase |
| 11 | P55055 | NR1H2 | nuclear receptor subfamily  1 group H member 2 | nucleic acid binding;  receptor;transcription factor |
| 12 | O75469 | NR1I2 | nuclear receptor subfamily  1 group I member 2 | nucleic acid binding;  receptor; transcription factor |
| 13 | P06746 | POLB | DNA polymerase beta | nucleic acid binding |
| 14 | O95342 | ABCB11 | ATP binding cassette  subfamily B member 11 | hydrolase; protease |
| 15 | Q2M3G0 | ABCB5 | ATP binding cassette  subfamily B member 5 | hydrolase; protease |
| 16 | P22303 | ACHE | Acetylcholinesterase | hydrolase; protease |
| 17 | P03956 | MMP1 | matrix metallopeptidase 1 | hydrolase; protease |
| 18 | P00734 | F2 | coagulation factor II, thrombin | hydrolase; protease |
| 19 | P08253 | MMP2 | matrix metallopeptidase 2 | hydrolase; protease |
| 20 | P08183 | ABCB1 | ATP binding cassette  subfamily B member 1 | hydrolase; protease |
| 21 | P08709 | F7 | coagulation factor VII | hydrolase; protease |
| 22 | P45983 | MAPK8 | mitogen-activated protein kinase 8 | kinase; transferase |
| 23 | Q13315 | ATM | ATM serine/threonine kinase | kinase;nucleic acid binding;  transferase |
| 24 | P06493 | CDK1 | cyclin dependent kinase 1 | kinase; transferase |
| 25 | P48736 | PIK3CG | phosphatidylinositol-4,5-bisphosphate 3-kinase catalytic subunit gamma | kinase; transferase |
| 26 | P47989 | XDH | xanthine dehydrogenase | oxidoreductase |
| 27 | P04040 | CAT | catalase | oxidoreductase |
| 28 | P09917 | ALOX5 | arachidonate 5-lipoxygenase | oxidoreductase |
| 29 | P08684 | CYP3A4 | cytochrome P450 family 3  subfamily A member 4 | oxidoreductase |
| 30 | P16050 | ALOX15 | arachidonate 15-lipoxygenase | oxidoreductase |
| 31 | P23219 | PTGS1 | prostaglandin-endoperoxide synthase 1 | oxidoreductase |
| 32 | P05177 | CYP1A2 | cytochrome P450 family 1  subfamily A member 2 | oxidoreductase |
| 33 | P35354 | PTGS2 | prostaglandin-endoperoxide synthase 2 | oxidoreductase |
| 34 | Q16850 | CYP51A1 | cytochrome P450 family 51  subfamily A member 1 | oxidoreductase |
| 35 | P09601 | HMOX1 | heme oxygenase 1 | oxidoreductase |
| 36 | P18054 | ALOX12 | arachidonate 12-lipoxygenase, 12S type | oxidoreductase |
| 37 | P14550 | AKR1A1 | aldo-keto reductase family 1 member A1 | oxidoreductase |
| 38 | O60218 | AKR1B10 | aldo-keto reductase family 1  member B10 | oxidoreductase |
| 39 | P04141 | CSF2 | colony stimulating factor 2 | signaling molecule |
| 40 | P10415 | BCL2 | BCL2, apoptosis regulator | signaling molecule |
| 41 | P01375 | TNF | tumor necrosis facto | signaling molecule |
| 42 | P27487 | DPP4 | dipeptidyl peptidase 4 | enzyme modulator;  hydrolase; protease |
| 43 | P05121 | SERPINE1 | serpin family E member 1 | enzyme modulator |
| 44 | P42574 | CASP3 | caspase 3 | enzyme modulator;  hydrolase; protease |
| 45 | P07550 | ADRB2 | adrenoceptor beta 2 | receptor |
| 46 | P25105 | PTAFR | platelet activating factor receptor | receptor |
| 47 | P08238 | HSP90AB1 | heat shock protein 90 alpha family  class B member 1 | chaperone |
| 48 | P04637 | TP53 | tumor protein p53 | transcription factor |
| 49 | P17931 | LGALS3 | galectin 3 | cell adhesion molecule;  signaling molecule |
| 50 | P05231 | IL6 | interleukin 6 | None |
| 51 | P00918 | CA2 | carbonic anhydrase 2 | None |
| 52 | P00915 | CA1 | carbonic anhydrase 1 | None |
| 53 | P01130 | LDLR | low density lipoprotein receptor | None |
| 54 | P11388 | TOP2A | DNA topoisomerase II alpha | None |
| 55 | P35503 | UGT1A3 | UDP glucuronosyltransferase  family 1 member A3 | None |
| 56 | O60656 | UGT1A9 | UDP glucuronosyltransferase  family 1 member A9 | None |
| 57 | P04035 | HMGCR | 1. hydroxy-3-methylglutaryl   -CoA reductase | None |
| 58 | P10636 | MAPT | microtubule associated protein tau | None |
| 59 | P26358 | DNMT1 | DNA methyltransferase 1 | None |
| 60 | P35228 | NOS2 | nitric oxide synthase 2 | None |
| 61 | P05093 | CYP17A1 | Cytochrome P450 family 17  subfamily A member 1 | None |
| 62 | P16581 | SELE | selectin E | None |
| 63 | P04114 | APOB | apolipoprotein B | None |
| 64 | P17612 | PRKACA | protein kinase cAMP-activated  catalytic subunit alpha | None |
| 65 | O00255 | MEN1 | menin 1 | None |
| 66 | P08700 | IL3 | interleukin 3 | None |
| 67 | P19320 | VCAM1 | vascular cell adhesion molecule 1 | None |
| 68 | Q9HAW9 | UGT1A8 | UDP glucuronosyl transferase  family 1member A8 | None |
| 69 | P29474 | NOS3 | nitric oxide synthase 3 | None |
| 70 | Q16790 | CA9 | carbonic anhydrase 9 | None |
| 71 | P83111 | LACTB | lactamase beta | None |
| 72 | P06276 | BCHE | butyrylcholinesterase | None |
| 73 | P09211 | GSTP1 | glutathione S-transferase pi 1 | None |
| 74 | P06213 | INSR | insulin receptor | None |
| 75 | P14679 | TYR | tyrosinase | None |
| 76 | Q12809 | KCNH2 | potassium voltage-gated channel  subfamily H member 2 | None |
| 77 | P27169 | PON1 | paraoxonase 1 | None |

| **Table 3 Correlations between components and targets** | | | |
| --- | --- | --- | --- |
| Components | Downregulate | Upregulate | References |
| Paeoniflorin | TNF,IL6,PTGS2,CYP3A4,CASP3 | PPARG,CAT | [52,54,59,66,71,81,83] |
| Albiflorin | TNF,IL6,PTGS2,CYP3A4,CASP3 | NOS3 | [52,59,72,81] |
| β-Sitosterol | IL6,PTGS2,TNF,CYP3A4,CASP3 | PPARG,CAT,NOS3 | [53,60,67,73,84] |
| Mairin | PTGS2,TNF,CYP3A4,IL6,CASP3, | PPARG,CAT | [55,63,65,74,85] |
| (+)-Catechin | TNF,PTGS2,CYP3A4,IL6,CASP3,JUN | PPARG,ESR1,TP53 | [56,61,68,75,78,79,87] |
| Kaempferol | PTGS2,CYP3A4,TNF,IL6,CASP3,JUN | PPARG,CAT | [57,62,69,76,80,86] |
| Gallic acid | PTGS2,CYP3A4,IL6,TNF,CASP3 | PPARG,CAT,NOS3 | [58,60,64,70,77,82] |
| Benzoylpaeoniflorin | PTGS2,IL6,TNF |  | [54,64] |
|  |  |  |  |
