# Supplementary material for: Uncovering the mechanism of the effects of Paeoniae Radix Alba on iron-deficiency anaemia through a network pharmacology-based strategy
Source: BMC Complement Med Ther. 2020 Apr 28;20:130. doi: 10.1186/s12906-020-02925-4 (PMC7189569; doi:10.1186/s12906-020-02925-4)

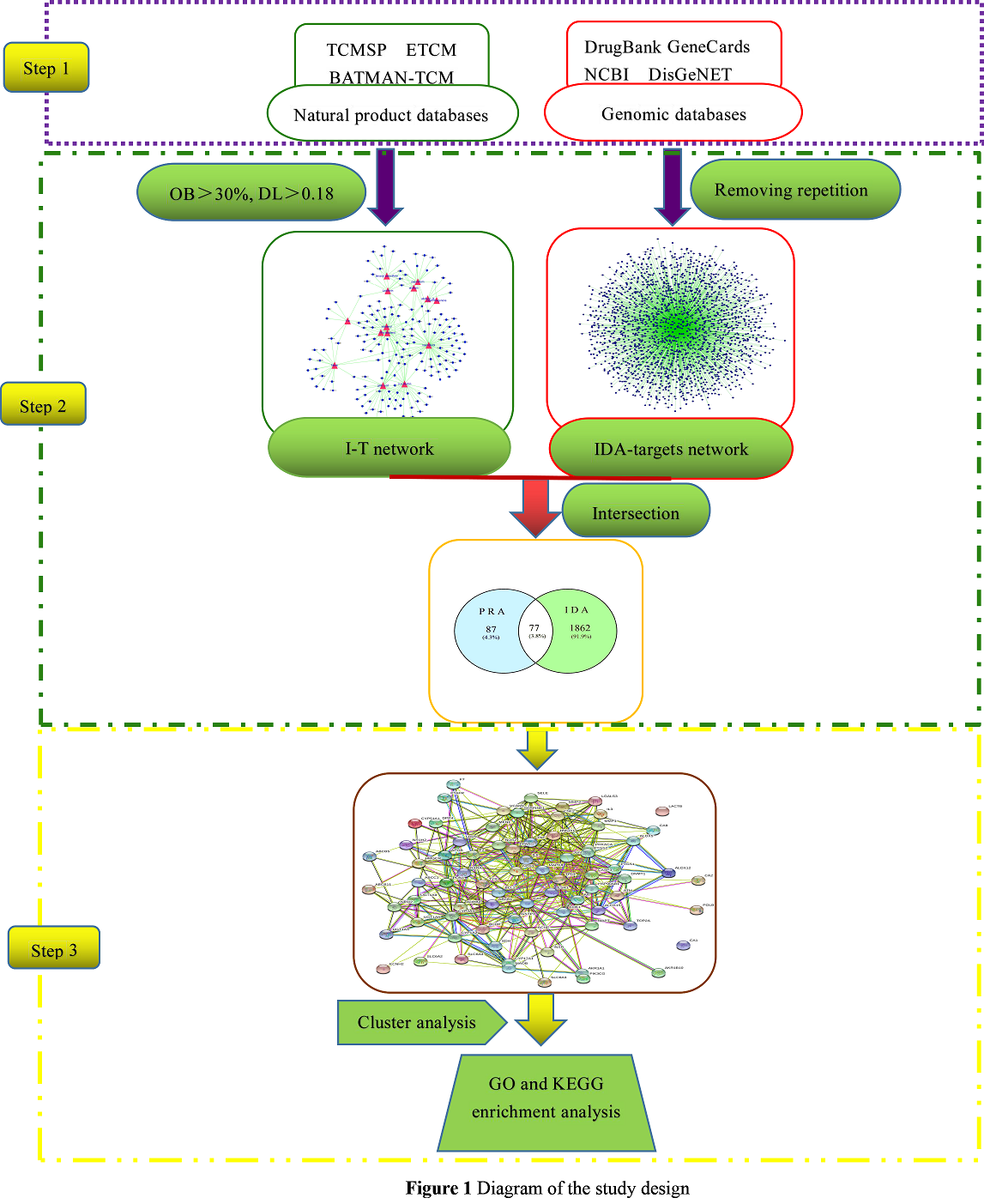

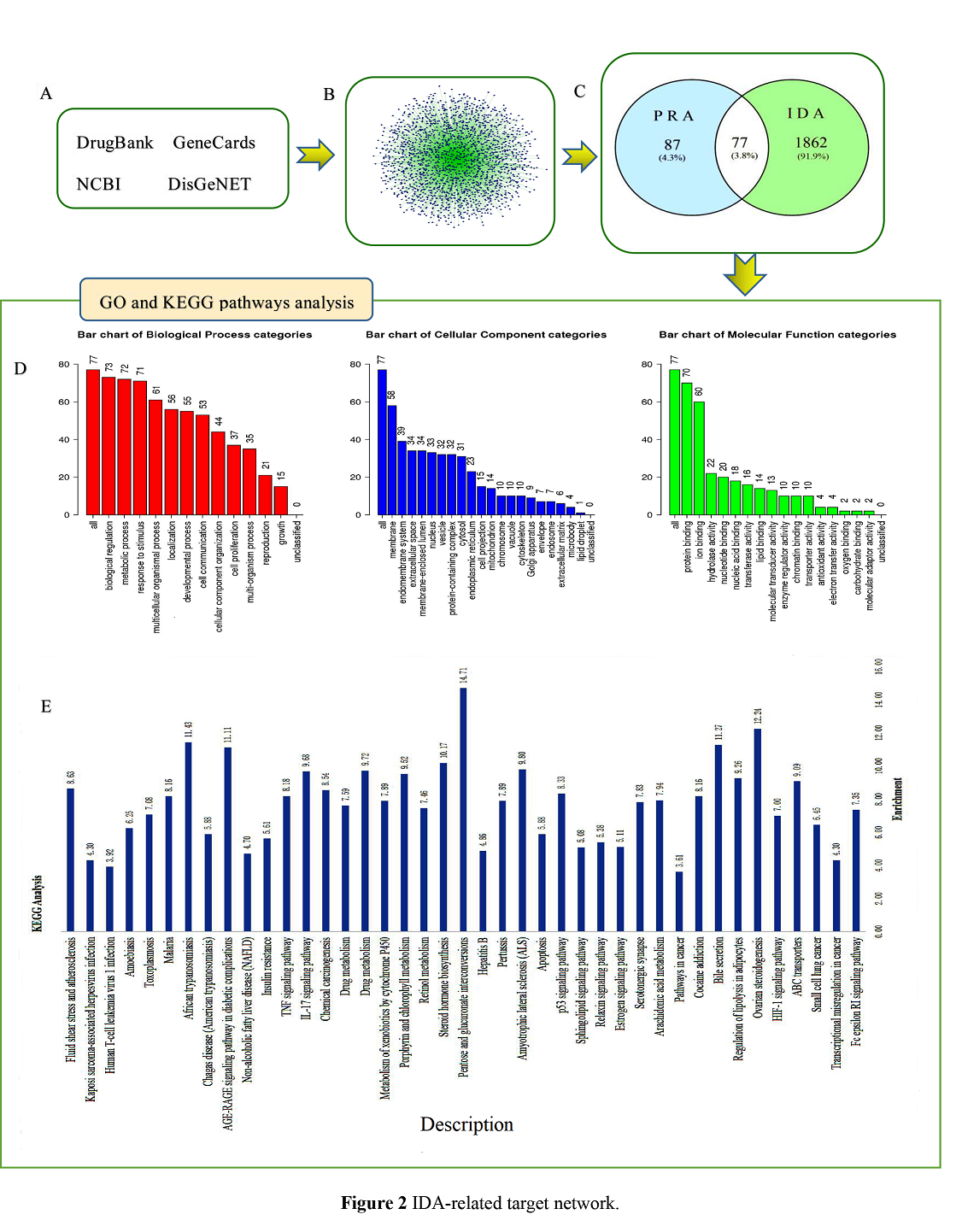

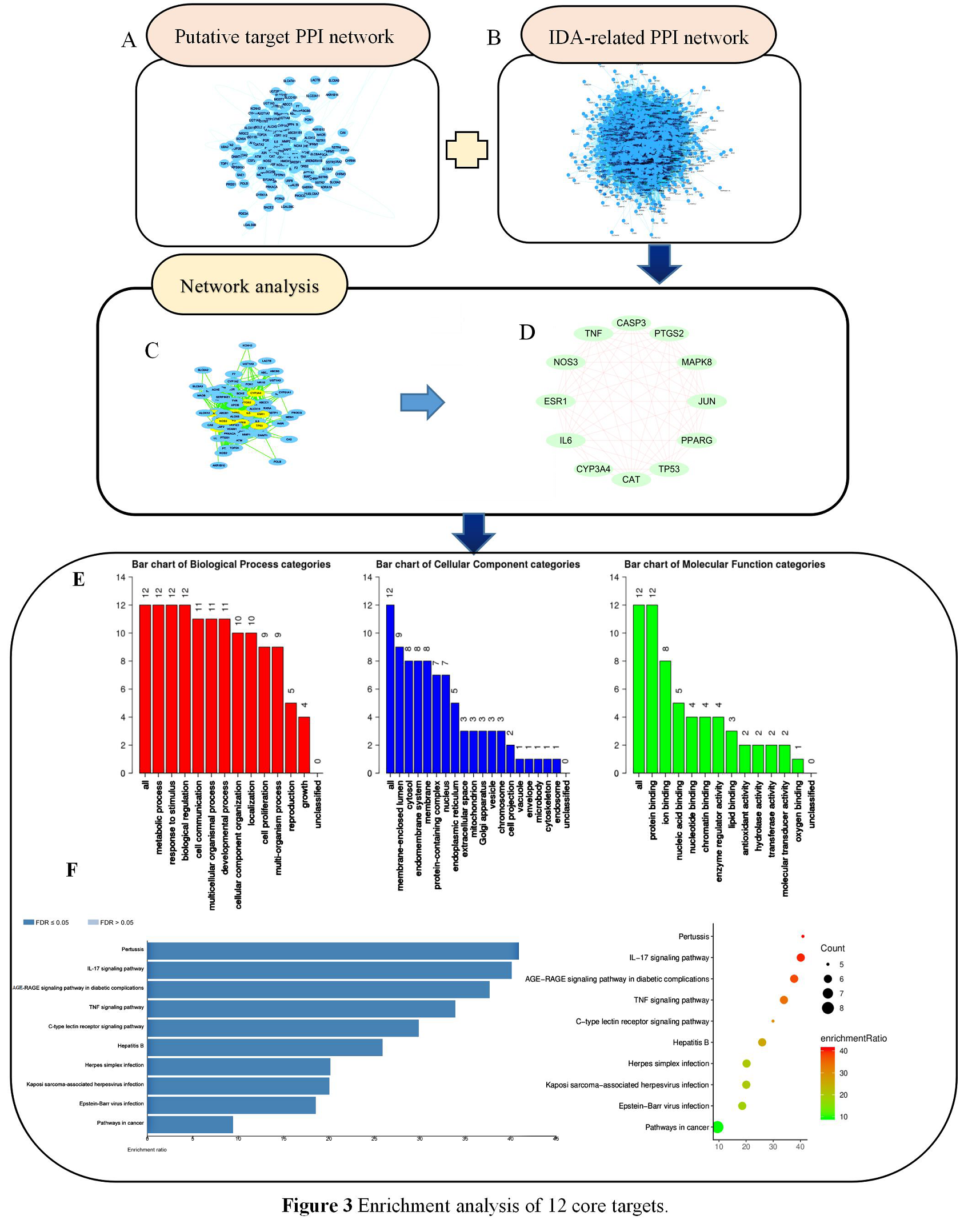

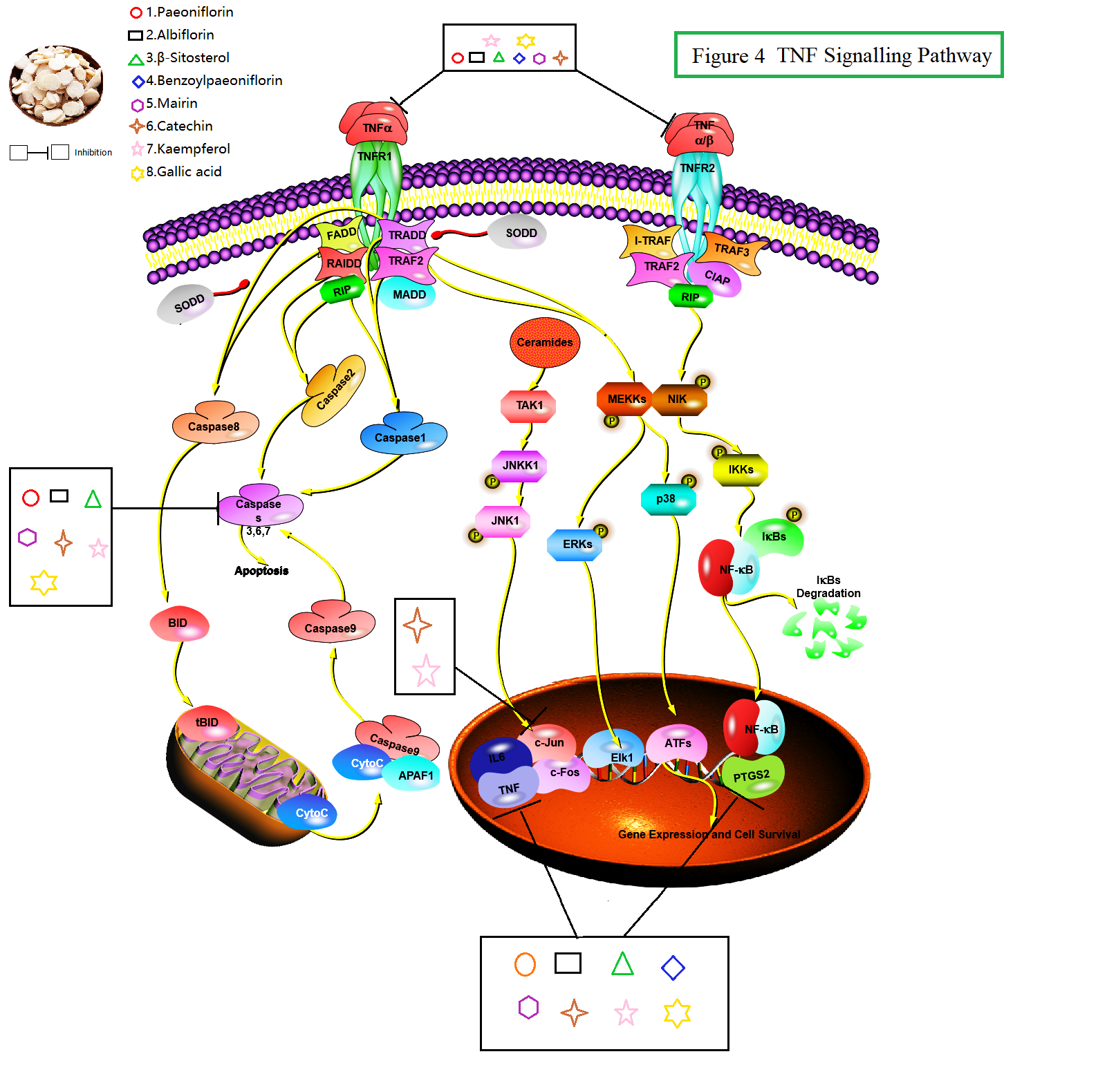

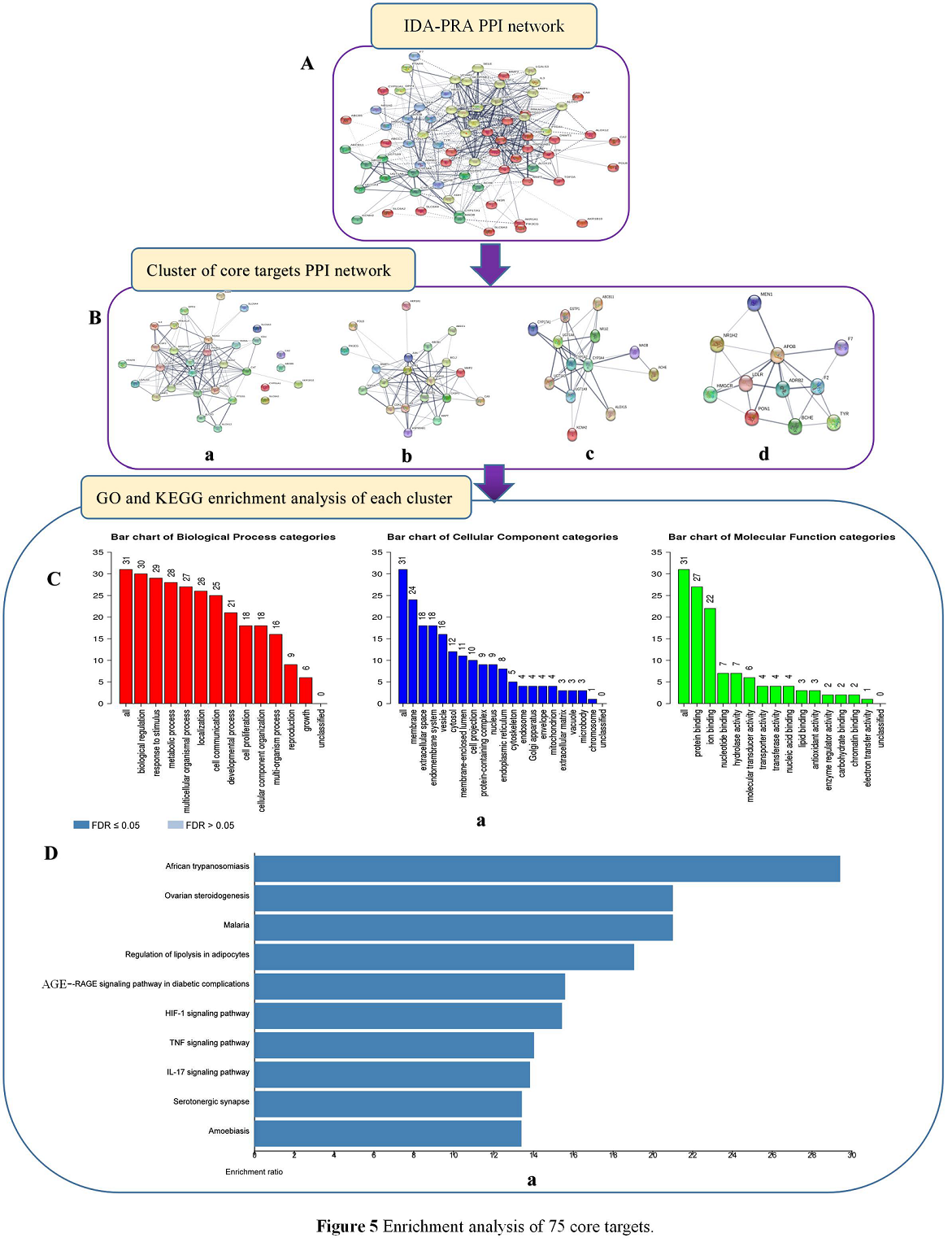

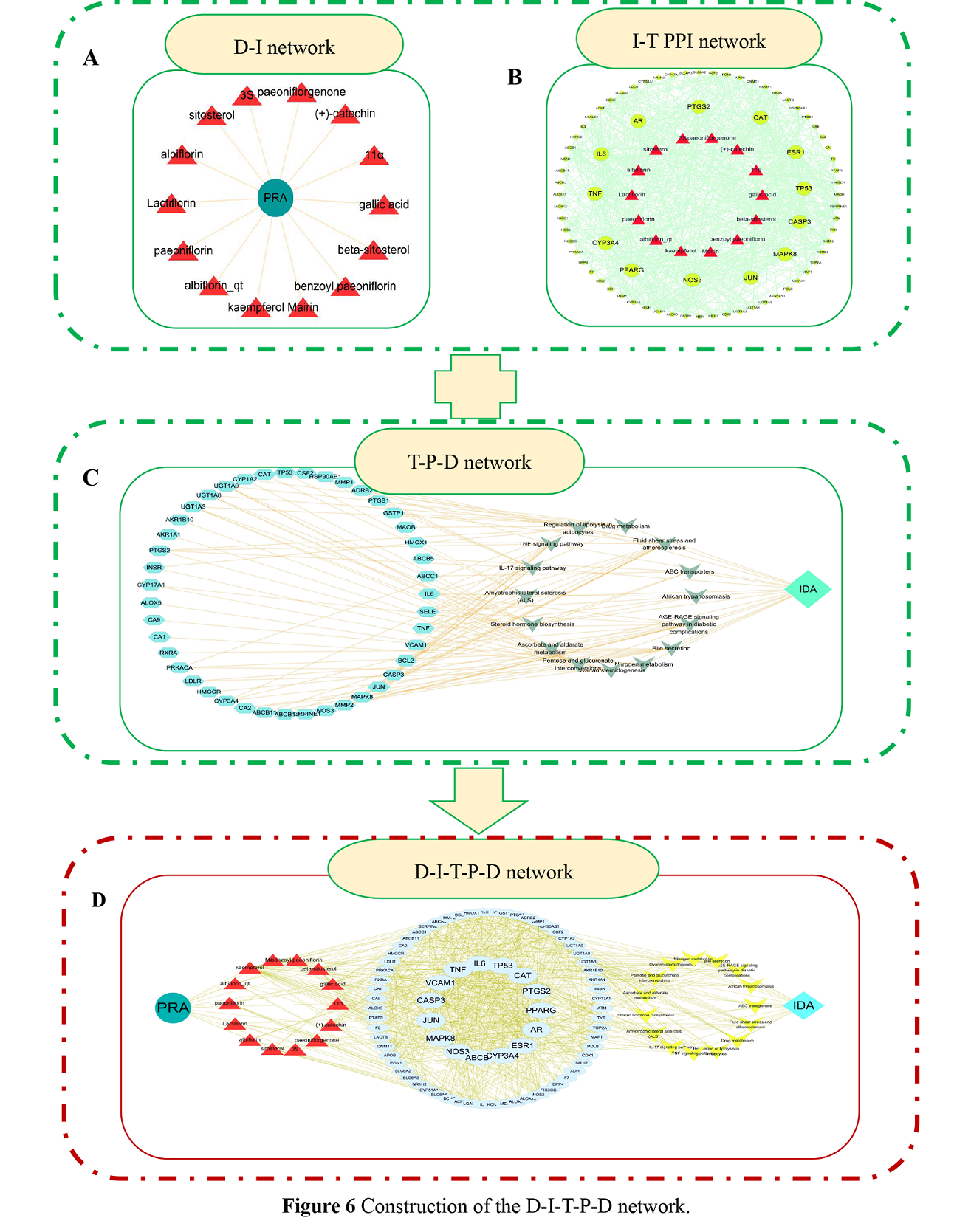

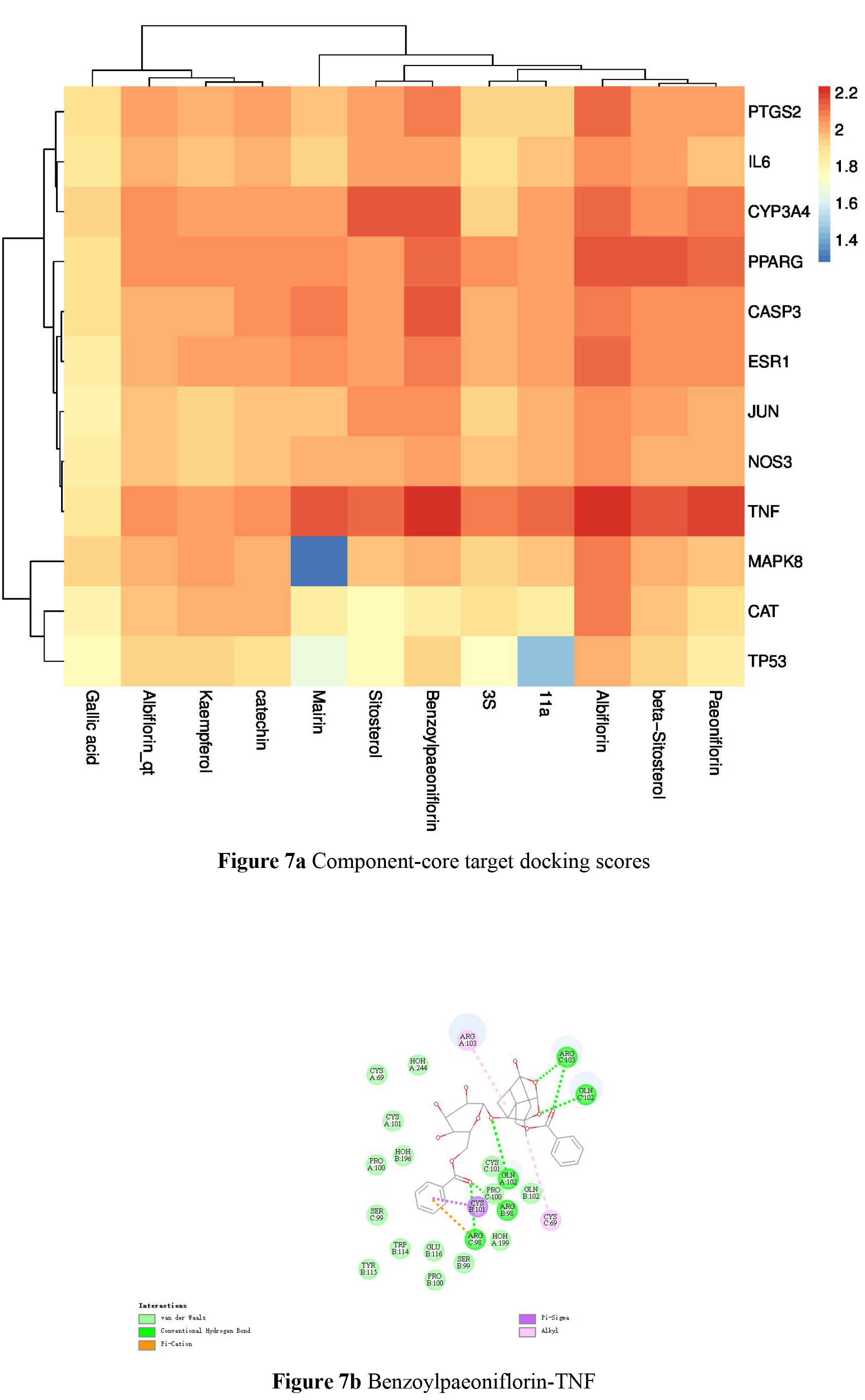


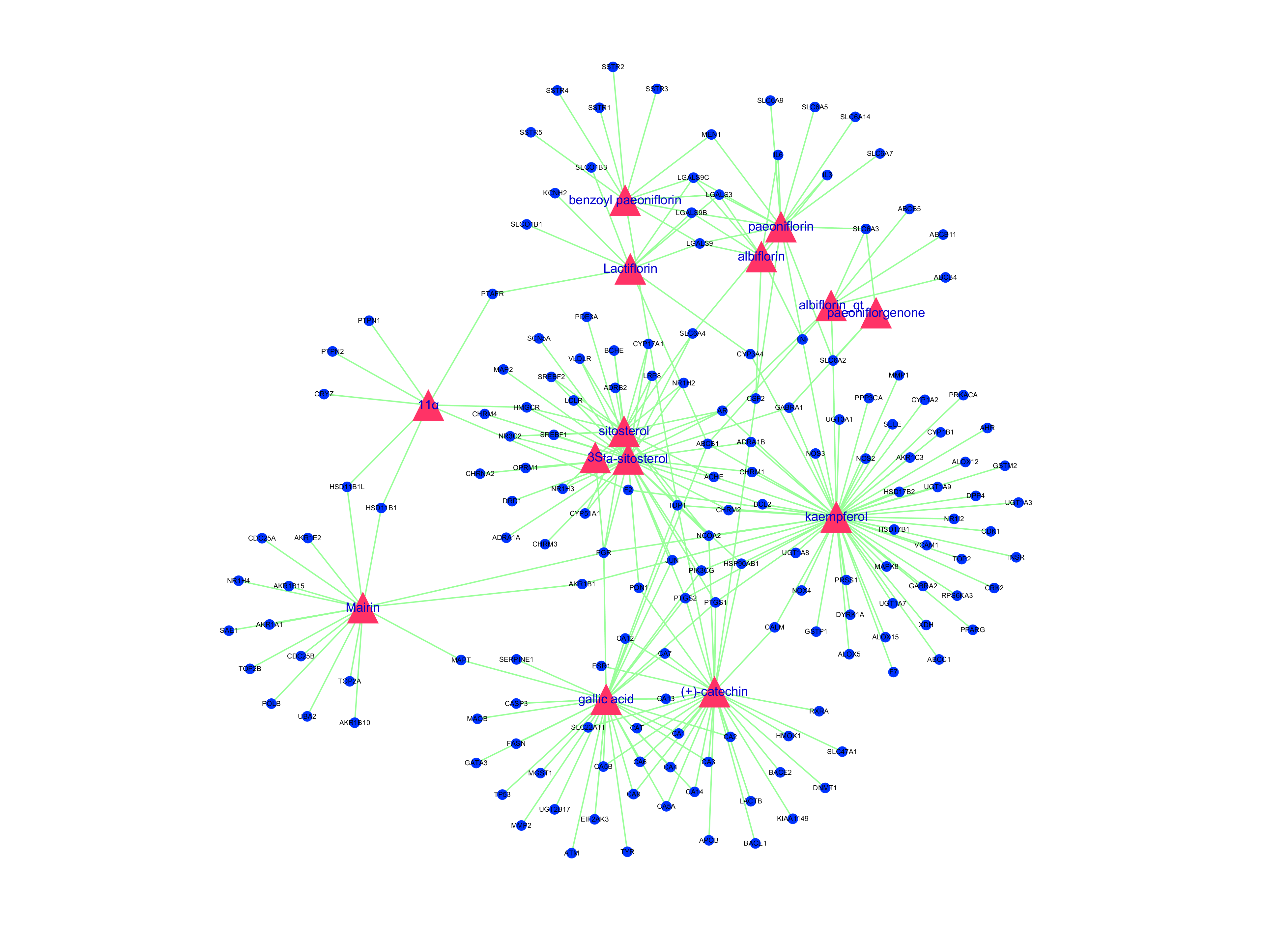
Figure1-I-T


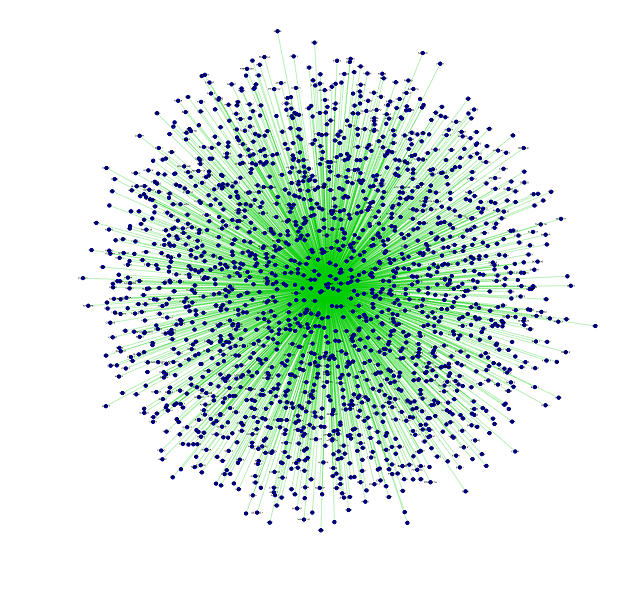
Figure1-IDA-T
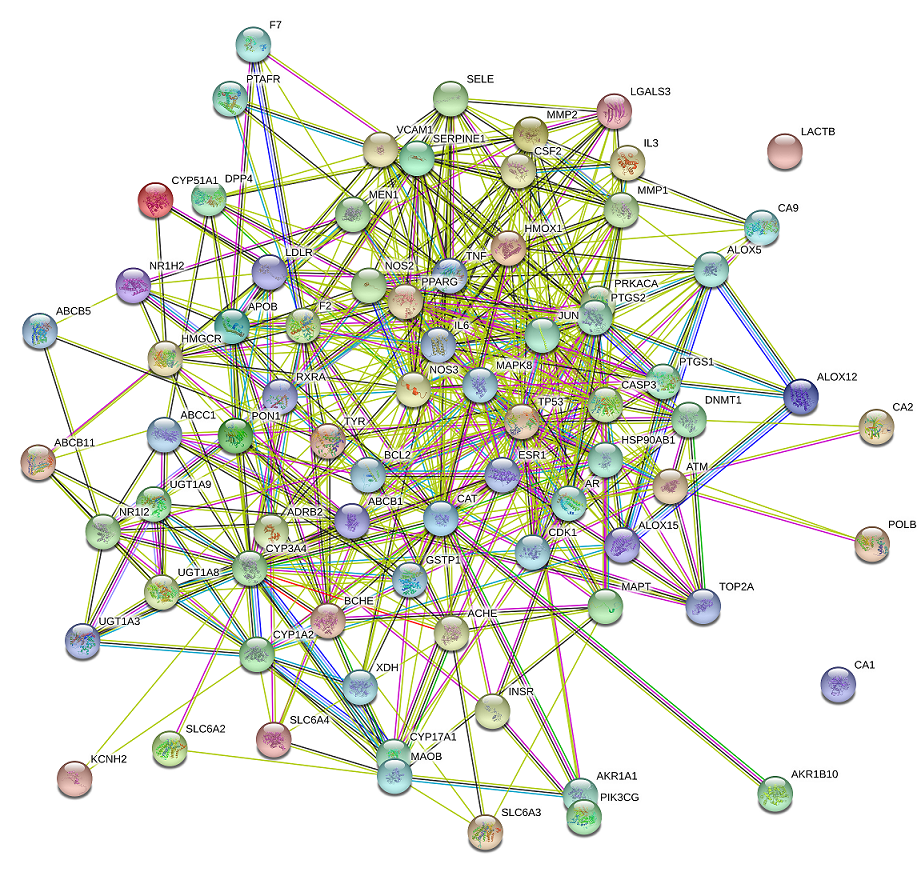
Figure1-PPI
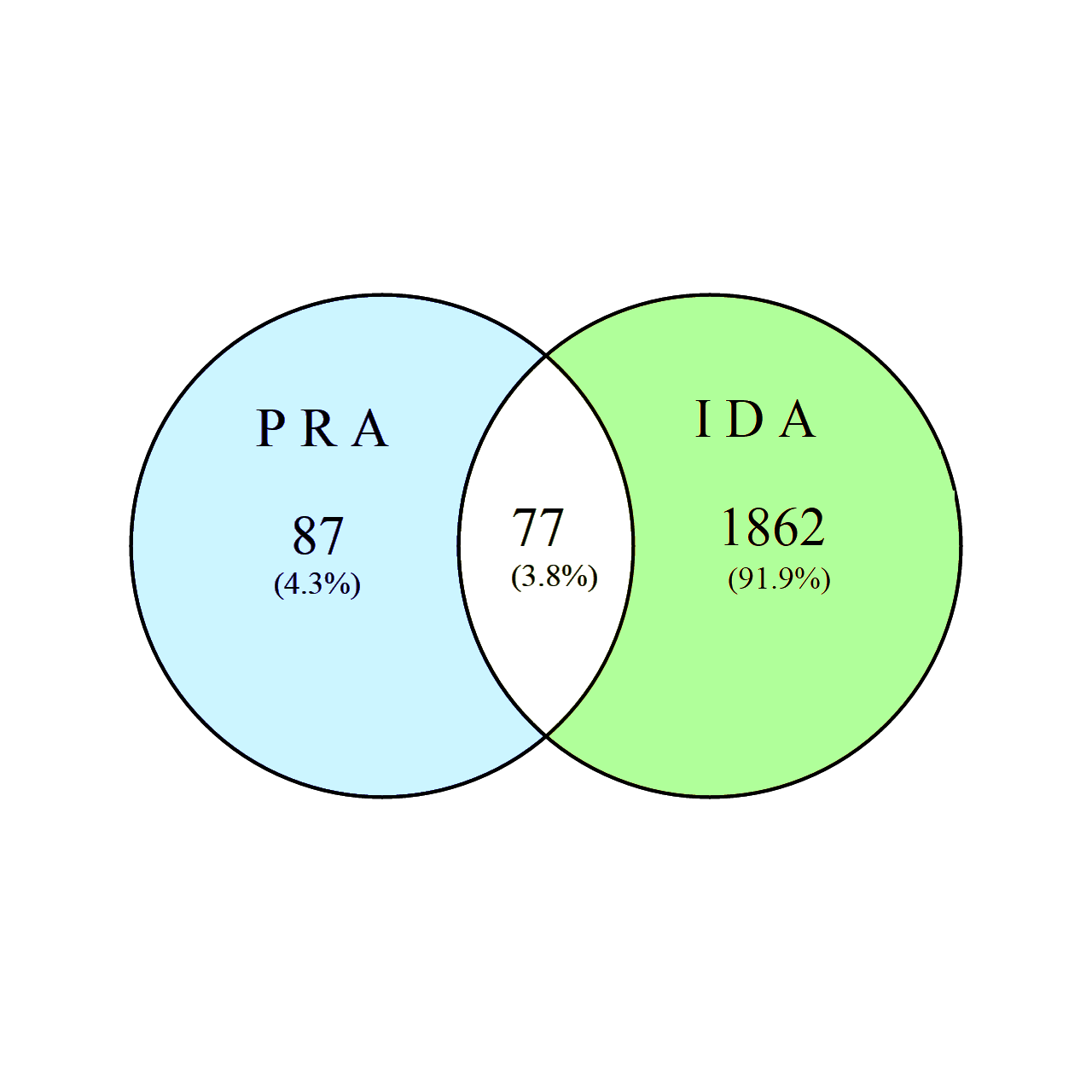
Figure1-Venney


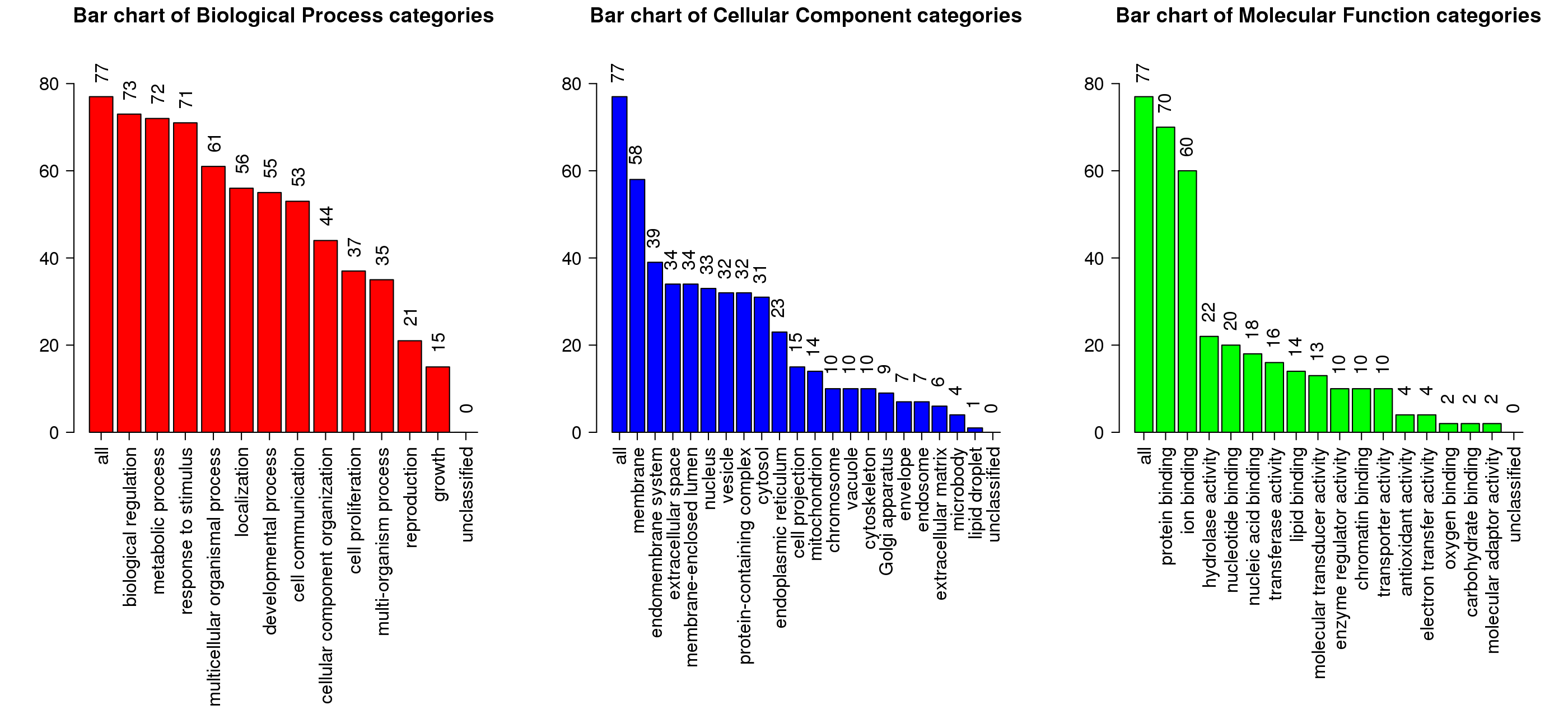
Figure2-D
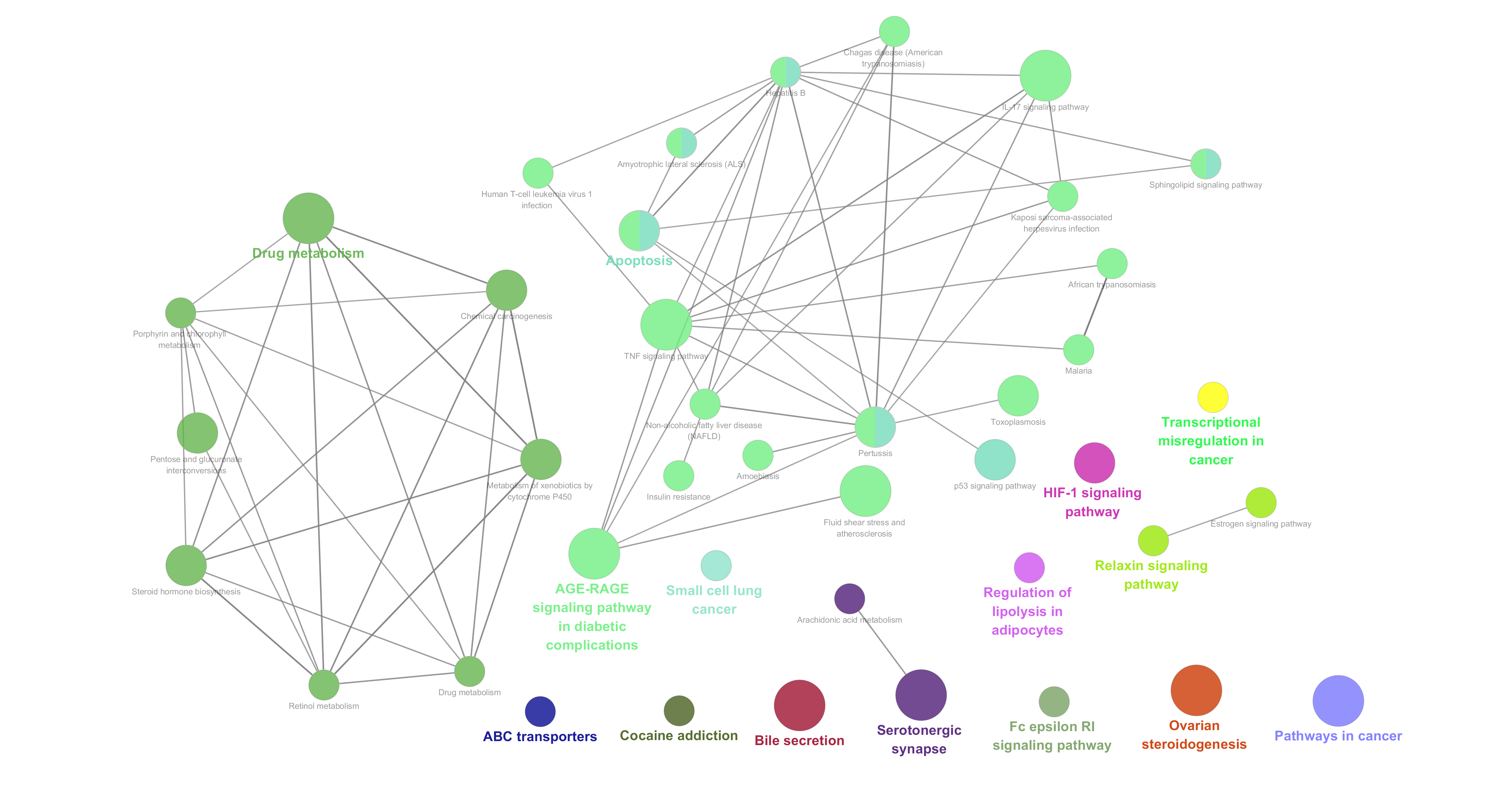
Figure2-KEGG
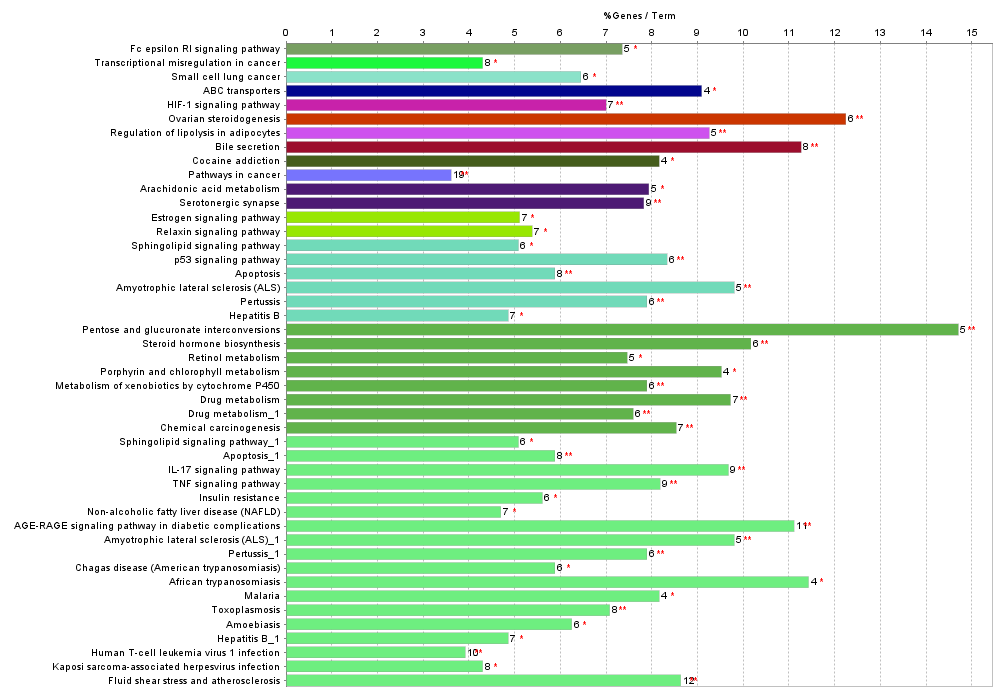
Figure2-KEGG
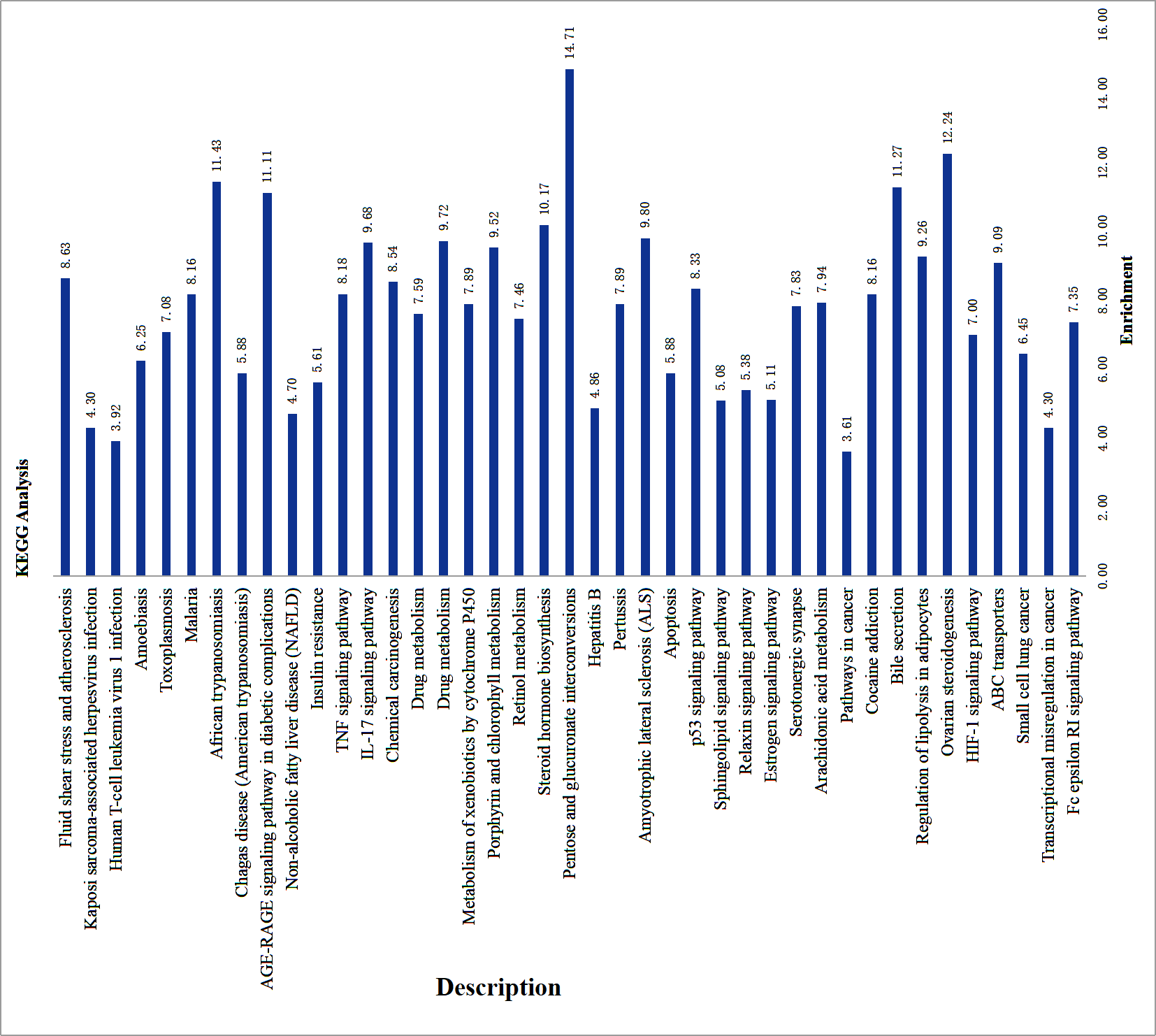
Figure2-E
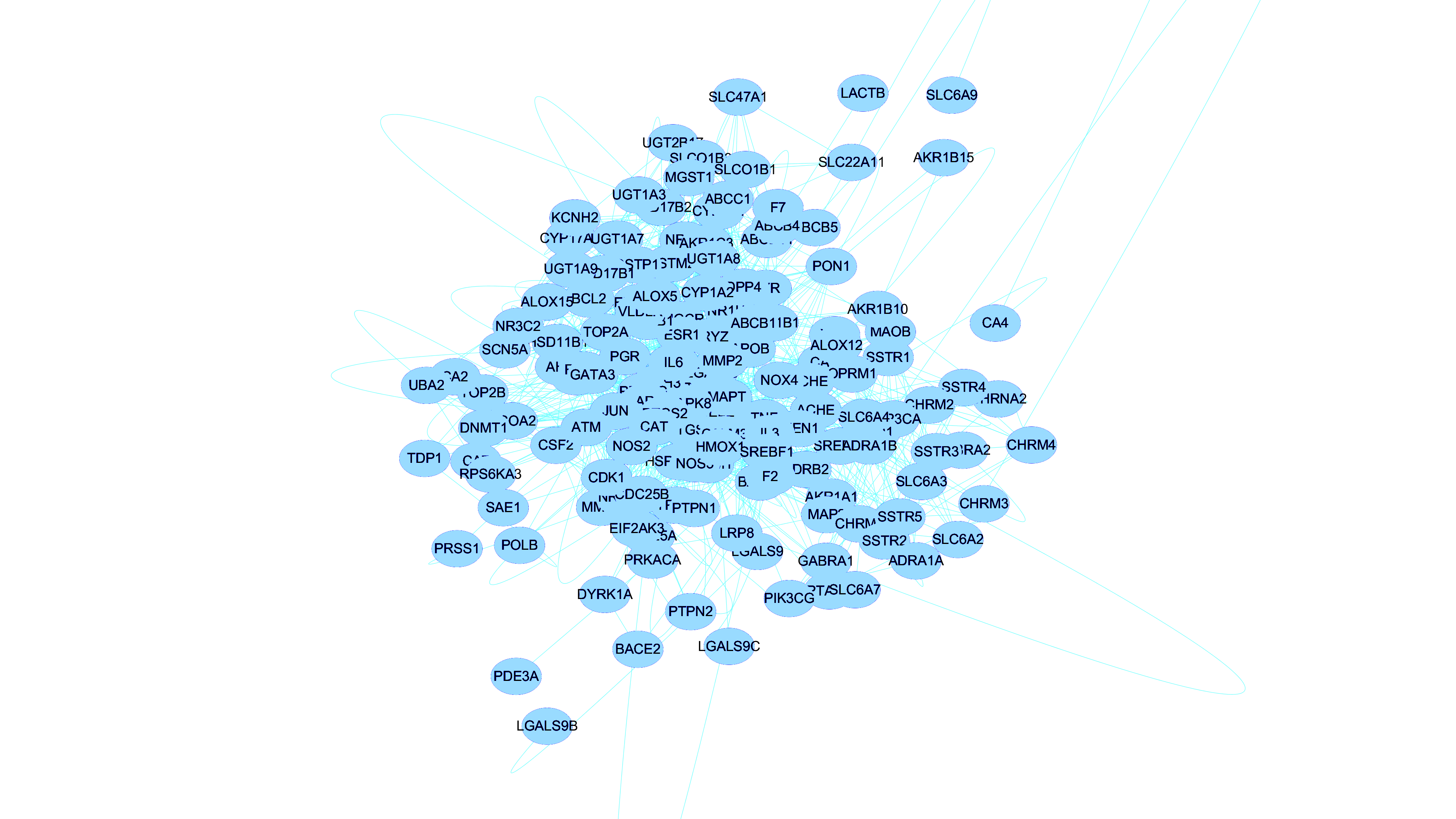
Figure3-A
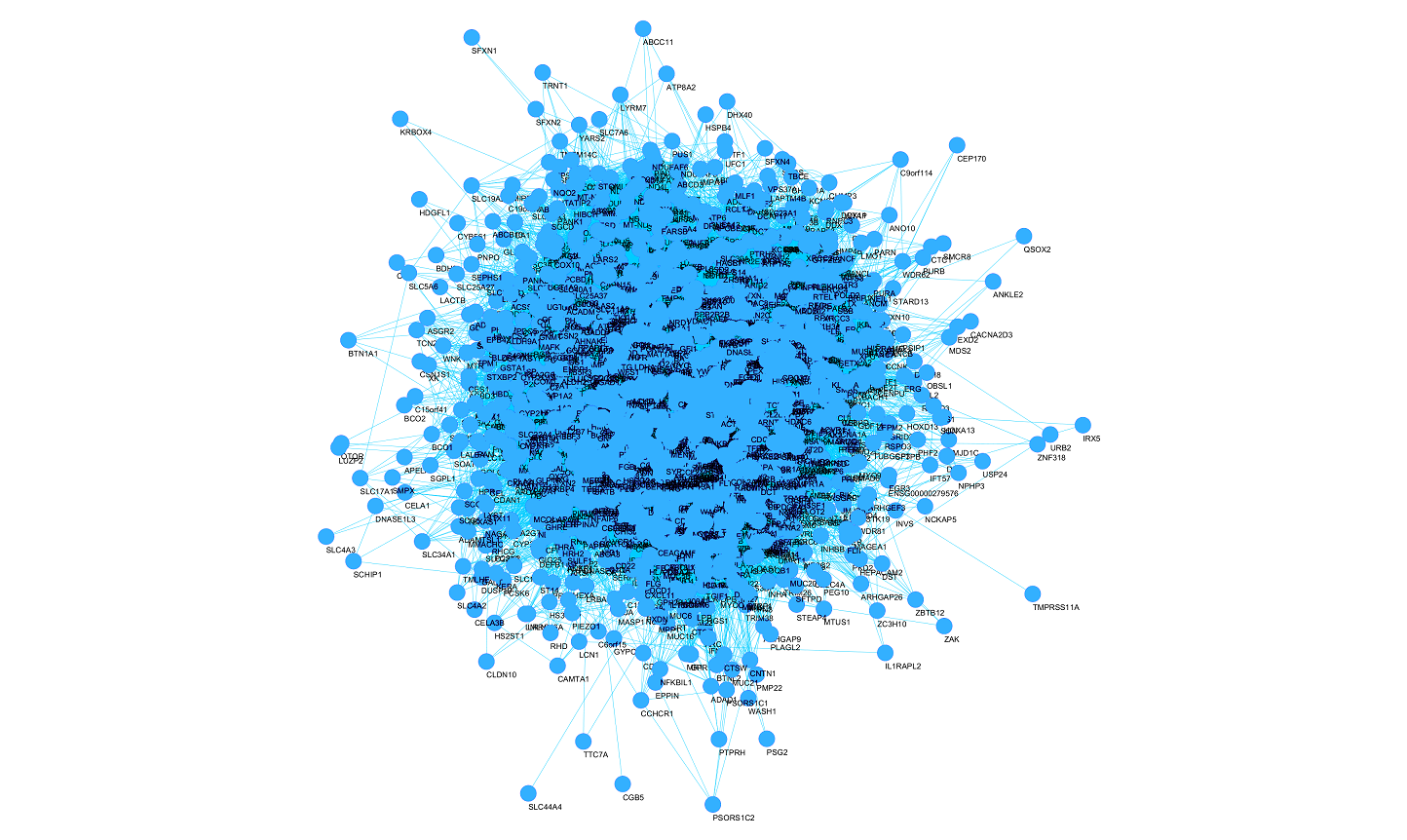
Figure3-B
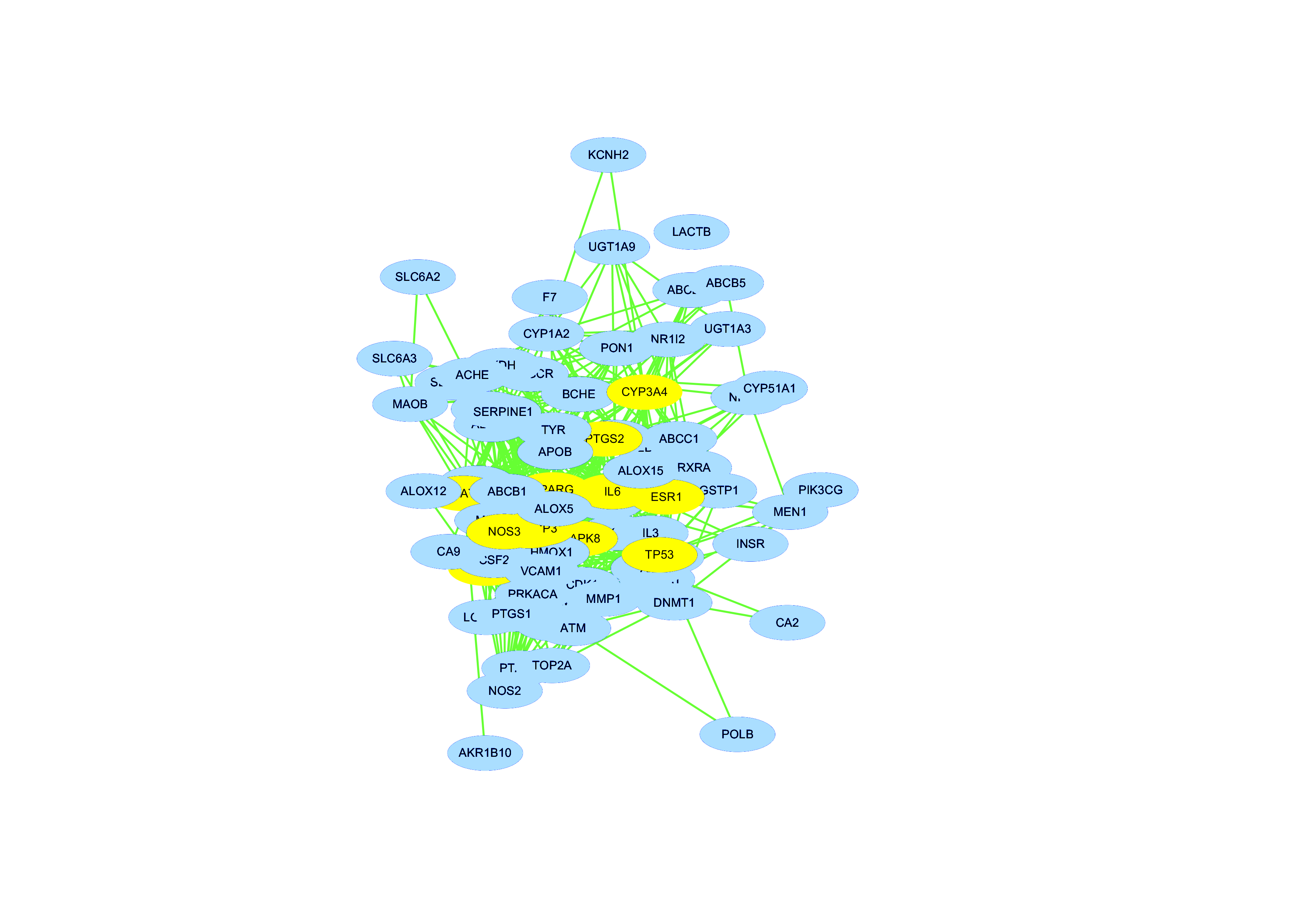
Figure3-C
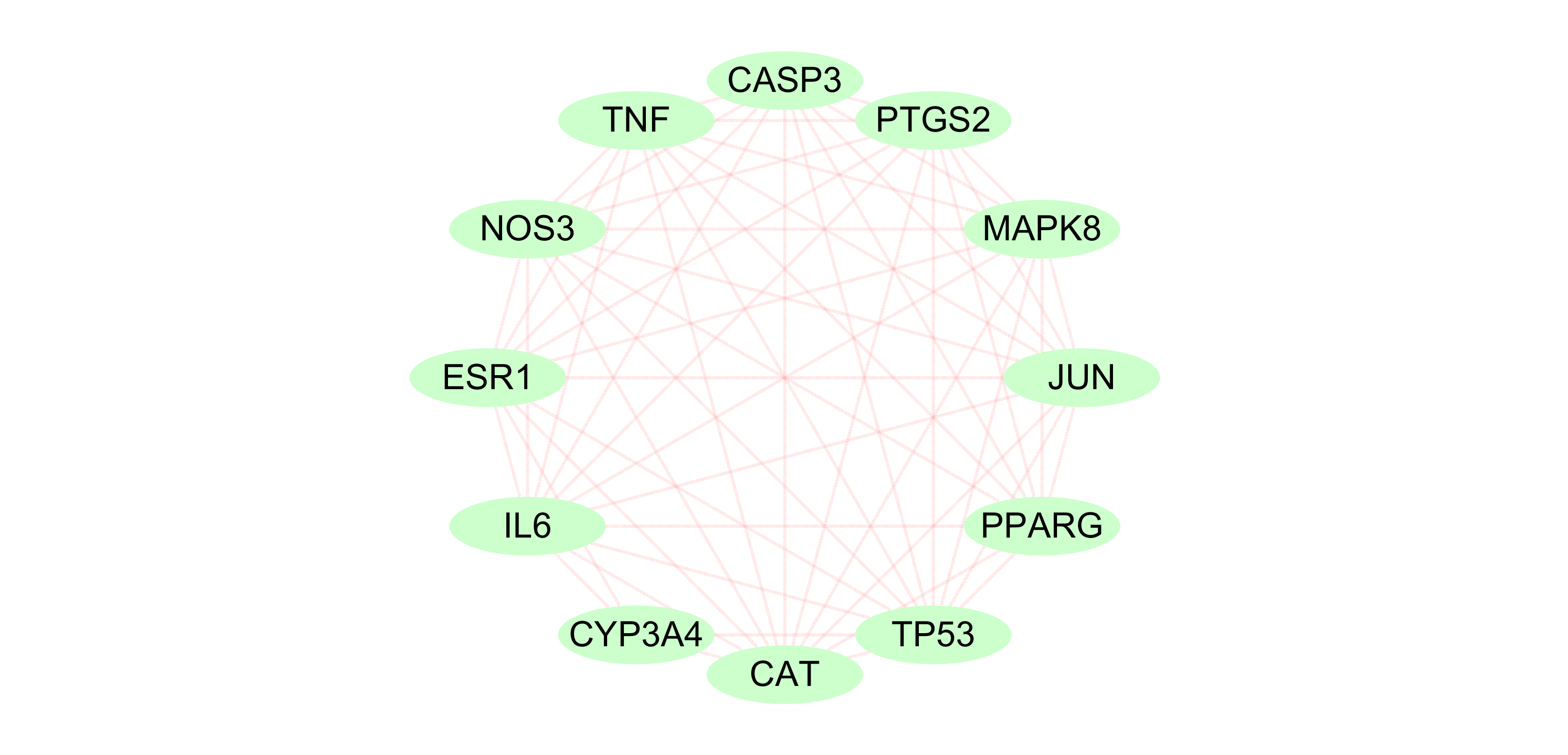
Figure3-D
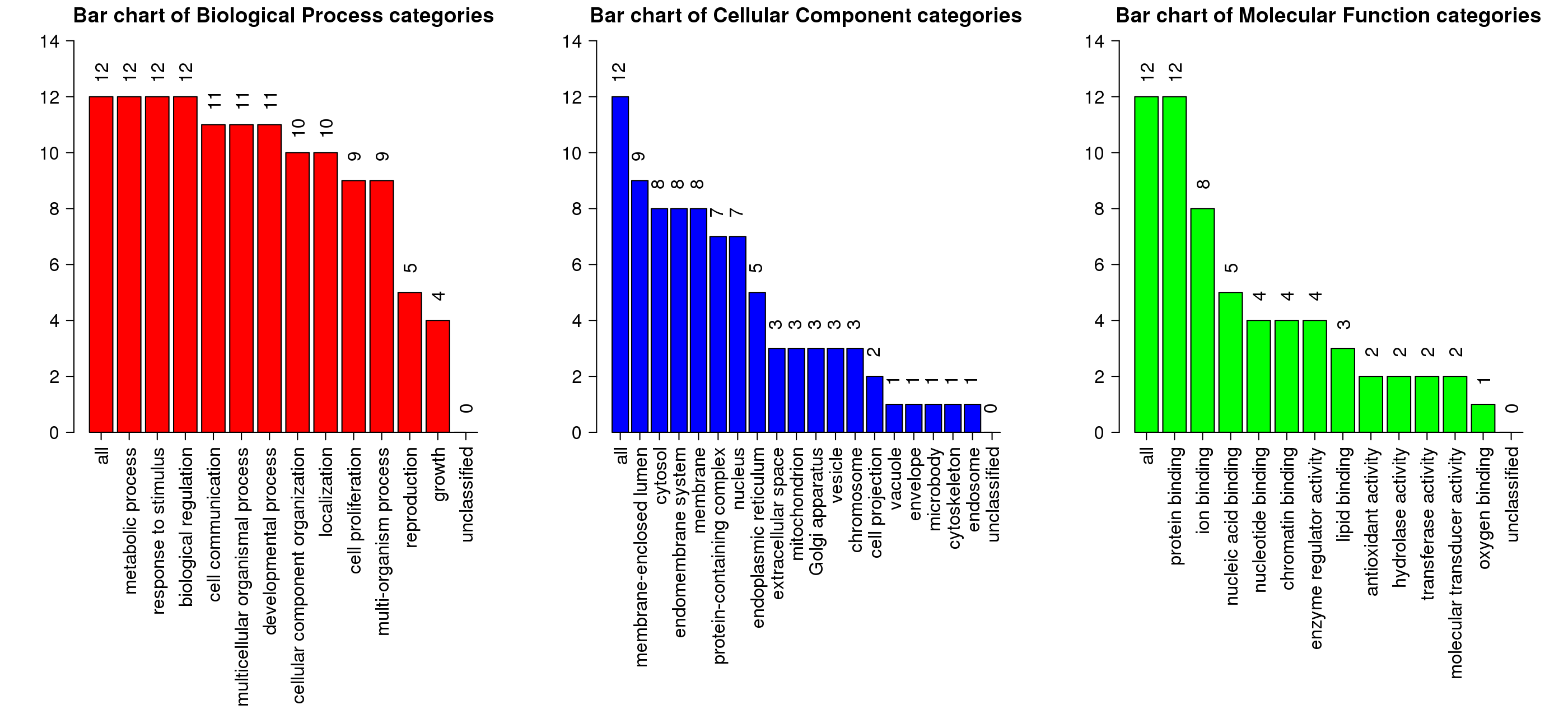
Figure3-E
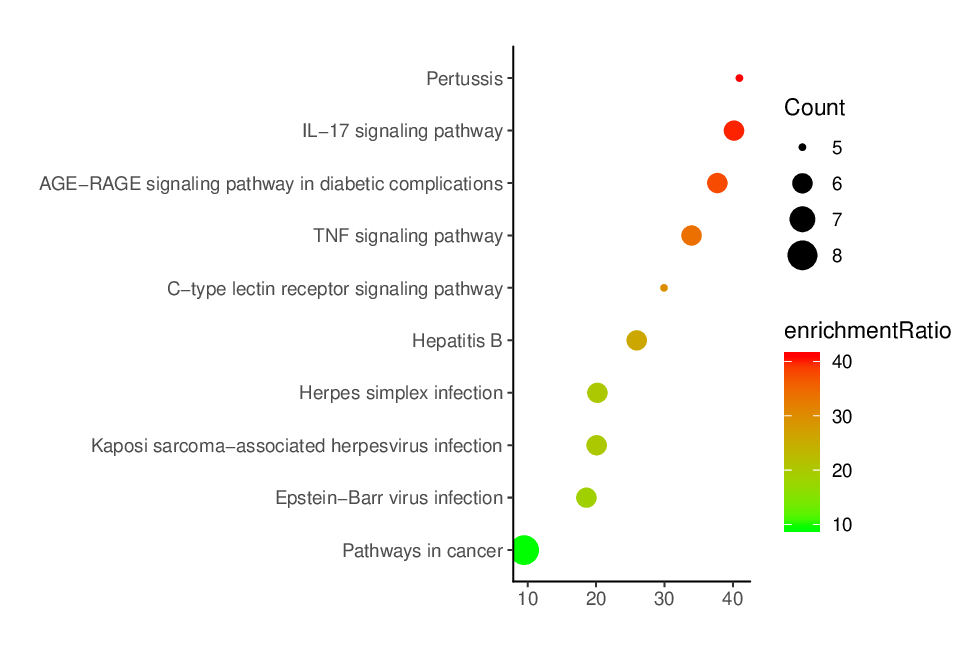
Figure3-F


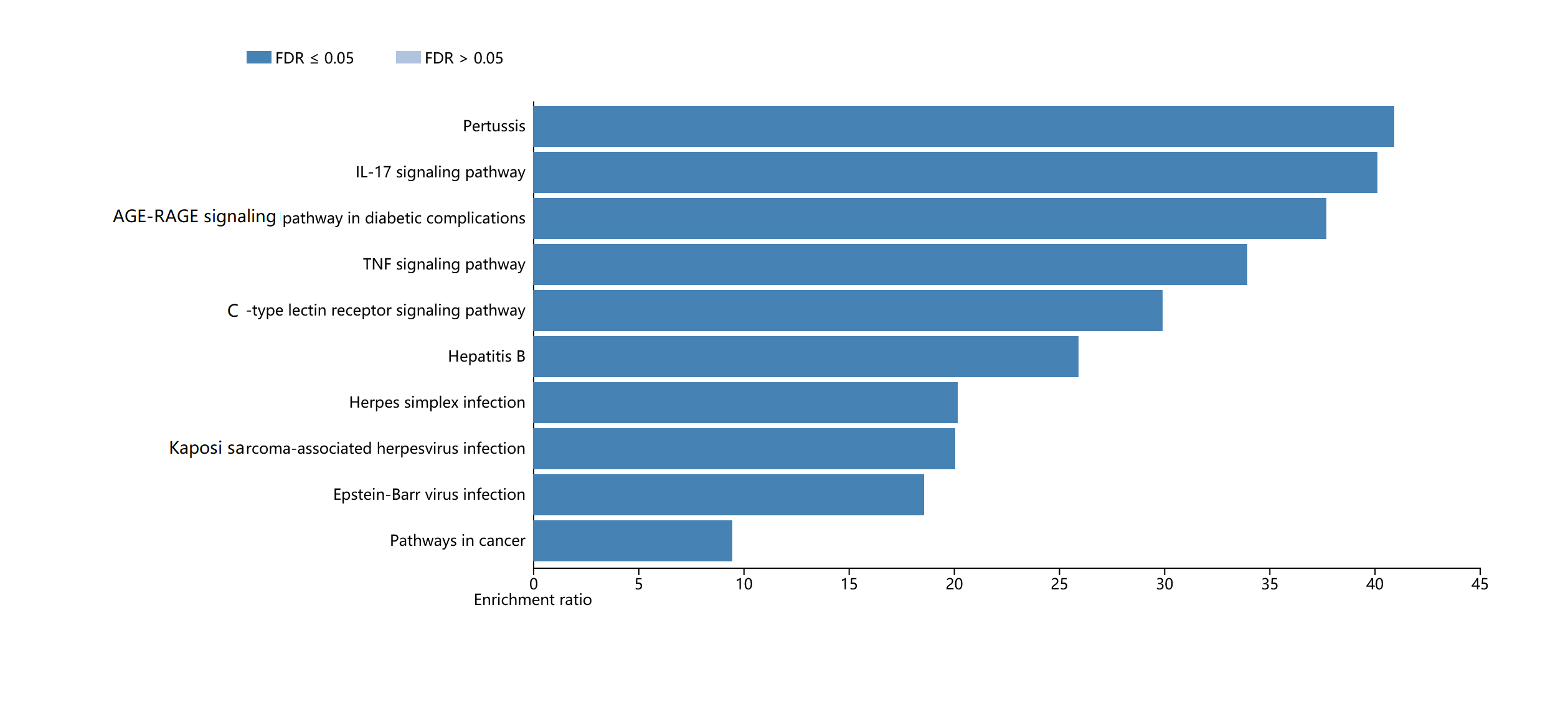


Figure3-F
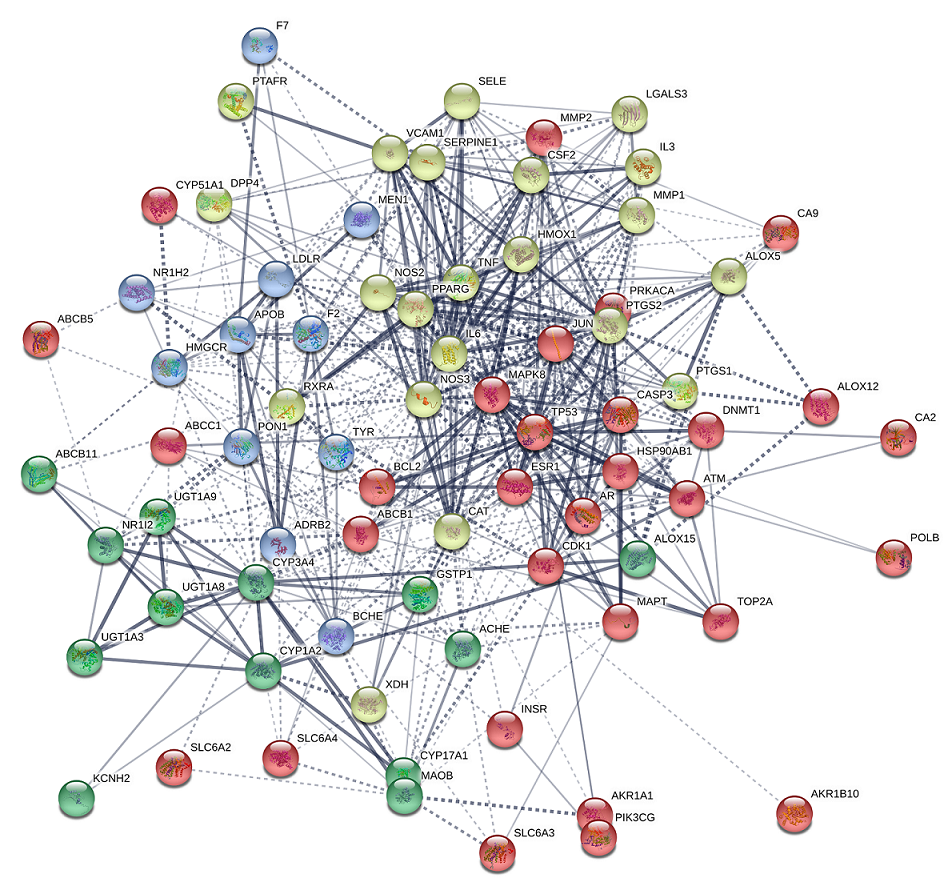
Figure5-A
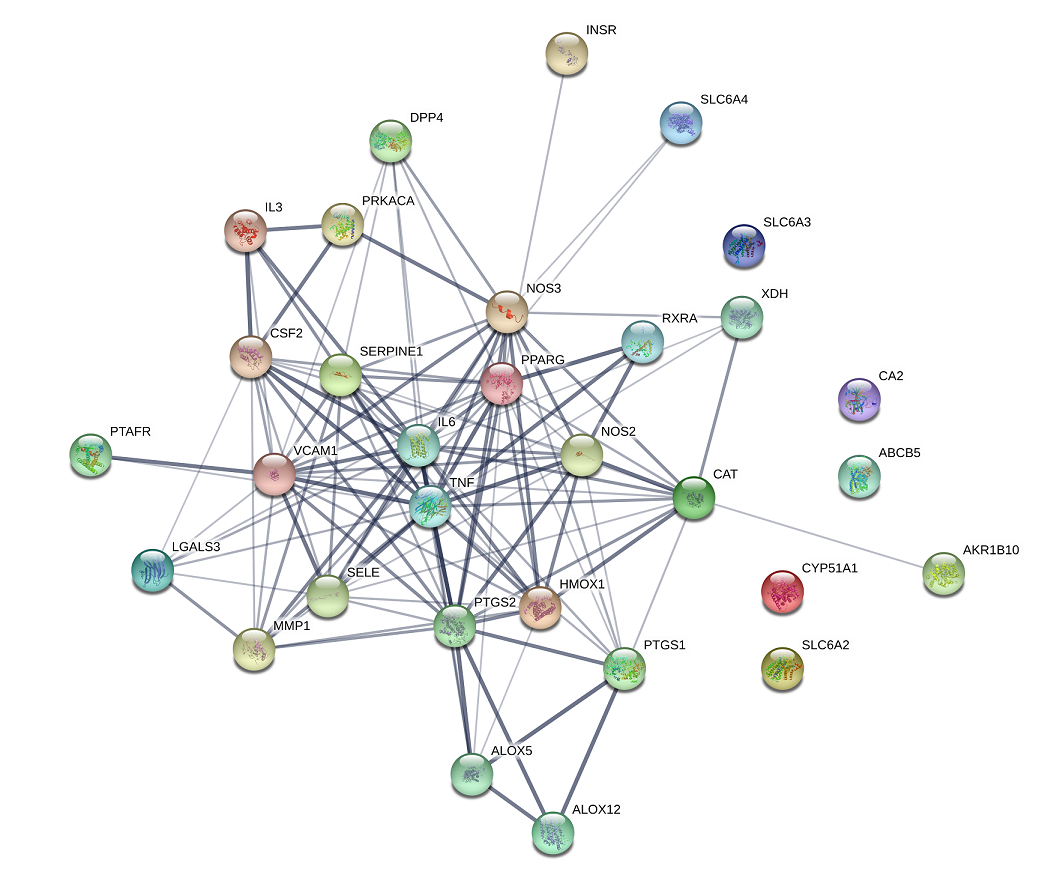
Figure5-B-a
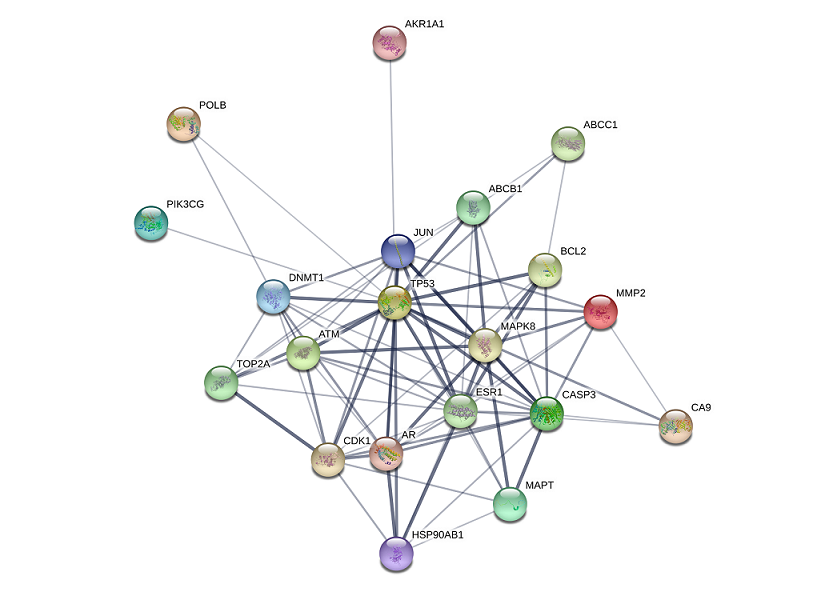
Figure5-B-b
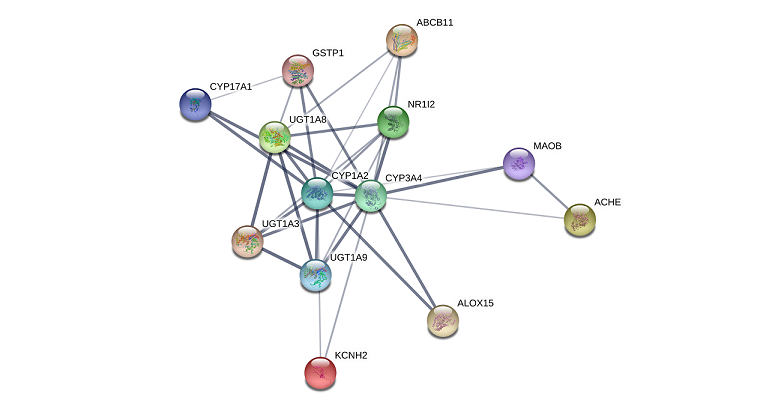
Figure5-B-c
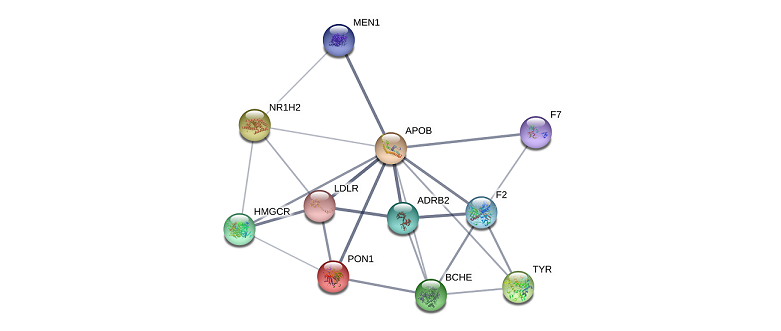
Figure5-B-d


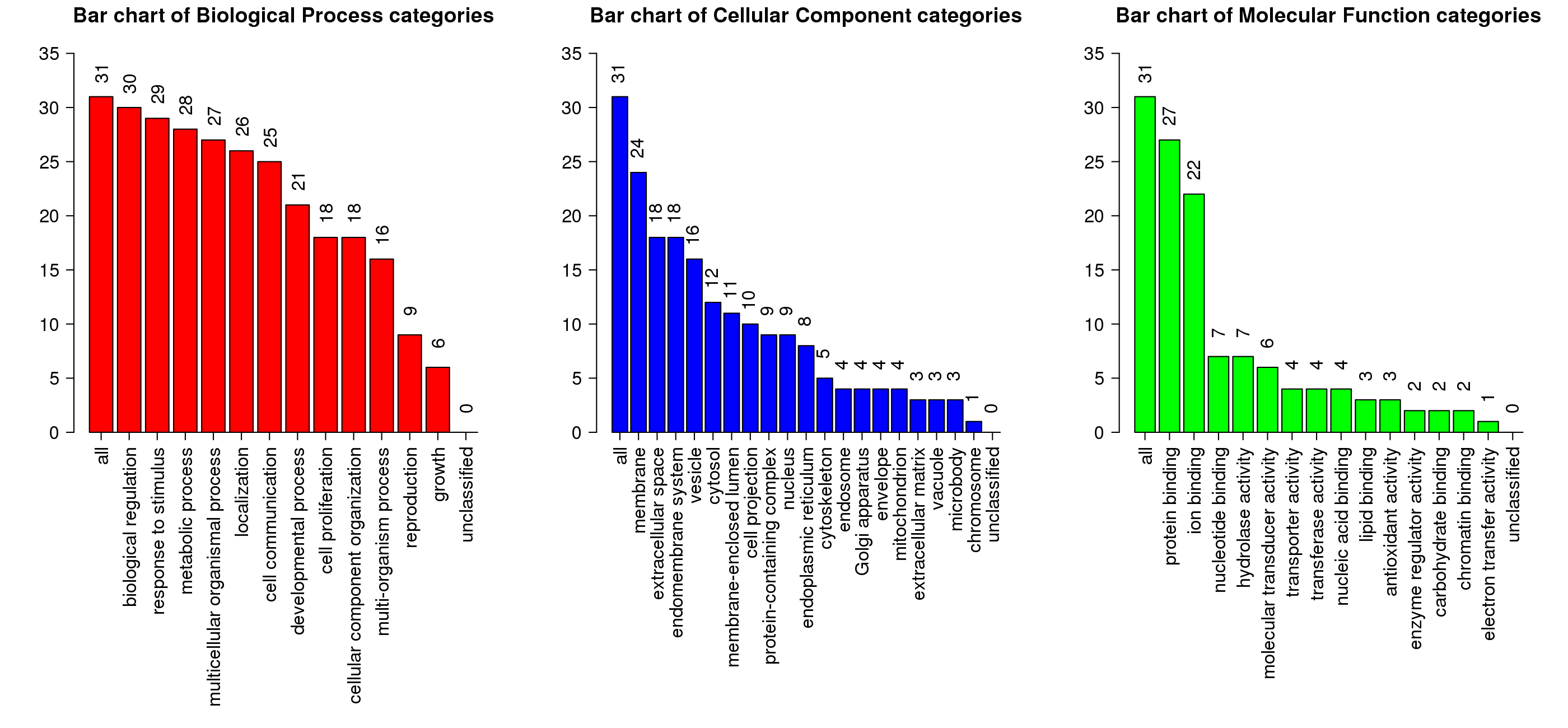
Figure5-C-a
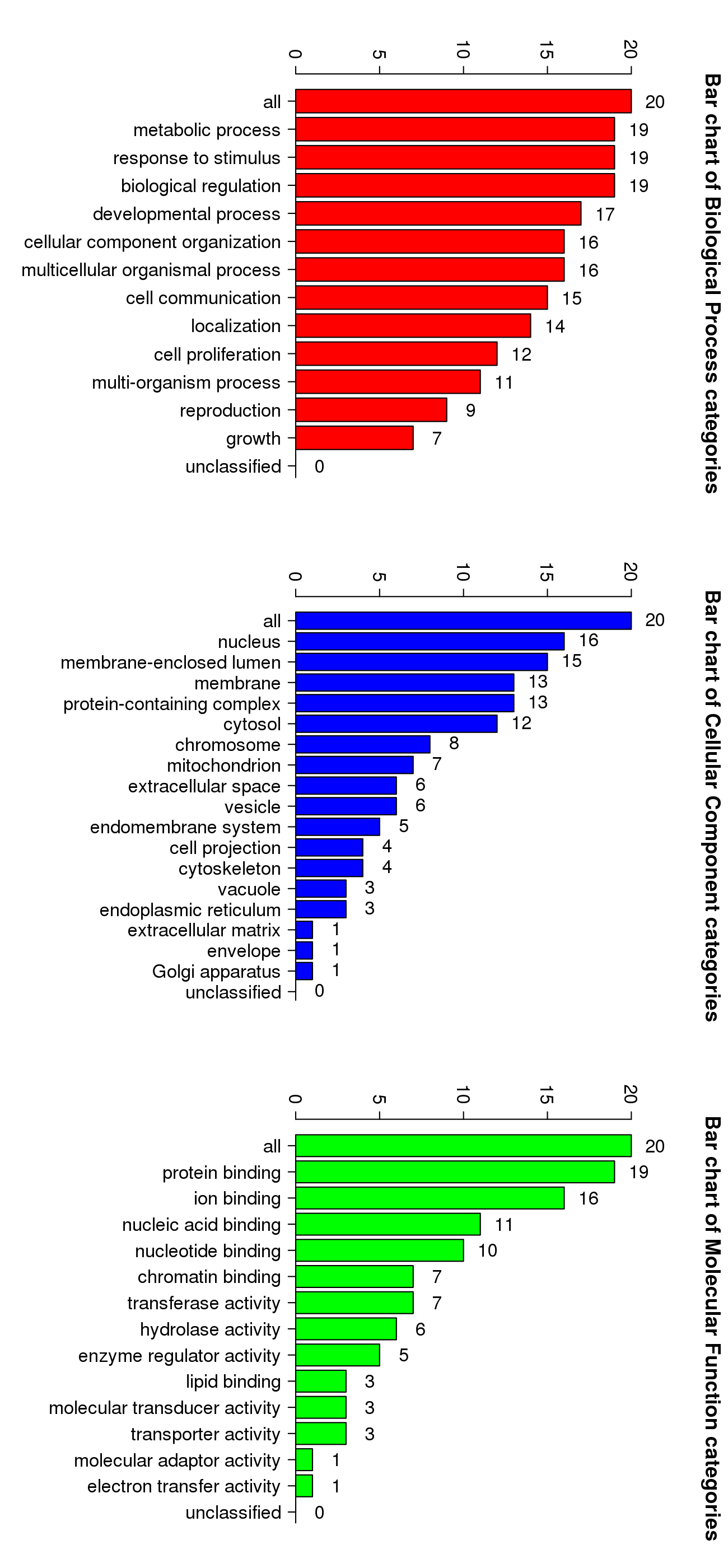
Figure5-C-b
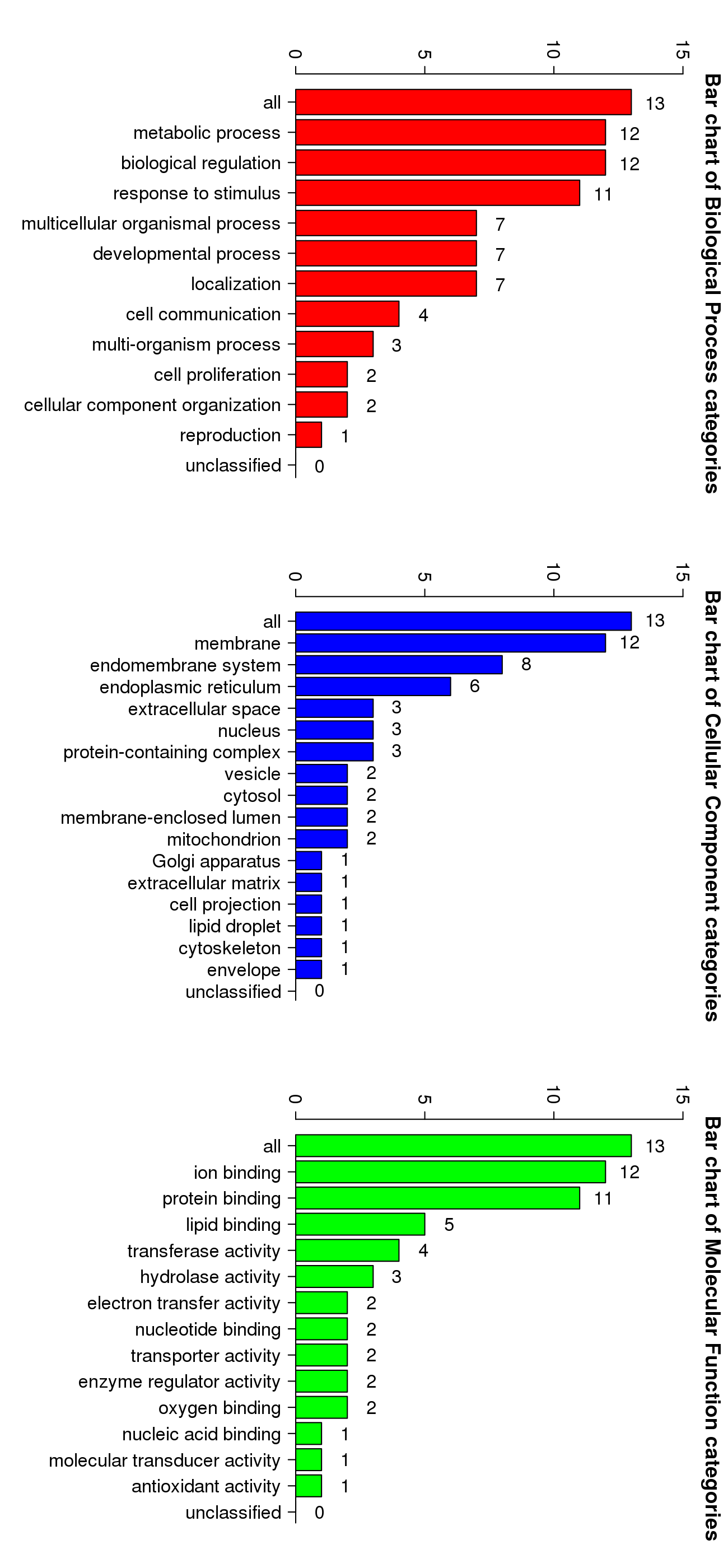
Figure5-C-c
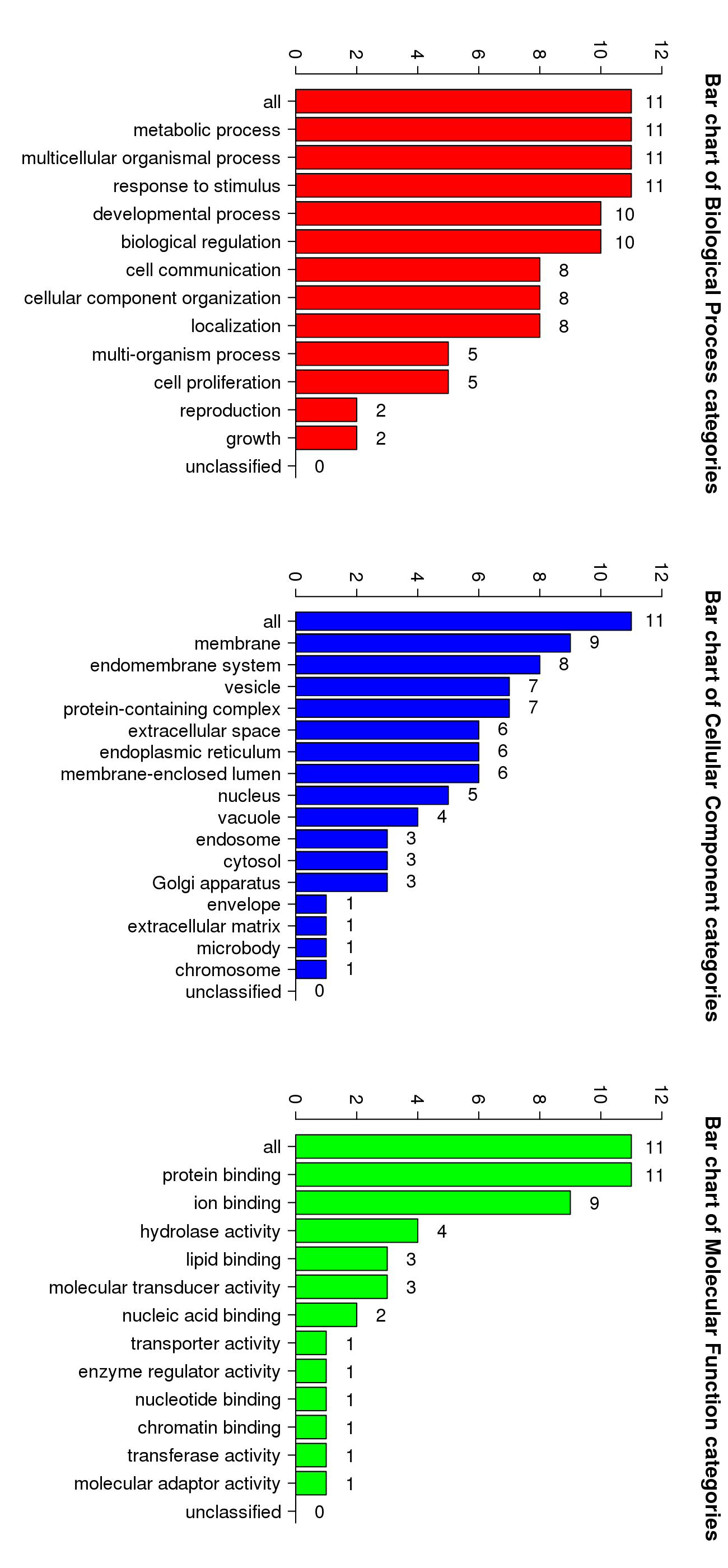
Figure5-C-d


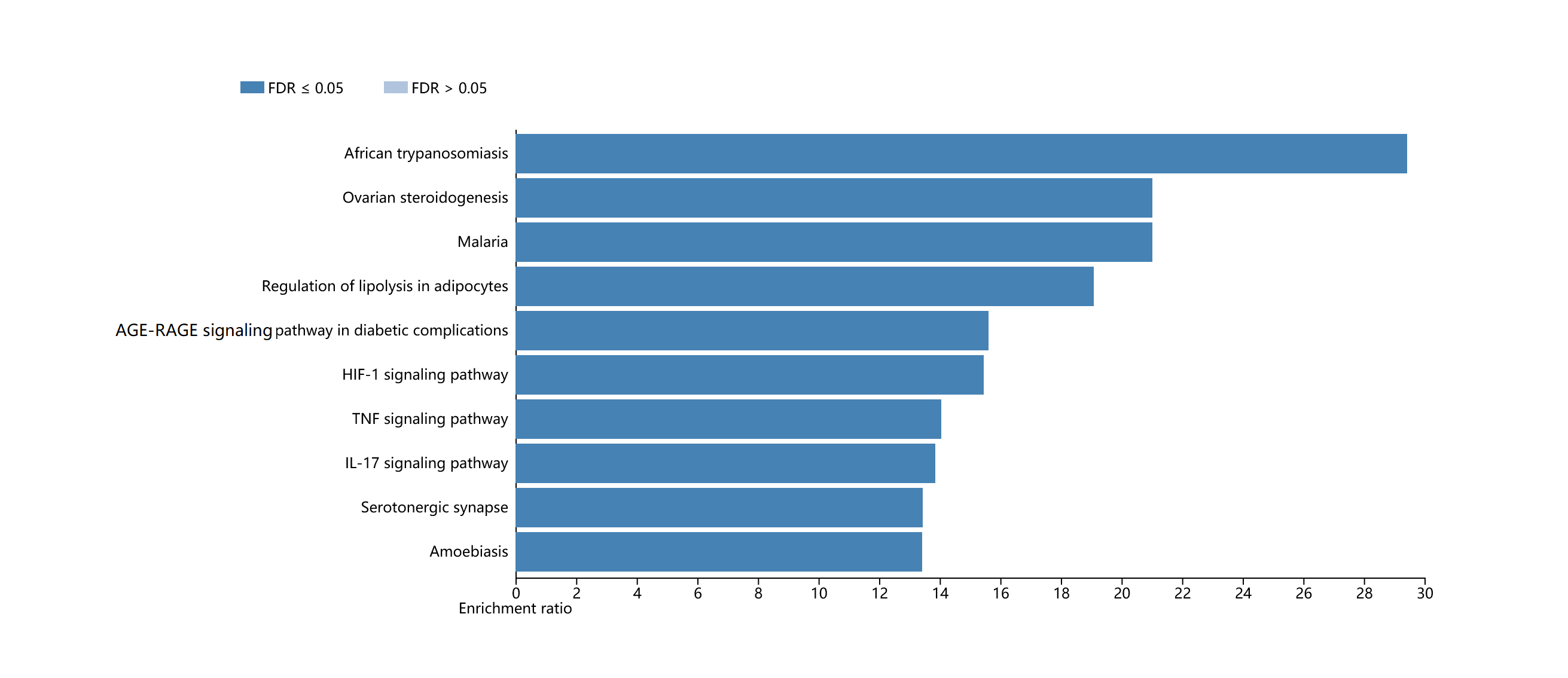


Figure5-D-a


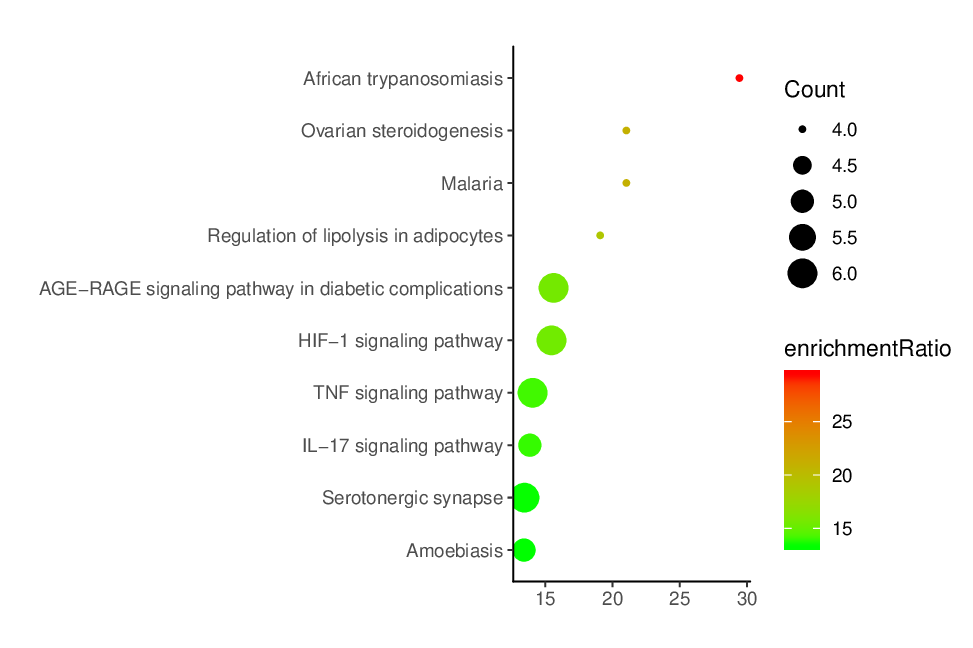
Figure5-D-a
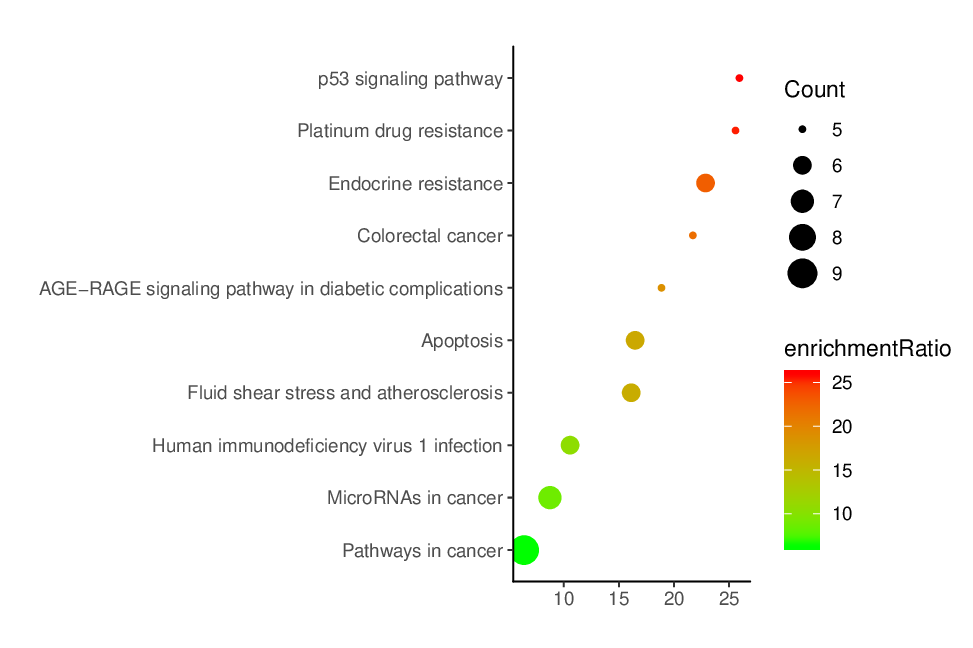
Figure5-D-b


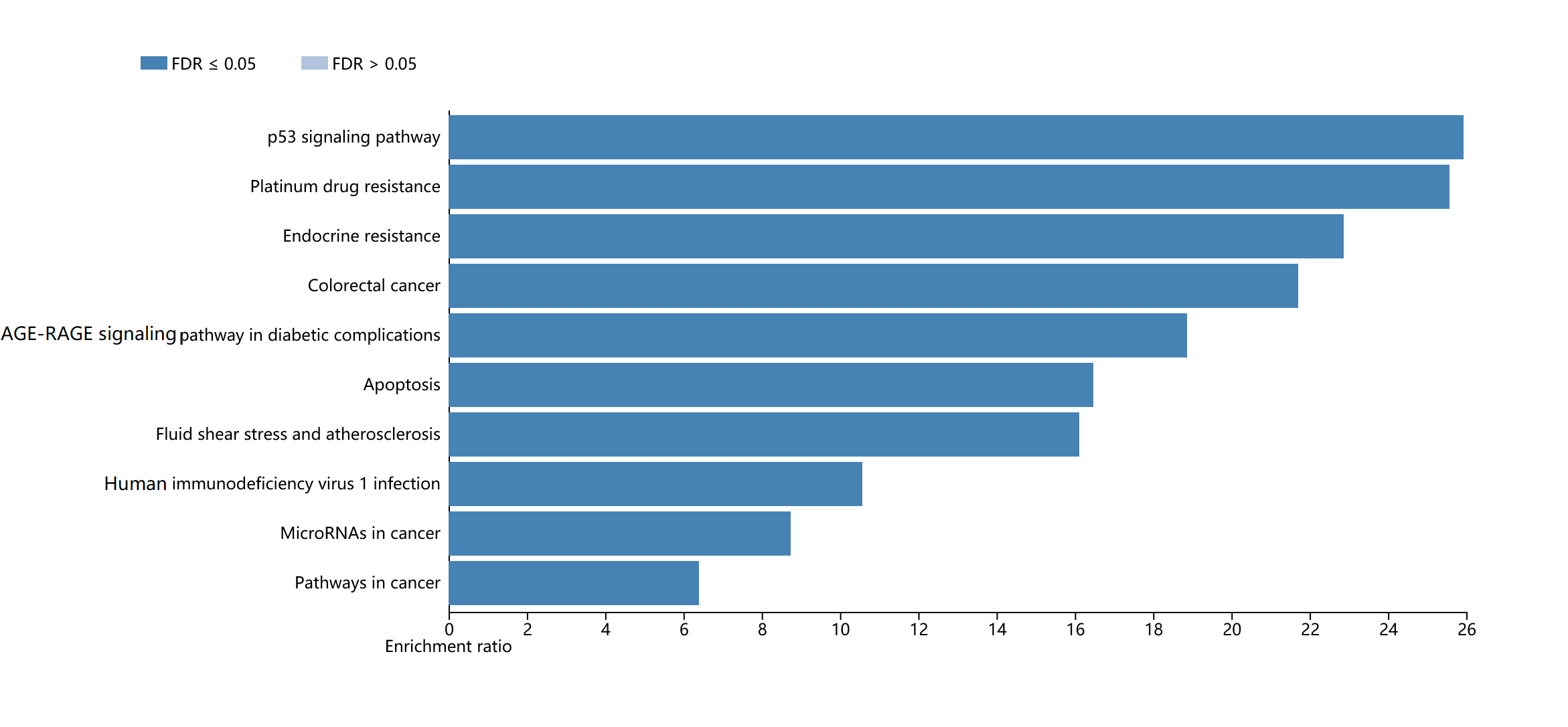


Figure5-D-b


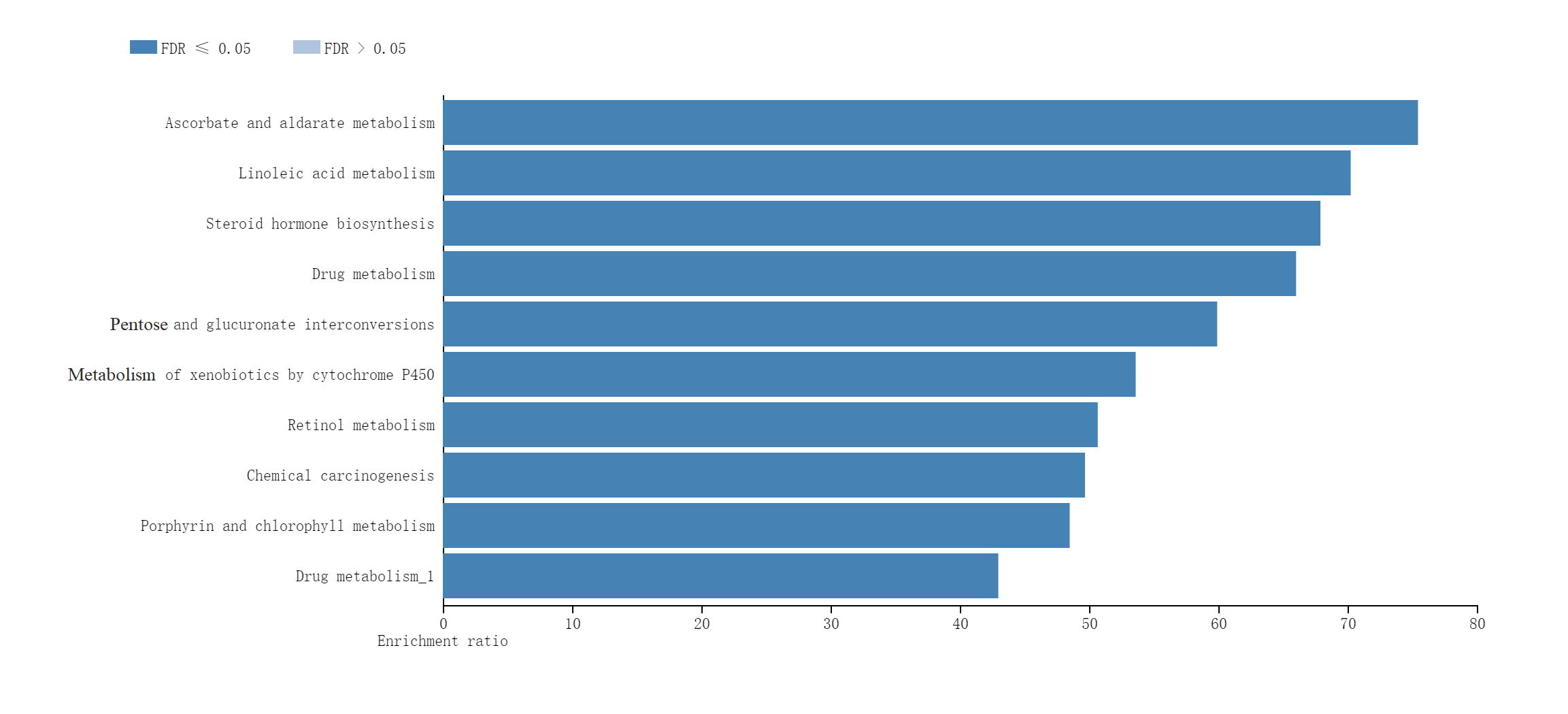


Figure5-D-c
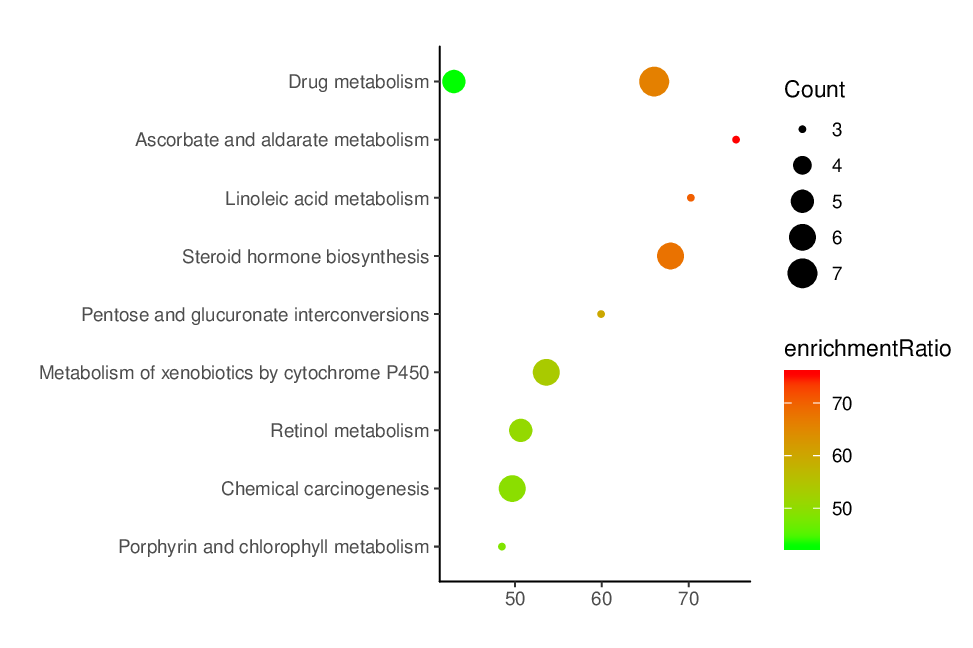
Figure5-D-c
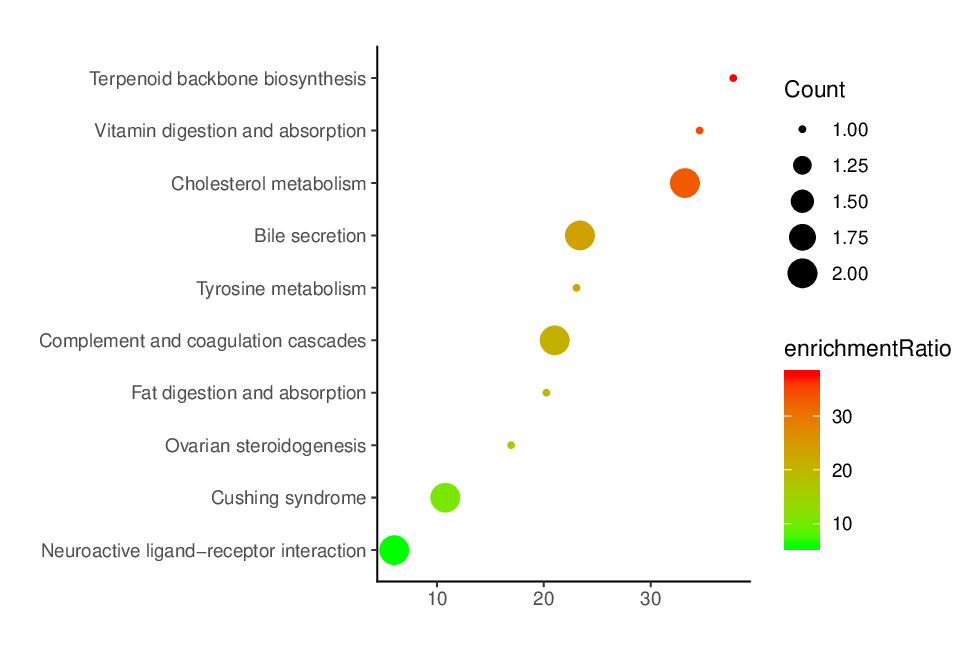
Figure5-D-d


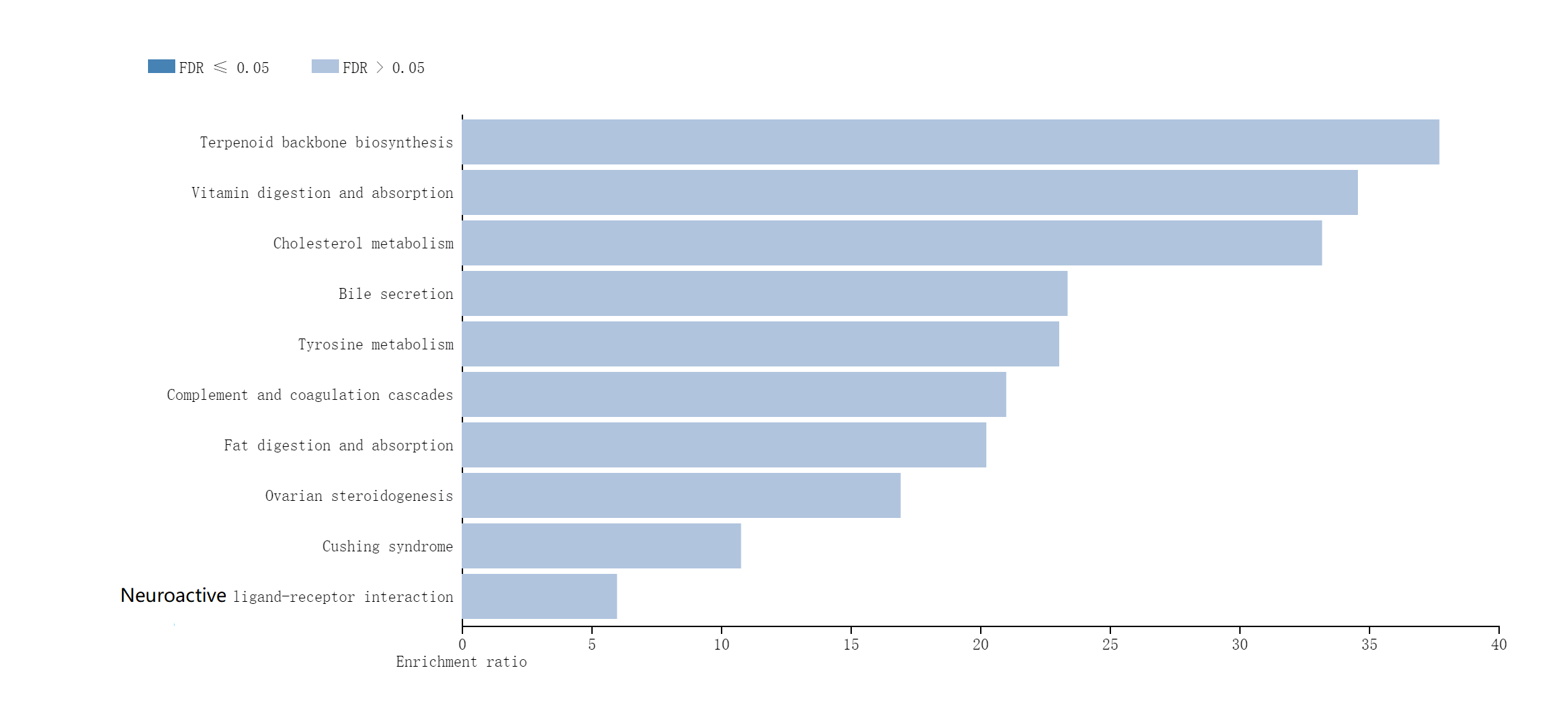


Figure5-D-d
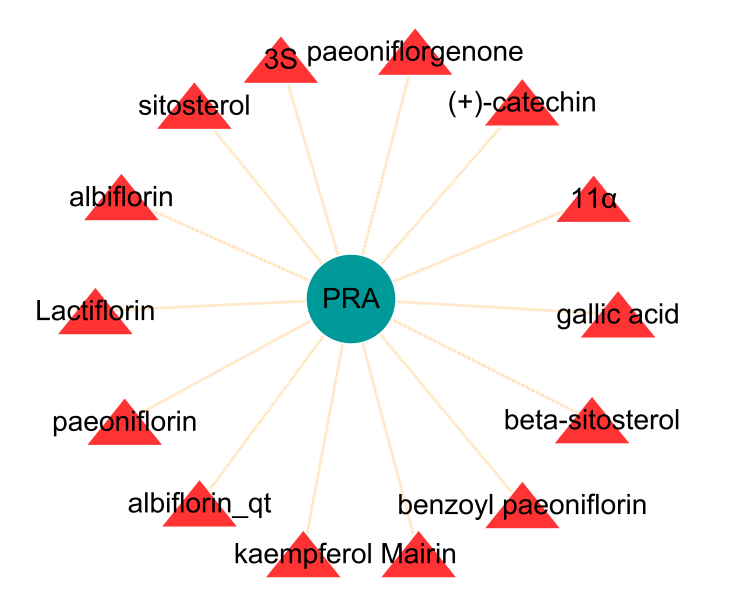
Figure6-A
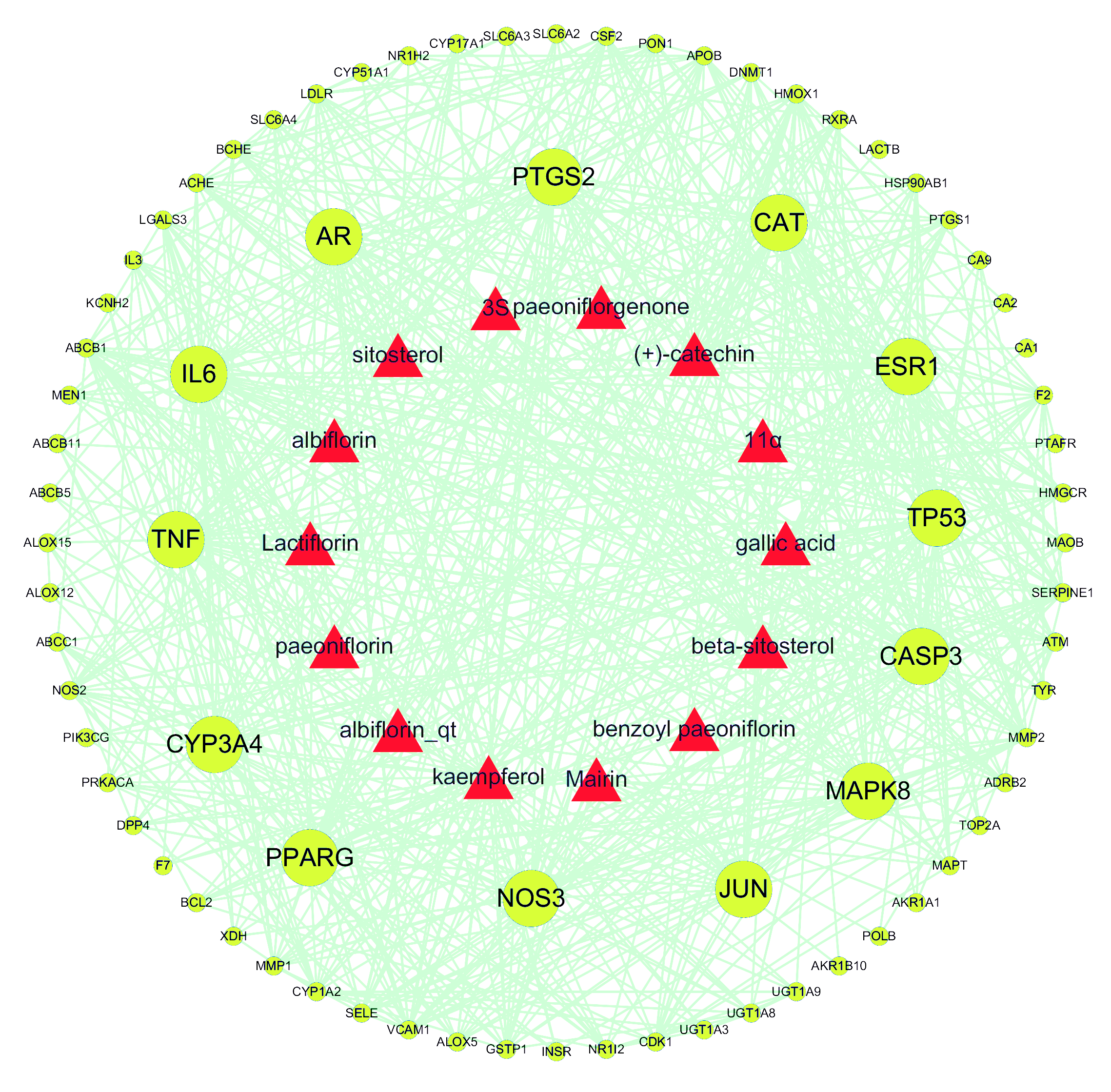
Figure6-B
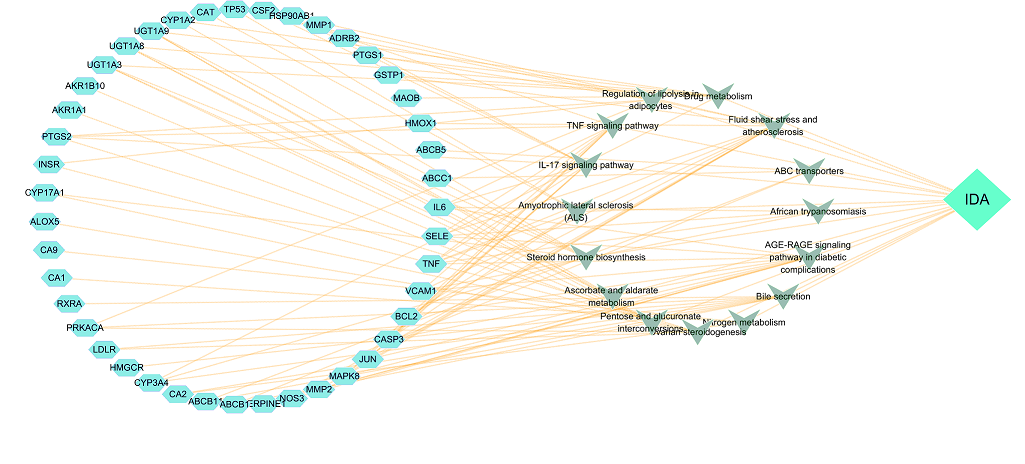
Figure6-C
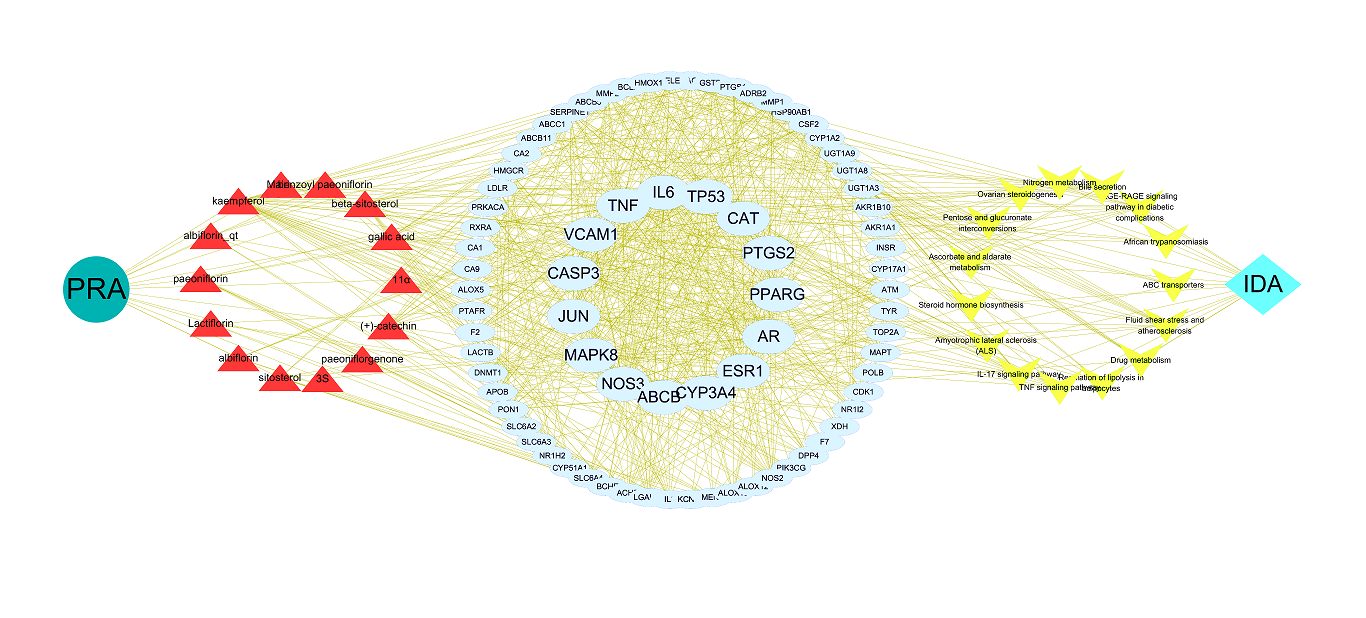
Figure6-D

**Core targets analysis-KEGG**


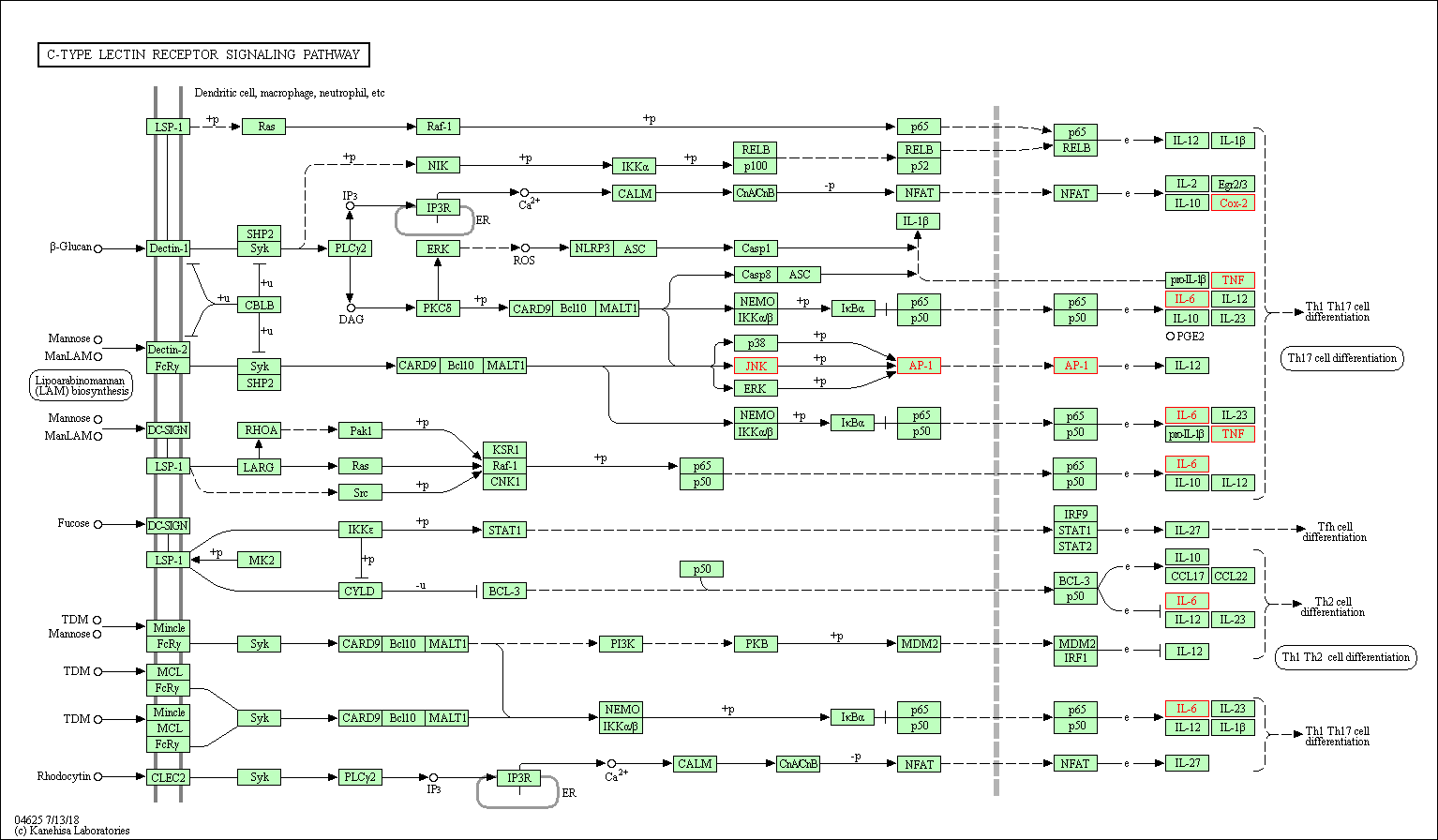

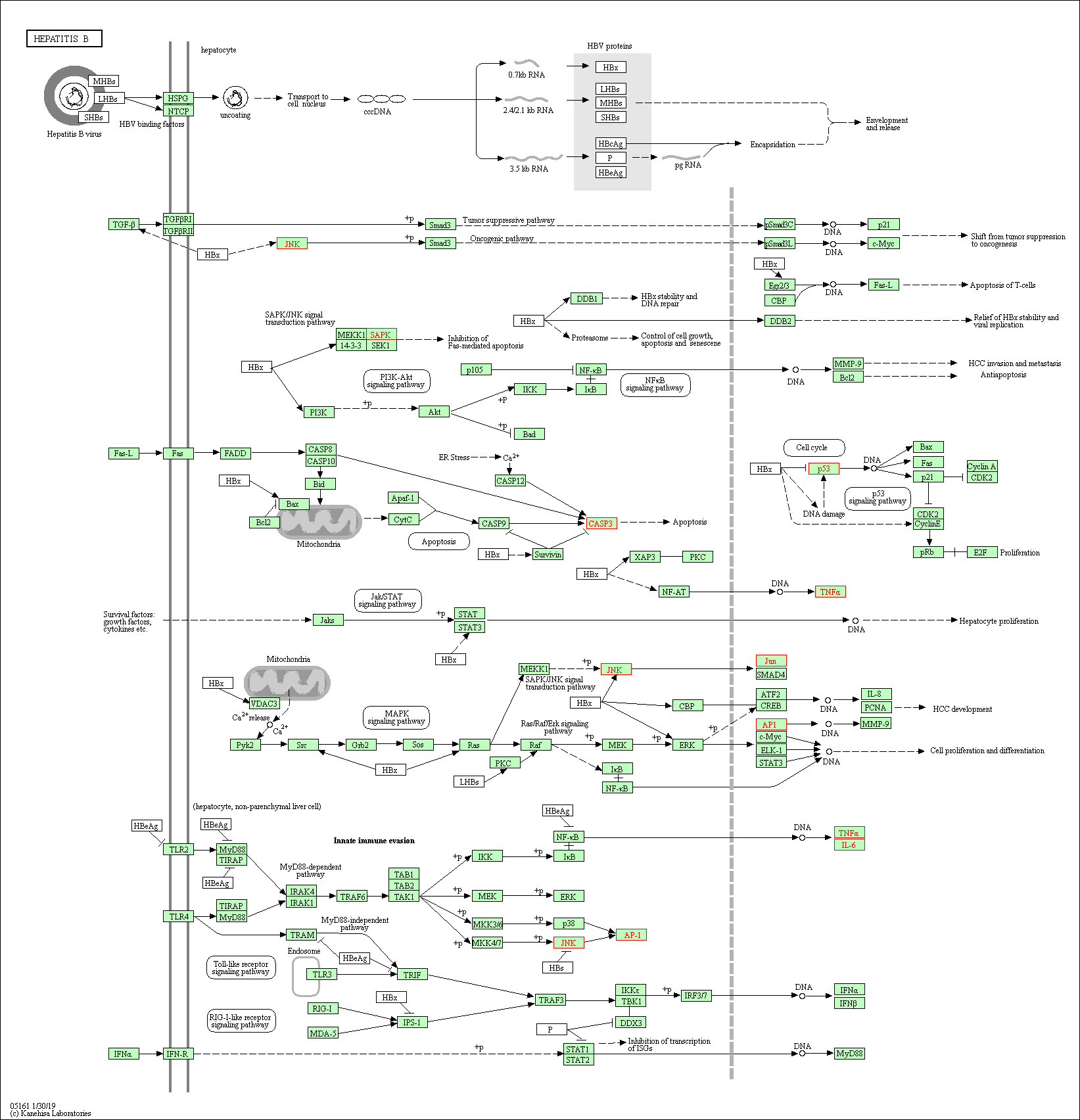

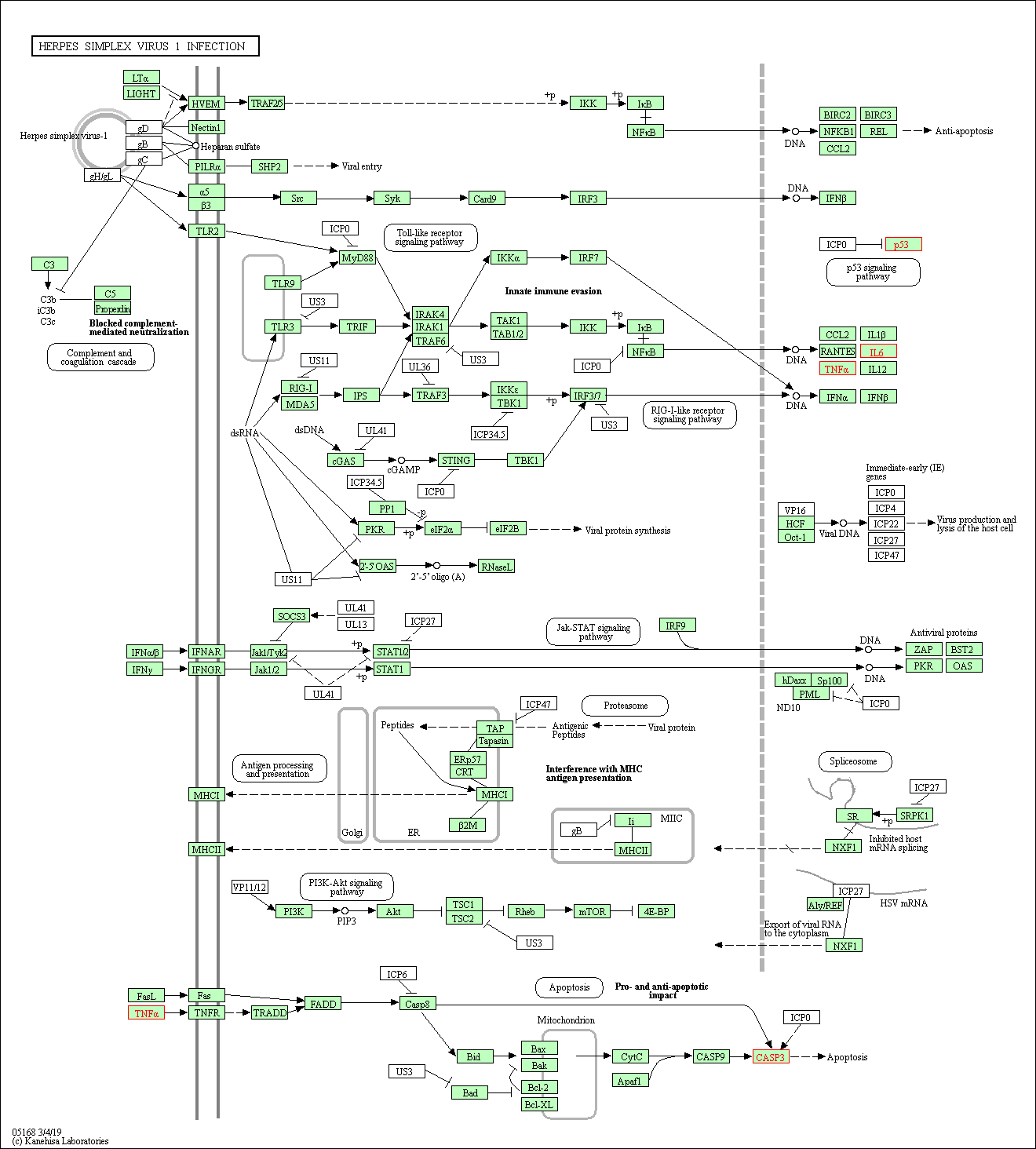

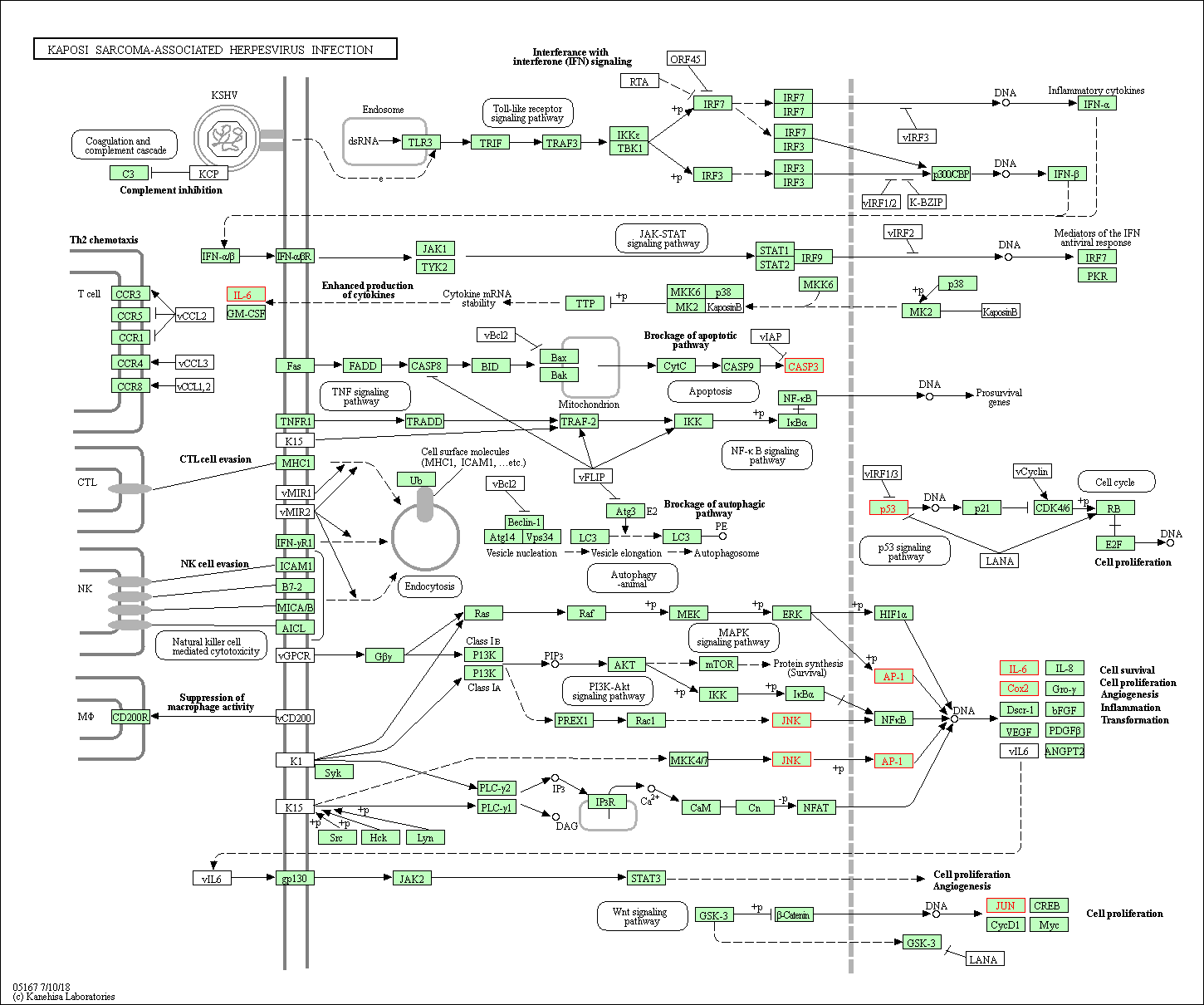

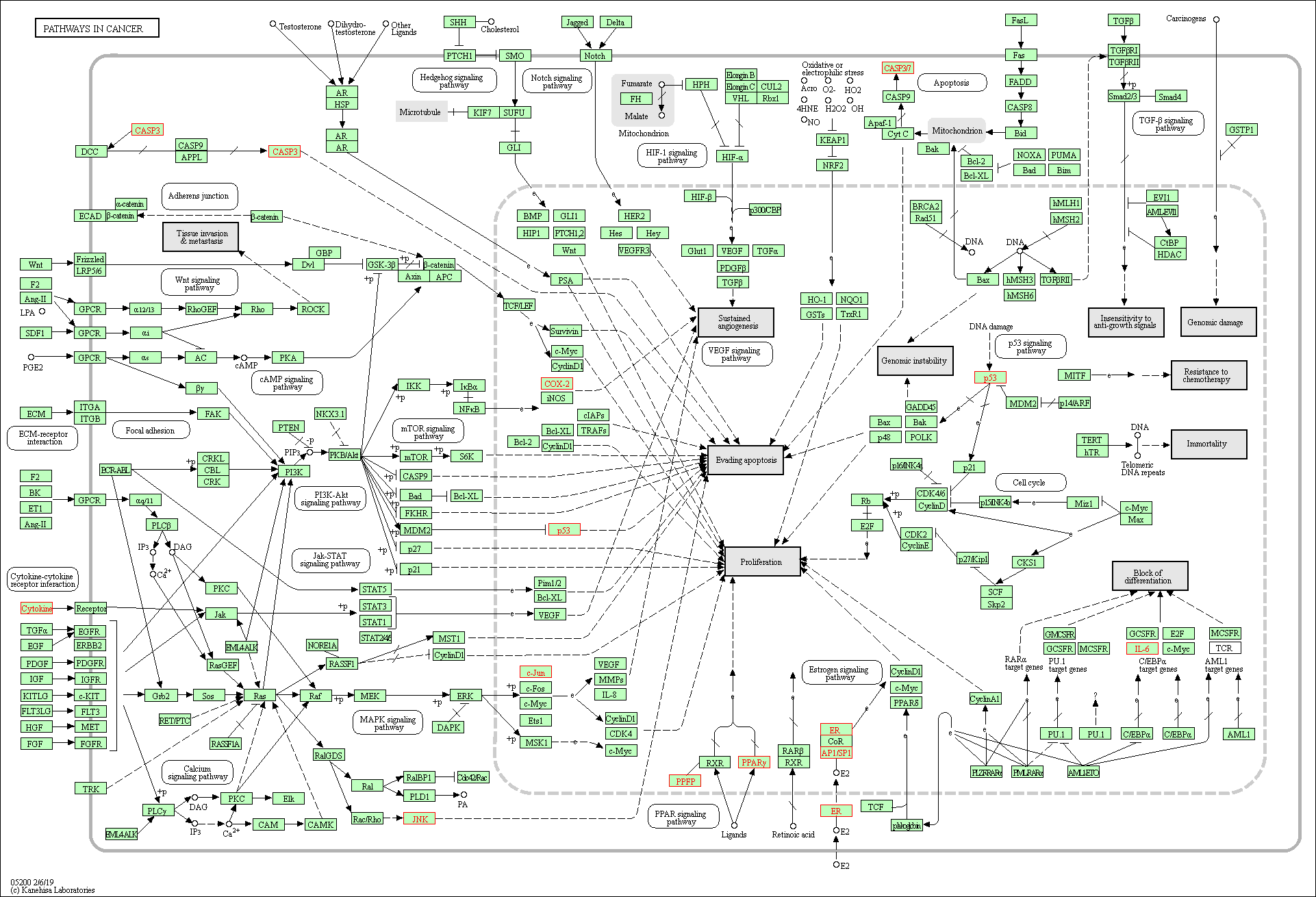

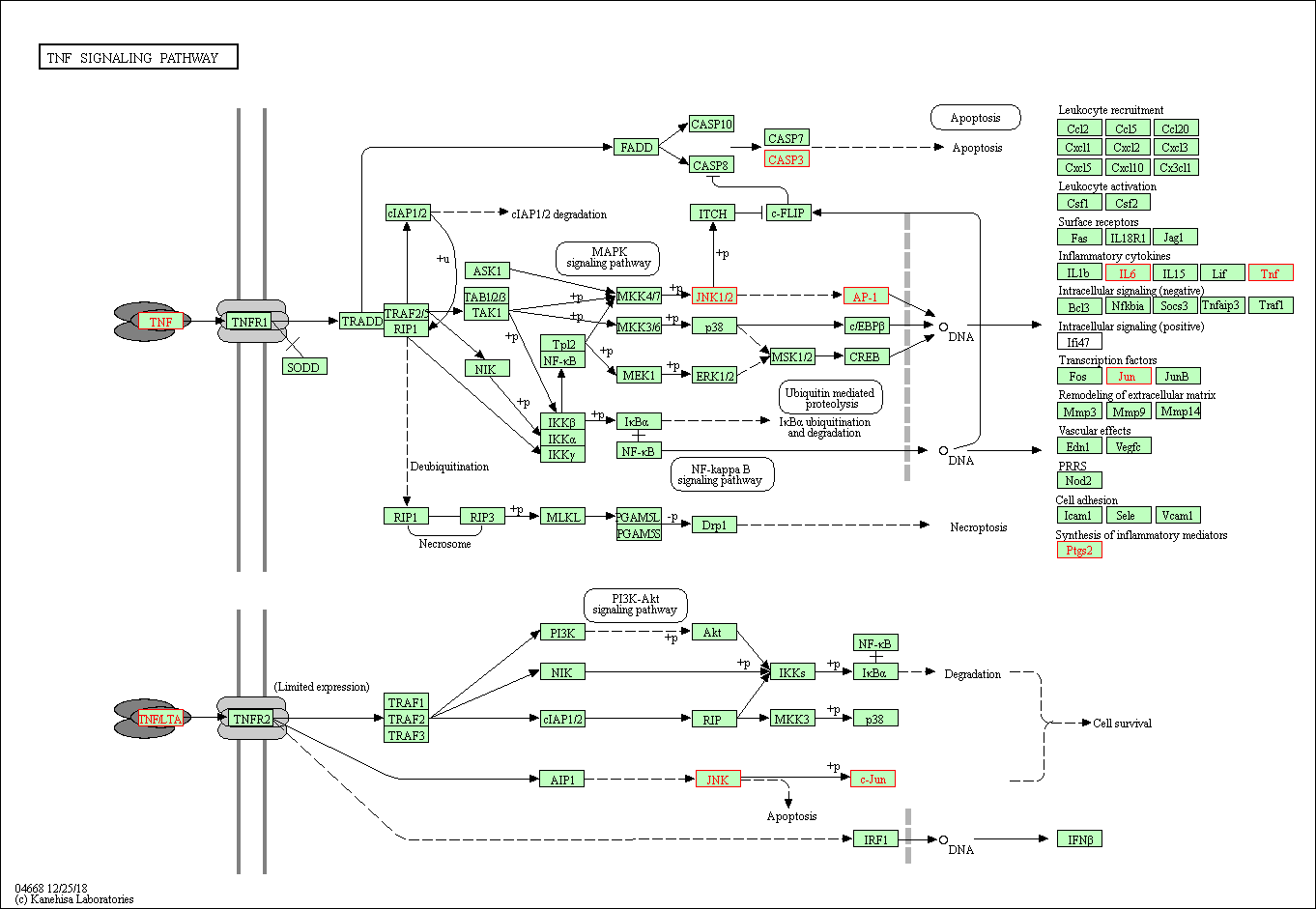

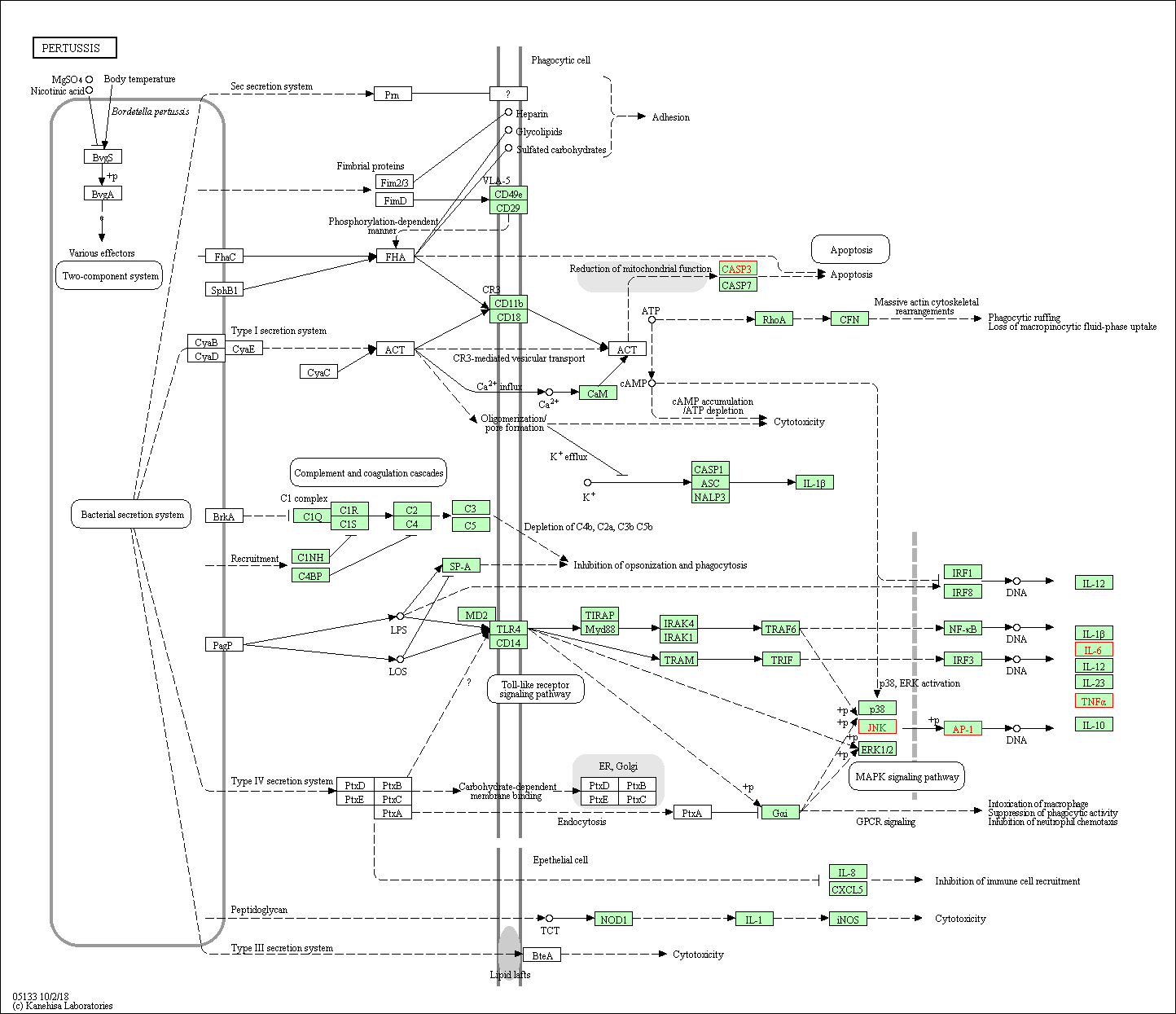

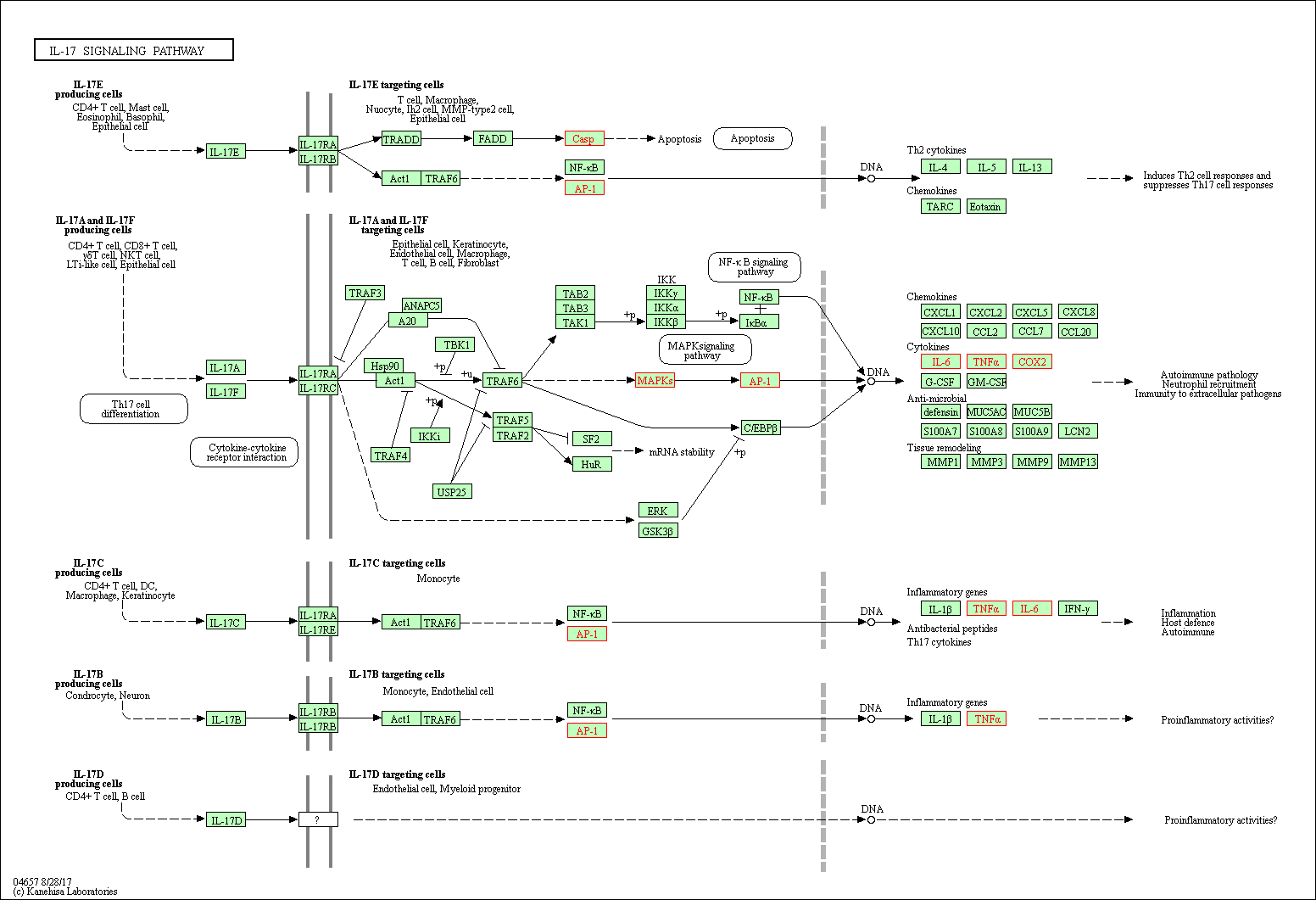

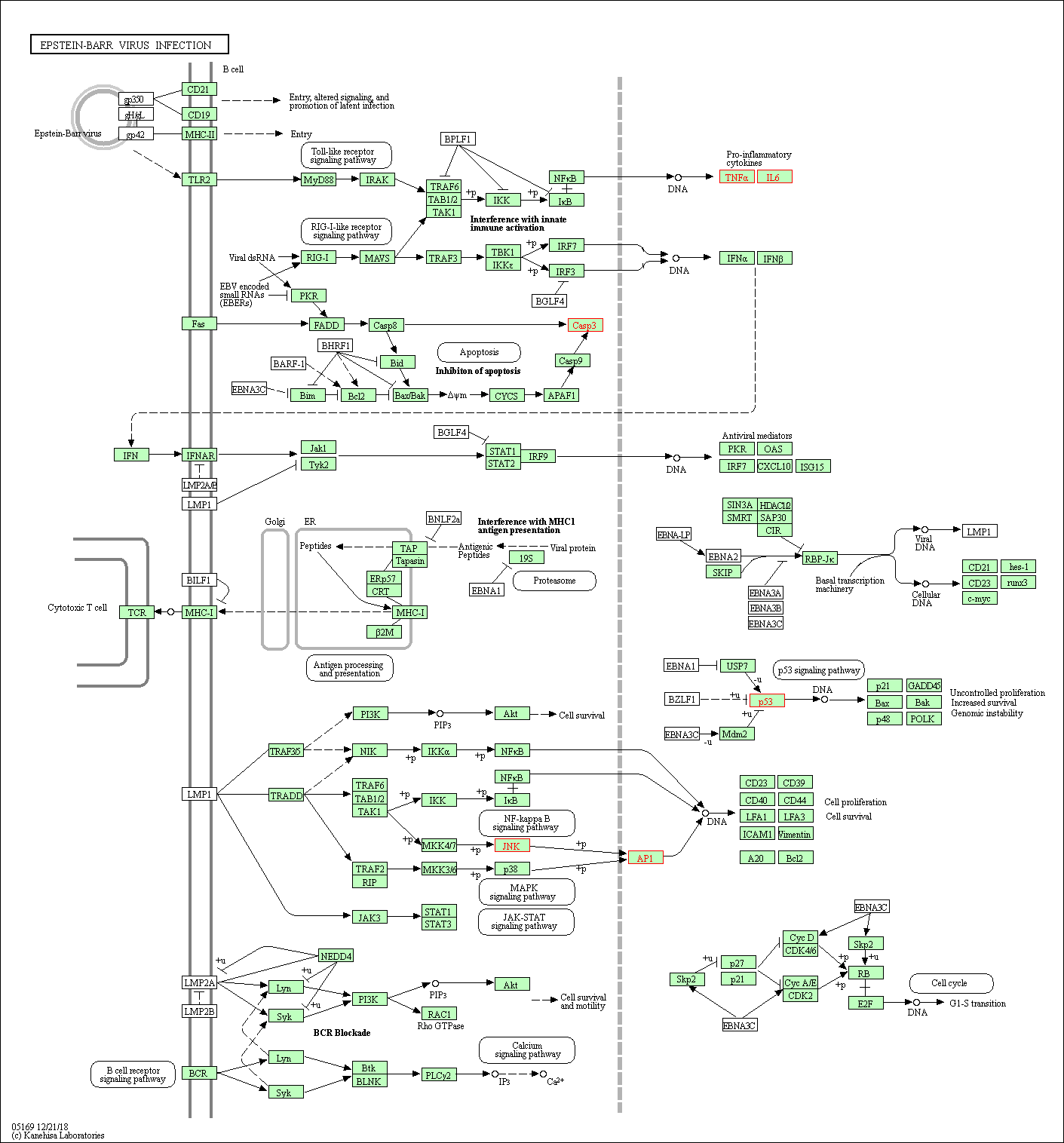

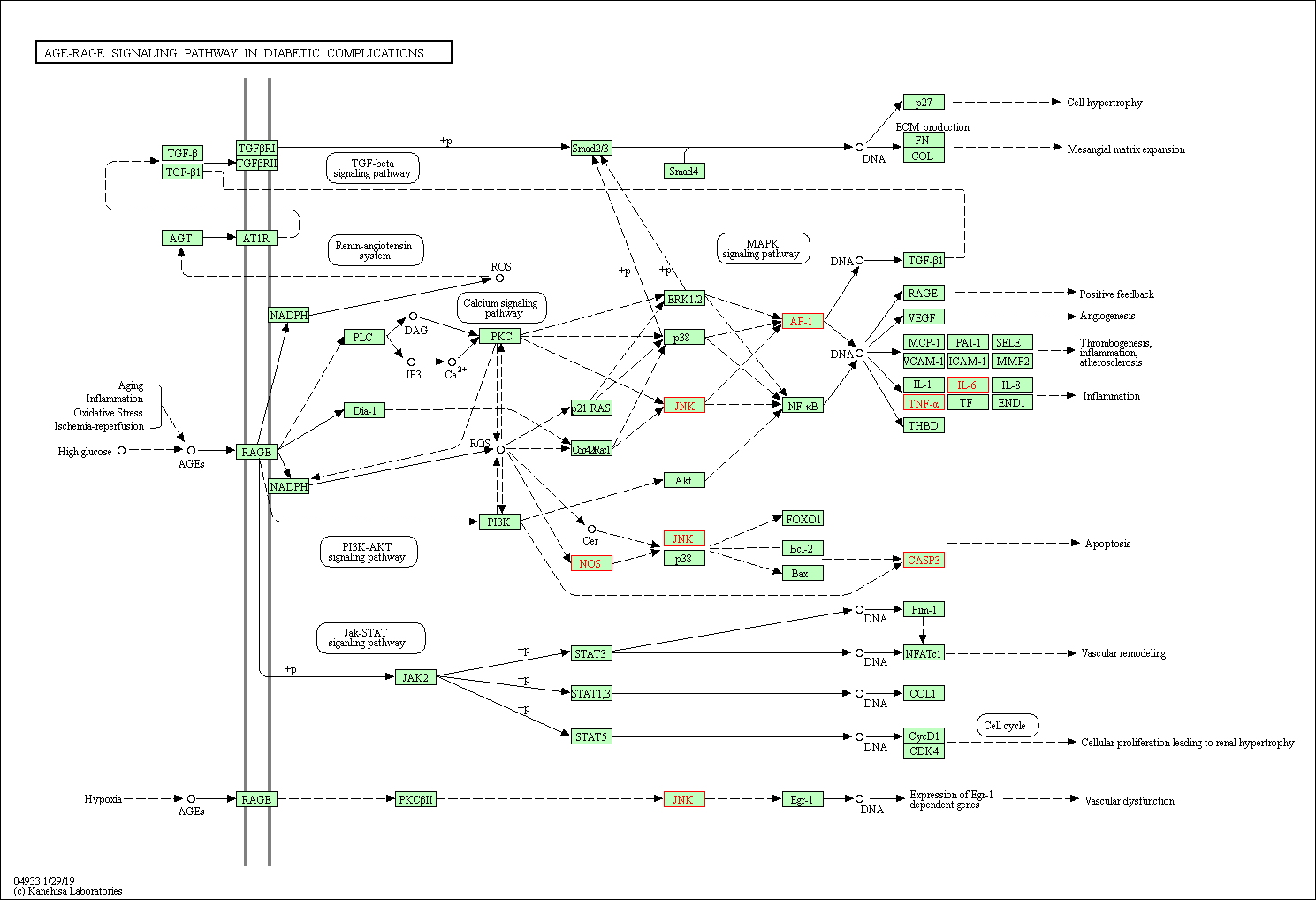

Supplement: Supplementary file 3 — Additional file 3. File for all pictures. [file 12906_2020_2925_MOESM3_ESM.doc]
