# Supplementary material for: Uncovering the mechanism of the effects of Paeoniae Radix Alba on iron-deficiency anaemia through a network pharmacology-based strategy
Source: BMC Complement Med Ther. 2020 Apr 28;20:130. doi: 10.1186/s12906-020-02925-4 (PMC7189569; doi:10.1186/s12906-020-02925-4)
Supplement: Supplementary file 4 — Additional file 4. File for partial targets with components docking. [file 12906_2020_2925_MOESM4_ESM.pdf]

Molecular docking Verification component-Target-score

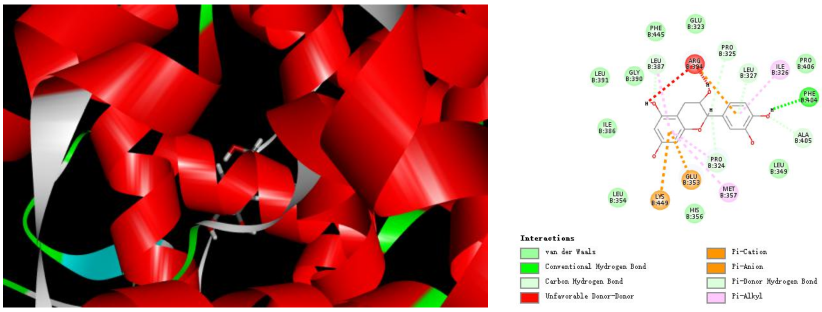

(+)-catechin-ESR1-109.443

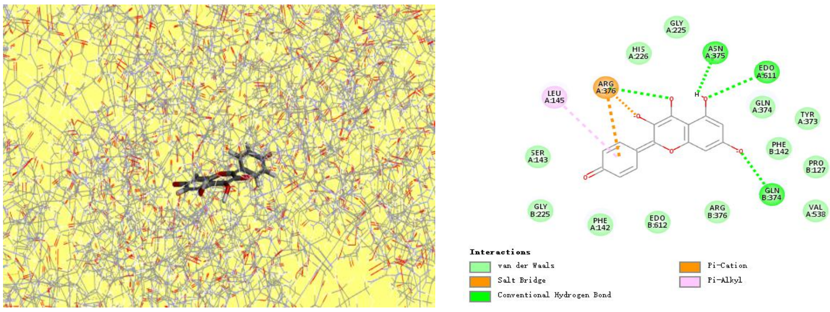

Kaempferol-PTGS2-86.3227

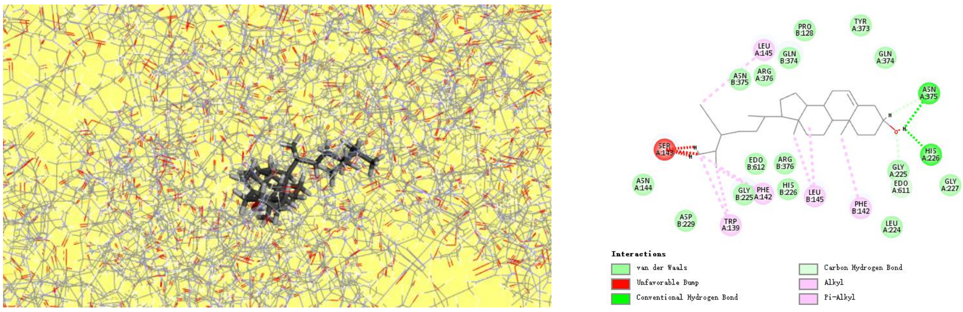

beta-sitosterol-PTGS2-104.549

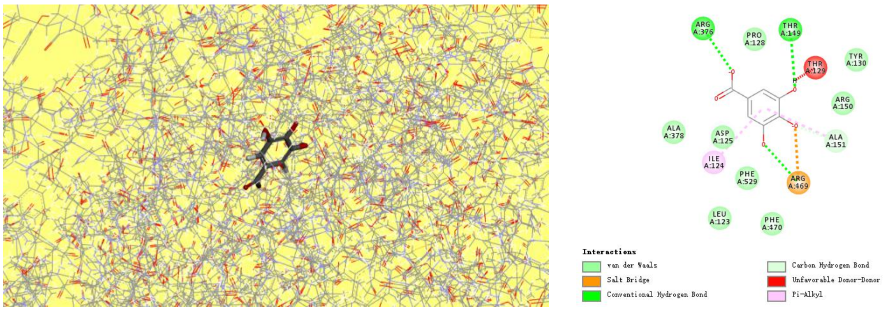

gallic acid-PTGS2-81.4532

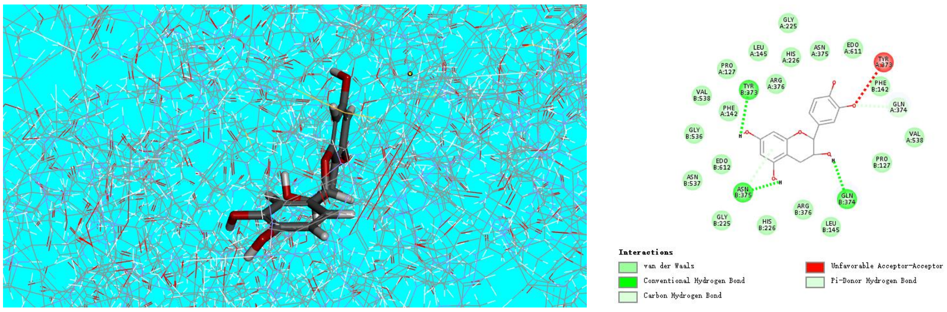

(+)-catechin-PTGS2-103.414

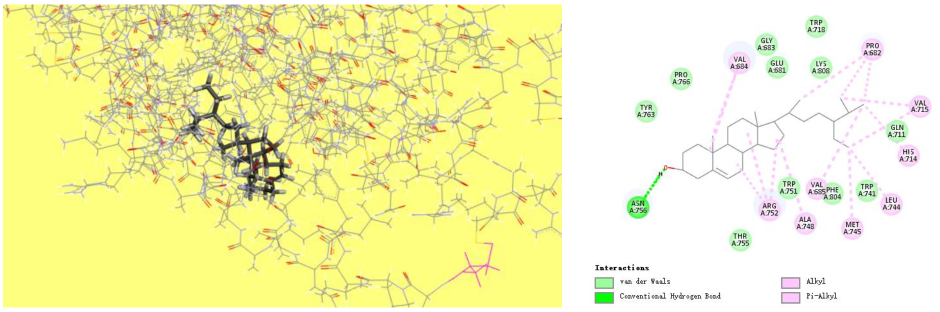

Sitosterol-AR-114.949

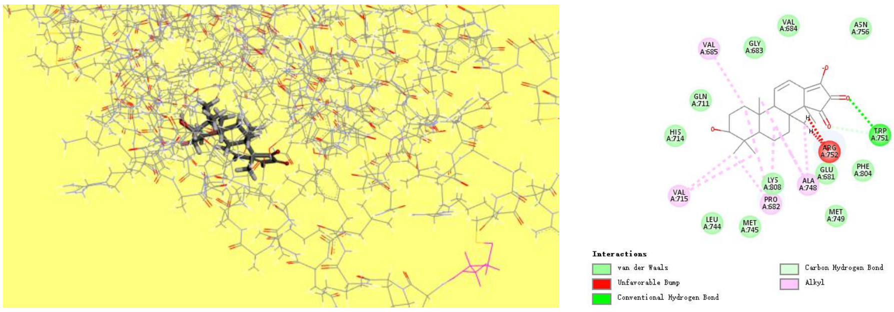

3S-AR-83.5991

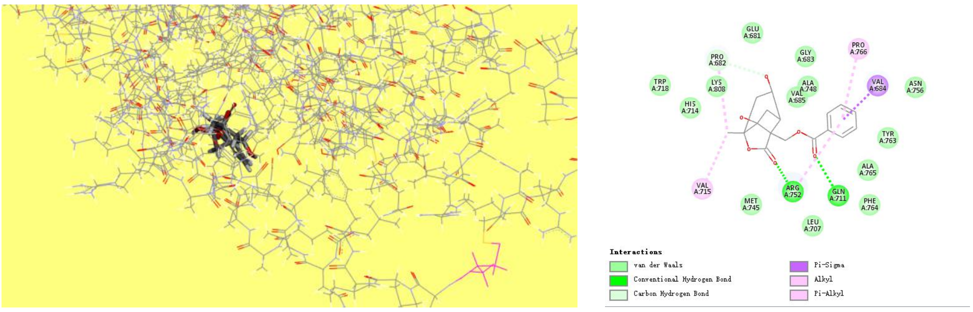

albiflorin_qt-AR-120.185

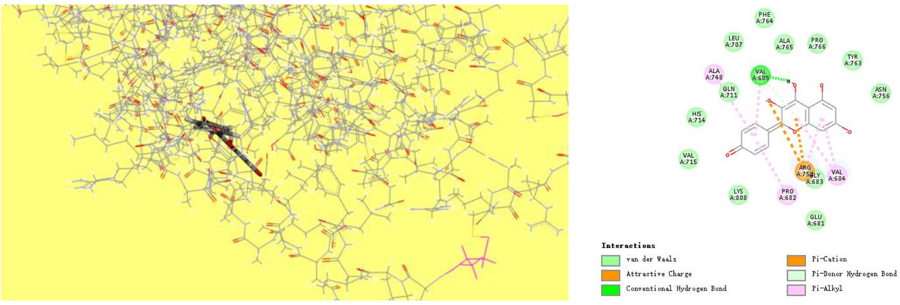

Kaempferol-AR-116.574

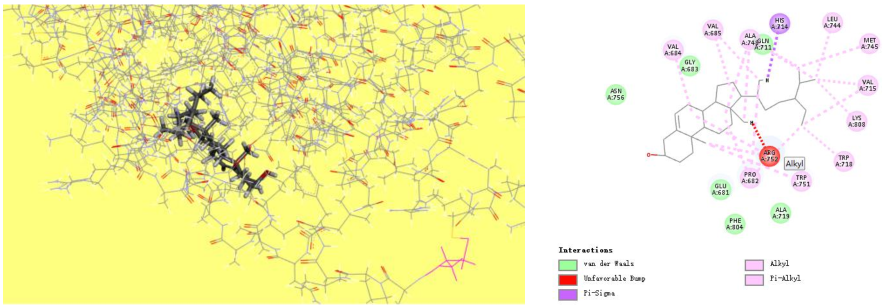

beta-sitosterol-AR-106.703

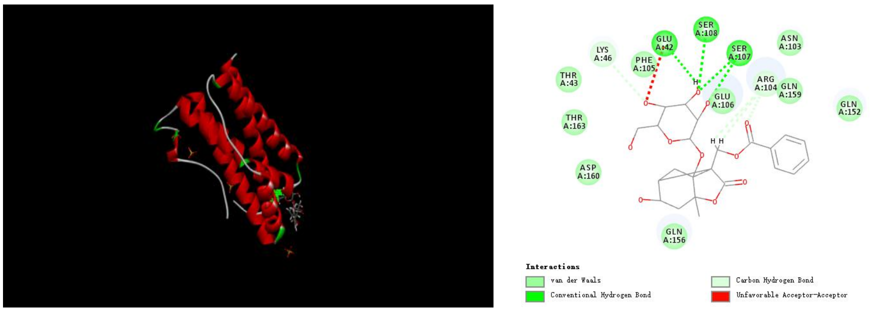

Albiflorin-IL6-118.385

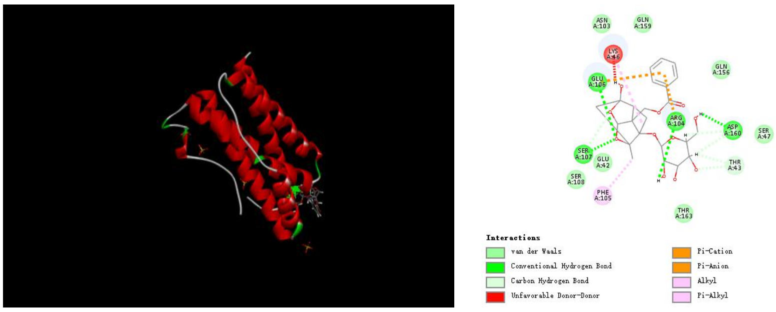

Paeoniflorin-IL6-123.033

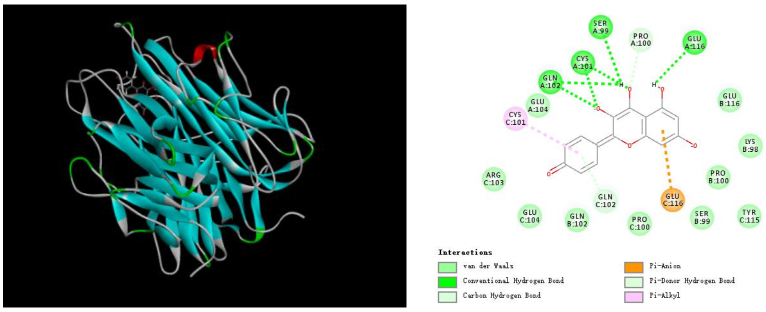

Kaempferol-TNF-107.571

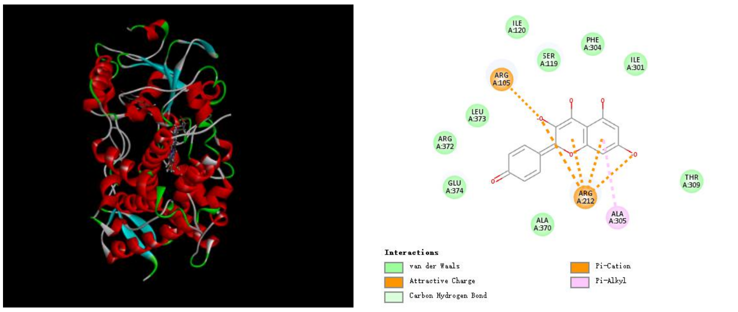

Kaempferol-CYP3A4-119.055

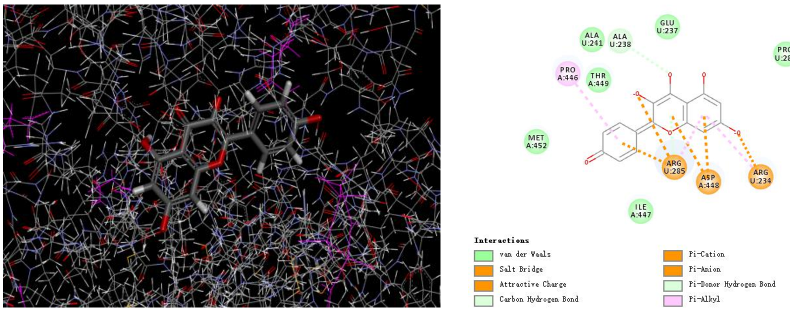

Kaempferol-PPARG-98.3622

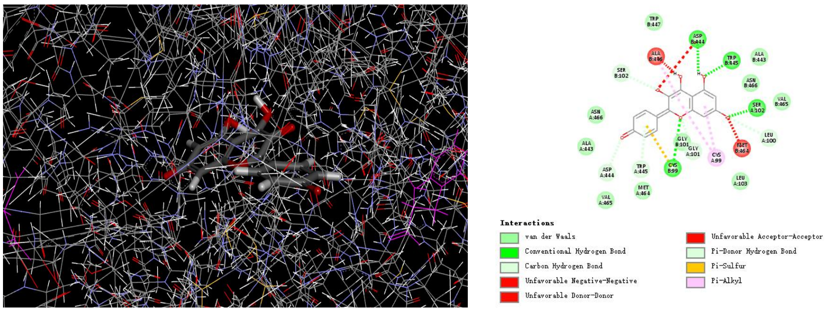

Kaempferol-NOS3100.734

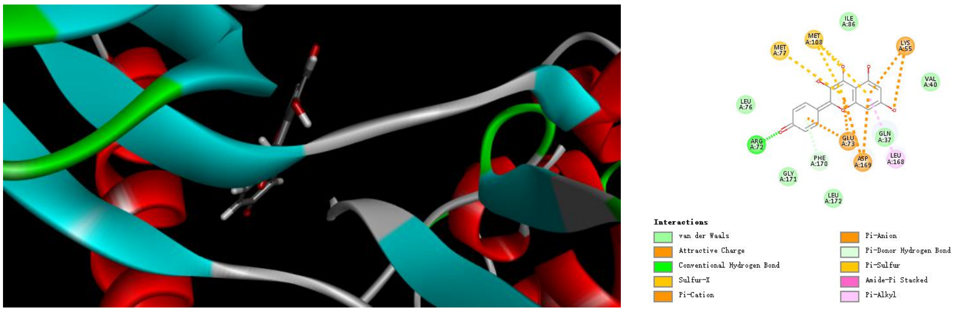

Kaempferol-MAPK8-109.54

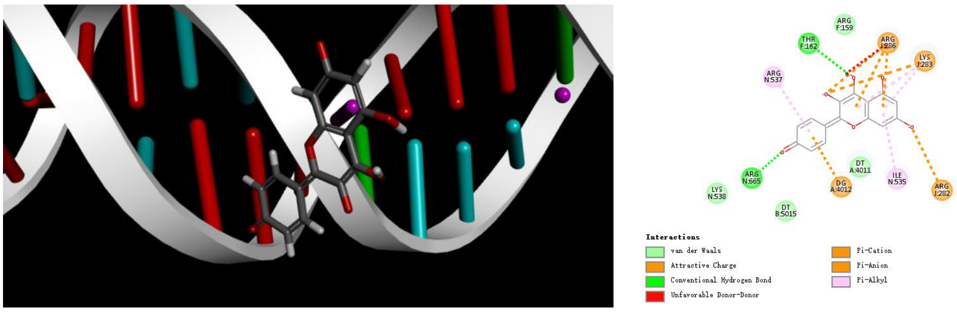

Kaempferol-JUN-89.7769

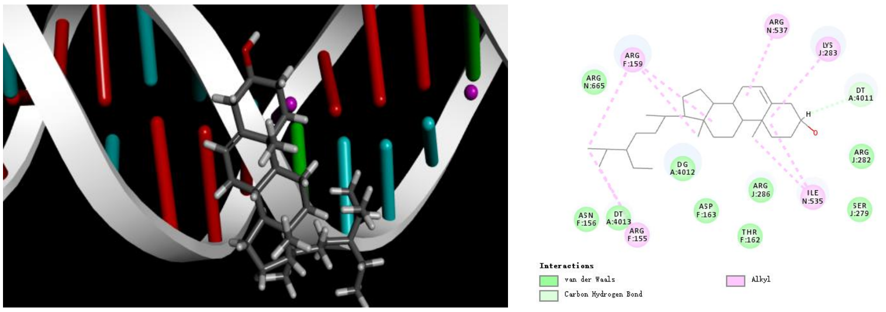

beta-sitosterol-JUN-109.879

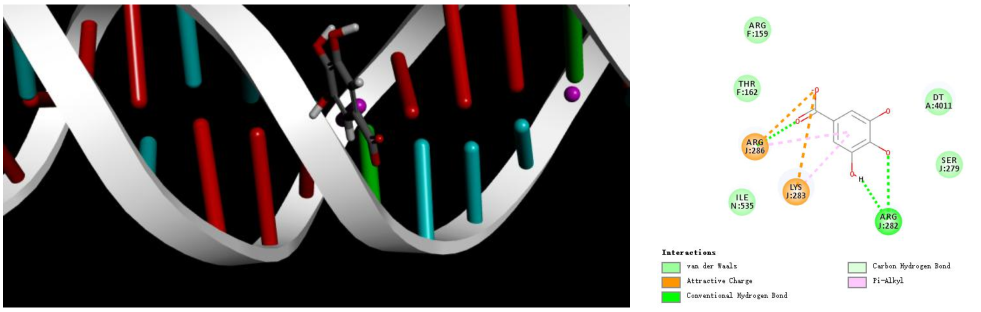

gallic acid-JUN-67.2373

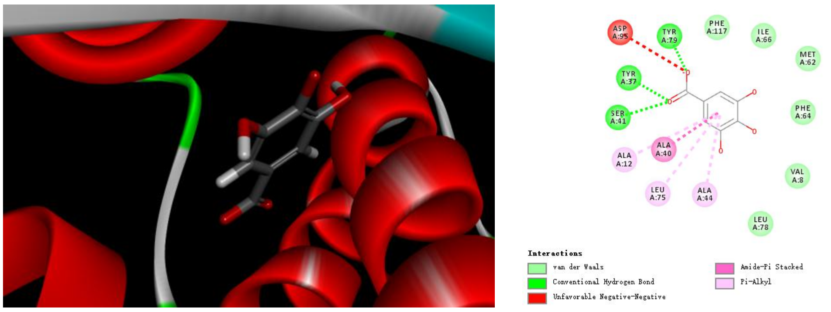

gallic acid-SERPINE1-59.283

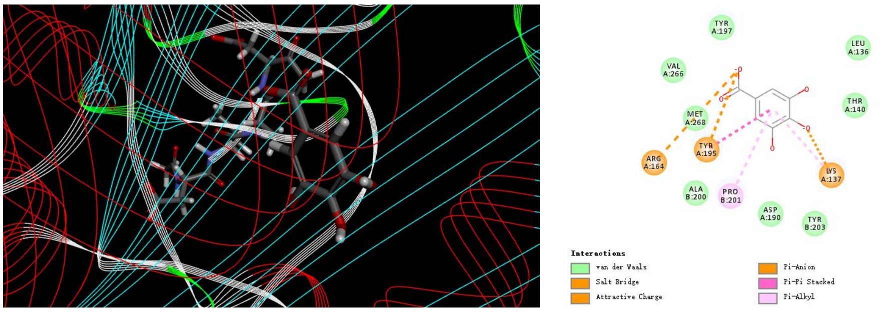

gallic acid-CASP3-66.1591

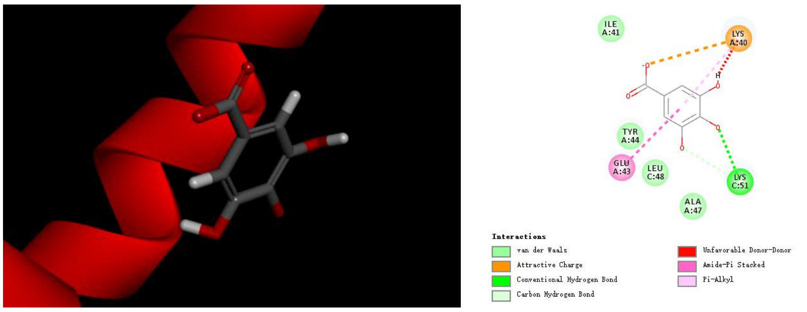

gallic acid-TP53-56.1329

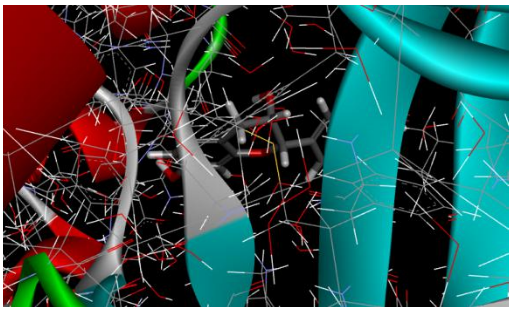

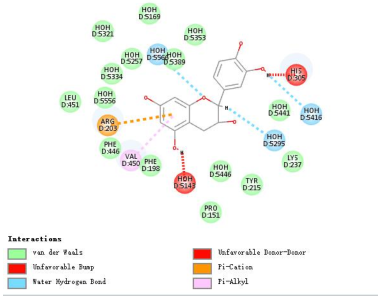

(+)-catechin-CAT-67.2883

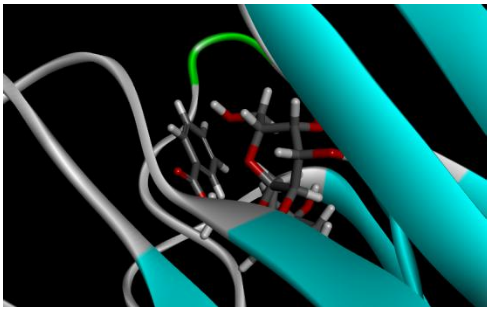

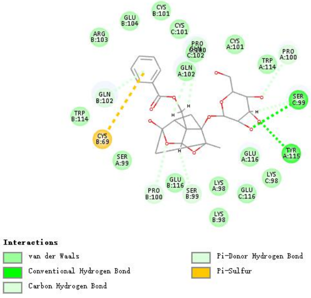

Paeoniflorin-TNF-150.725
